# MicroRNA biomarkers of type 2 diabetes: evidence synthesis from meta-analyses and pathway modelling

**DOI:** 10.1007/s00125-022-05809-z

**Published:** 2022-10-21

**Authors:** Hongmei Zhu, Siu-wai Leung

**Affiliations:** 1 State Key Laboratory of Quality Research in Chinese Medicine, Institute of Chinese Medical Sciences, University of Macau, Macao, China; 2 Present Address: Centre of Gastrointestinal and Minimally Invasive Surgery, Department of General Surgery, Third People’s Hospital of Chengdu, Chengdu, China; 3 Present Address: Medical Research Centre, Third People’s Hospital of Chengdu, Affiliated Hospital of Southwest Jiaotong University, Chengdu, China; 4 Edinburgh Bayes Centre for AI Research in Shenzhen, College of Science and Engineering, University of Edinburgh, Edinburgh, UK

**Keywords:** Meta-analysis, MicroRNA biomarkers, MicroRNA-regulated pathway, Type 2 diabetes

## Abstract

**Aims/hypothesis:**

MicroRNAs are being sought as biomarkers for the early identification of type 2 diabetes. This study aimed to synthesise the evidence from microRNA–type 2 diabetes association studies and microRNA-regulated type 2 diabetes pathway delineation studies that met stringent quality criteria to identify and validate microRNAs of both statistical and biological significance as type 2 diabetes biomarkers.

**Methods:**

Eligible controlled studies on microRNA expression profiling of type 2 diabetes were retrieved from PubMed, ScienceDirect and Web of Science. MicroRNA-regulated type 2 diabetes pathway delineation studies were conducted by integrating and cross-verifying the data from miRTarBase, TransmiR, miRecords, TargetScanHuman, the Kyoto Encyclopedia of Genes and Genomes (KEGG) and the Retraction Watch database. Before meta-analysis, quality assessment was performed according to the corresponding reporting guidelines for evidence-based medicine. To select the most statistically significant microRNAs, we conducted extensive meta-analyses according to the latest methodology. Subgroup and sensitivity analyses were carried out to further examine the microRNA candidates for their tissue specificity and blood fraction specificity and the robustness of the evidence. Signalling pathway impact analysis of dysregulated microRNAs identified from meta-analyses was performed to select biologically significant microRNAs that were enriched in our newly built microRNA-regulated pathways.

**Results:**

Of the 404 differentially expressed microRNAs identified in the 156 controlled profiling studies with a combined sample size of >15,000, only 60 were both consistently and significantly dysregulated in human type 2 diabetes. No microRNAs were both consistently and significantly dysregulated in multiple tissues according to subgroup analyses. In total, 58 microRNAs were found to be robust in sensitivity analyses. A total of 1966 pathway delineation studies were identified, including 3290 microRNA–target interactions, which were further combined with KEGG pathways, producing 225 microRNA-regulated pathways. Impact analysis found that 16 dysregulated microRNAs identified from extensive meta-analyses were statistically significantly enriched in the augmented KEGG type 2 diabetes pathway.

**Conclusions/interpretation:**

Sixteen microRNAs met the criteria for biomarker selection. In terms of both significance and relevance, the order of priority for verification of these microRNAs is as follows: miR-29a-3p, miR-221-3p, miR-126-3p, miR-26a-5p, miR-503-5p, miR-100-5p, miR-101-3p, mIR-103a-3p, miR-122-5p, miR-199a-3p, miR-30b-5p, miR-130a-3p, miR-143-3p, miR-145-5p, miR-19a-3p and miR-311-3p.

**Registration:**

PROSPERO registration number CRD42017081659.

**Graphical abstract:**

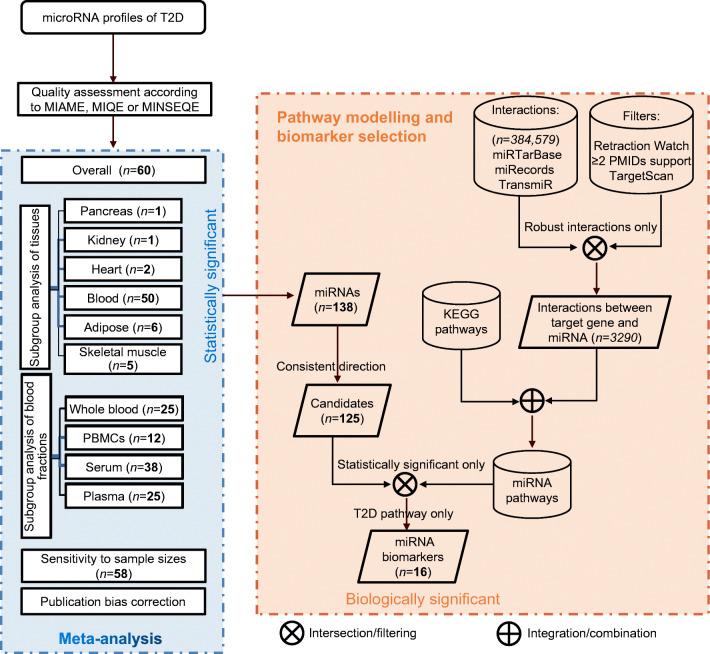

**Supplementary Information:**

The online version contains peer-reviewed but unedited supplementary material available at 10.1007/s00125-022-05809-z.

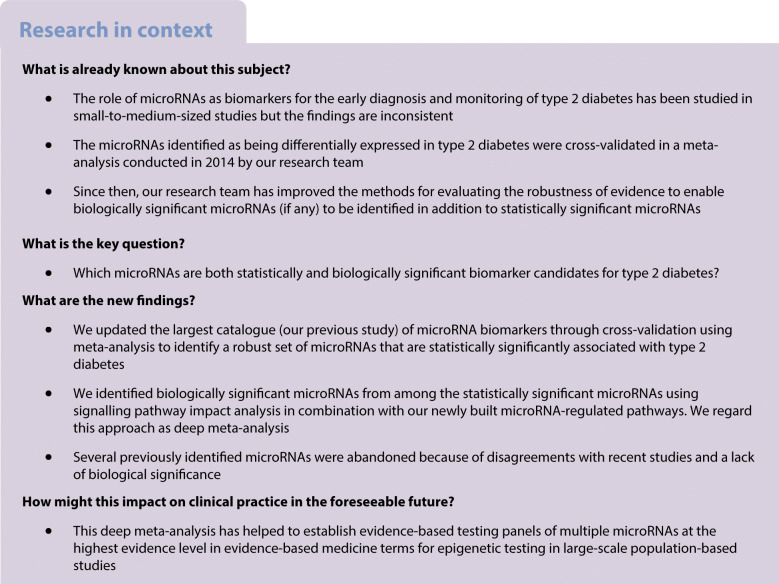



## Introduction

In 2021, 537 million adults were living with diabetes and 240 million people with diabetes were undiagnosed according to the International Diabetes Federation [1]. Type 2 diabetes is characterised by insulin resistance and beta cell dysfunction [2]. Uncontrolled type 2 diabetes can lead to a number of serious health problems, including microvascular and macrovascular complications, which affect the heart, blood vessels, eyes, kidneys and nerves [3] and cause a huge burden on healthcare systems (at least US$966 billion in health expenditure in 2021) [1]. Because of late diagnosis and intervention, the burden of type 2 diabetes is getting heavier and heavier [4]. Earlier diagnosis of diabetes through molecular medicine approaches, such as monitoring type 2 diabetes-specific microRNAs, would enable earlier intervention in type 2 diabetes [5].

MicroRNAs are stable and endogenous non-coding RNAs of approximately 22 nucleotides whose abnormal expression is associated with disease [6, 7], including type 2 diabetes [5]. Our pilot study of microRNA biomarkers in type 2 diabetes [8] found that studies on differentially expressed microRNAs are heterogeneous and produce inconsistent results; thus, they were subject to evaluation by evidence-based meta-analysis. In meta-analysis the results across studies with similar protocols and objectives are statistically synthesised, for example to increase statistical power, obtain a more precise estimation of effect sizes, explore heterogeneity or risks, and generalise the results across studies [9, 10]. Our pilot study published in early 2015 introduced stringent quality assessment and used the latest meta-analysis methods, replacing the obsolete vote-counting method [8]. While many studies on type 2 diabetes and other diseases have followed our approach, some meta-analyses published since 2015 still have methodological flaws. Our research protocol, published in 2021, reported a 5.5-fold increase from 2014 to 2020 in the number of microRNA studies carried out on type 2 diabetes [11]. We therefore performed this comprehensive meta-analysis to fill the gaps and confirm which microRNAs are reliably associated with type 2 diabetes. To establish a genetic testing panel of specific microRNAs for early type 2 diabetes diagnosis, we assessed their deeper biological relevance by pathway analysis after confirming their statistical significance. Research indicates that popular pathway analysis tools, such as the Database for Annotation, Visualization, and Integrated Discovery (DAVID) [12] and DIANA–mirPath [13], are not free from biases and inaccuracies [14]. We adopted a state-of-the-art workflow for microRNA pathway analysis using microRNA-augmented pathways (mirAP) to integrate microRNA into signalling pathways and using impact analysis to obtain information on pathway topology [15]. As most of the current literature reporting experimentally validated microRNA–target interactions is based on mirAP from mirTarBase version 4.5, rather than the latest version (version 9.0) [16], the data are outdated. In addition, problematic data from retracted studies were found in mirTarBase, which had not been processed during the generation of mirAP. Therefore, we constructed new microRNA pathways with microRNA–target interactions according to the latest databases, including mirTarBase. New quality control criteria were introduced to ensure the quality of the evidence, for example to ensure that microRNA studies that have been retracted or that have concerns over research integrity were excluded.

## Methods

The study protocol was registered in PROSPERO following the PRISMA guidelines (registration number CRD42017081659) and published in *PLOS ONE* in 2021 [11].

### Search strategies

PubMed, ScienceDirect and Web of Science were searched for type 2 diabetes microRNA expression profiling studies published between 1993 and 2020 using the terms: (‘miRNA’, ‘diabetes’ and ‘expression’ in Title/Abstract) or (‘miRNA’, ‘diabetes’ and ‘profil*’ in Title/Abstract) or (‘microRNA’, ‘diabetes’ and ‘expression’ in Title/Abstract) or (‘microRNA’, ‘diabetes’ and ‘profil*’ in Title/Abstract). The last search was conducted on 27 July 2020. Human microRNAs are usually expressed as ‘microRNA-*’ or ‘miR-*’ according to standard nomenclature. Those discovered before the standard nomenclature was established will retain their original names, such as let-7 [17]; thus, the Retraction Watch database was searched in September 2021 using the terms ‘miR*’ OR ‘microRNA*’ OR ‘let-7*’ in the title to filter out retracted studies on microRNAs.

### Eligibility criteria and study selection

Eligible studies had to meet the following inclusion criteria: (1) investigation of differentially expressed microRNAs (DEMs) in people with type 2 diabetes; (2) identification of DEMs in diabetic and non-diabetic control samples; (3) reported microRNA detection technology; (4) reported criteria for selecting DEMs; (5) reported sample sizes; and (6) not be retracted or have concerns over research integrity. Studies on the identification of DEMs in saliva or urine were excluded, as this study focused on microRNAs in blood.

### Data extraction and quality assessment

The items collected and recorded from eligible studies included study ID (i.e. first author and year of publication), location of study, tissue types, clinical information on type 2 diabetes (i.e. status, age, BMI, HbA_1c_), sample size, microRNA detection platform used, criteria used for selecting DEMs, and the list of DEMs and their corresponding fold changes (if available). DEMs were aligned with miRBase version 22 [18] to unify the names before quality assessment. Quality assessment was performed according to the reporting guidelines for the respective platform, that is, the Minimum Information About a Microarray Experiment (MIAME) guideline (version 2.0) [19], the Minimum Information for Publication of Quantitative Real-time PCR Experiments (MIQE) guideline [20] and the Minimum Information About a high-throughput SEQuencing Experiment (MINSEQE) guideline (https://www.fged.org/projects/minseqe, accessed 2 September 2022) were used for evaluating microarray, PCR and RNA-Seq studies, respectively. Six domains were assessed with the MIAME or MIQE guideline and five domains were assessed with the MINSEQE guideline [11]. Domains were rated as low risk, unclear risk or high risk, suggesting high reproducibility, ambiguous reproducibility and low reproducibility, respectively.

### Data analysis

Extensive meta-analyses were performed in R with the metafor package [21] under a random-effects model. Both empirical Bayes (EB) estimation and restricted maximum likelihood (REML) estimation were used to estimate the outcomes of the meta-analyses. The outcomes were presented as absolute values of log_*e*_ (odds ratios) (logORs) with adjusted *p* values and 95% CIs, based on the numbers of dysregulation events in both type 2 diabetic and non-diabetic control samples. MicroRNAs with logORs >0 or <0 were considered to be upregulated or downregulated, respectively. Bonferroni correction was used to adjust *p* values, and microRNAs identified by both REML and EB estimation methods with adjusted *p* values <0.05 were considered to be significant differentially expressed microRNAs in this meta-analysis.

### Subgroup and sensitivity analyses

Subgroup analyses were performed on tissue type (e.g. muscle, adipose tissue), blood fraction (e.g. serum, plasma) and microRNA detection method (PCR-based and RNA-Seq) to investigate potential heterogeneity. When examining tissue type, the tissue type in studies using different blood fractions was classified as blood, as these studies aimed to investigate circulating microRNAs in blood. Similarly, the tissue type in studies using whole pancreas or pancreatic islets was classified as pancreatic tissue.

Sensitivity analysis based on sample size was carried out to test the robustness of the findings. Meta-analyses were repeated on studies with sample sizes ≥25 and ≥50.

### Publication bias

Publication bias is the phenomenon that a study with positive results and/or statistically significant outcomes is more likely to be published than a study with negative results or non-statistically significant outcomes. This bias can misinform and mislead researchers [22]. Funnel plots were generated to visualise possible publication bias, and Begg’s [23] and Egger’s [24] tests were carried out to detect the significance of any publication bias. The trim-and-fill method [25], which estimates the lack of studies on one side of the funnel plot, was performed to correct publication bias and only microRNAs with statistically significant effect sizes after correction were considered for selection as biomarkers.

### MicroRNA pathway modelling and biomarker selection

Few microRNA pathway databases and pathway studies include microRNA interactions that reveal microRNA-regulated pathways as the molecular mechanisms of disease. In this study we used information on microRNA targets and Kyoto Encyclopedia of Genes and Genomes (KEGG) pathways to build a new database of microRNA pathways and integrated these pathways in accordance with their topology to select potential microRNA biomarkers of type 2 diabetes. Experimentally validated interactions between human microRNAs and their target genes were downloaded from the latest versions of the miRTarBase (version 9.0) [16], miRecords (version 4.0) [26] and TransmiR (version 2.0) [27] databases with PMIDs. MicroRNA names were aligned with names in miRBase version 22 [18]. The top predicted microRNA–target pairs were obtained from TargetScanHuman 8.0 [28], which acted as a filter to select interactions that were consistent between experiments and predictions. As mentioned previously, the problematic data from retracted studies were found in the above-mentioned experimentally validated databases. We excluded the retracted studies in accordance with the Retraction Watch database to ensure the quality of our database of microRNA–target interactions. In other words, each of the interactions had to be supported by at least two different information sources (PMIDs) and two types of database (i.e. wet experimentation and bioinformatics prediction) and to have not been retracted (according to the Retraction Watch database) or not be associated with concerns over research integrity. R package ROntoTools [29] was adopted to obtain the latest KEGG pathways, which were further combined with robust microRNA–target interactions using mirIntegrator [30] to produce microRNA-regulated pathways. Signalling pathway impact analysis [31] of dysregulated microRNAs from meta-analyses was performed to select biologically significant microRNAs that were enriched in our newly built microRNA-regulated pathways. Laterza et al [32] have demonstrated how circulating microRNAs may indicate the physiological state at tissue level. They are stable and can be detected by less invasive techniques [33] and are specific to particular disease states [34]. Therefore, circulating microRNAs of statistical significance (in different analyses) and biological relevance (especially enriched in type 2 diabetes pathways) and detectable in blood or blood fractions were prioritised for selection as type 2 diabetes biomarkers.

## Results

### Included studies and their characteristics

**Fig. 1:**
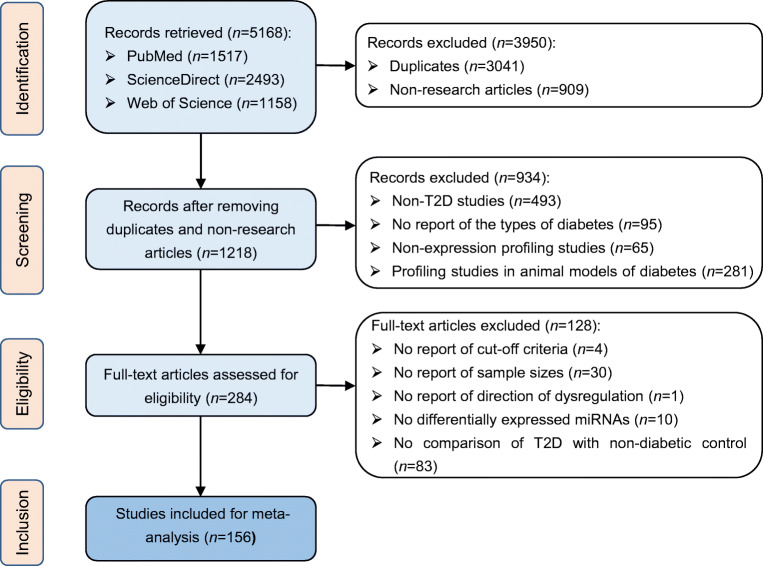
Flow diagram of study selection. T2D, type 2 diabetes

Figure 1 shows the study selection process. A total of 5168 potentially relevant records were identified from PubMed, ScienceDirect and Web of Science. After removal of duplicate publications and non-research articles such as reviews, 1218 records remained, of which 284 were identified for full-text assessment. During the full-text assessment, 128 studies were excluded, for example for not reporting cut-off criteria, sample sizes or direction of dysregulation. As a result, 156 studies met the eligibility criteria for meta-analysis, with a combined sample size of >15,000. Of the 156 eligible studies (listed in electronic supplementary material [ESM] Appendix), most reported only type 2 diabetes microRNA expression profiles in humans; approximately 30 studies were based on both individuals with type 2 diabetes and animals models of diabetes. For the present meta-analysis, only human data were used. Details of the study characteristics are shown in ESM Table 1.

### Quality assessment

**Fig. 2:**
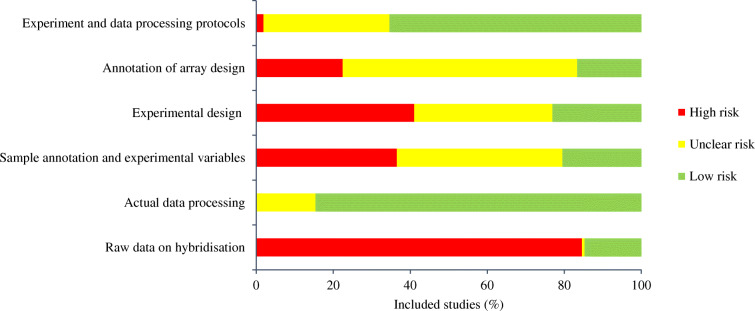
Quality assessment according to the MIAME guideline

The MIAME guideline 2.0 [19], MINSEQE guideline and MIQE guideline [20] were used to assess study quality. Figure 2 shows the results of the quality assessment process, mainly according to the domains of the MIAME guideline. The detailed quality assessment of the individual studies is shown in ESM Table 2. The overall assessment found that 85% of the included studies did not report raw data on hybridisation, which was rated as high risk, and only 17% and 23% of studies provided sufficient information (for replicability and reproducibility) about annotation of array design and experiment design, respectively (Fig. 2).

### Meta-analysis of differentially expressed microRNAs

The outcomes from REML estimation of DEMs are presented in the main text and ESM Tables 3–21, while the outcomes from EB estimation are presented in ESM Tables 22–35. Of the 404 DEMs reported in the 156 studies that compared type 2 diabetic samples with non-diabetic control samples, 205 (51%) were reported in at least two substudies. Among the 205 DEMs, meta-analysis identified 60 statistically significant dysregulated DEMs (i.e. 31 upregulated and 29 downregulated), as shown in ESM Table 3; the remaining 145 DEMs were of no statistical significance (adjusted *p*>0.05). The most frequently reported upregulated microRNA was miR-320a, which was reported in 14 substudies (logOR 5.2885, 95% CI 2.2857, 8.2914; adjusted *p*=4.96×10^–2^). The most frequently reported downregulated microRNA was MiR-30c-5p (logOR 7.4205, 95% CI 5.7924, 9.0485; adjusted *p*=3.68×10^–17^), which was reported in six substudies.

### Subgroup analysis

A total of 118 of 156 studies investigated circulating microRNAs in plasma, serum, peripheral blood mononuclear cells (PBMCs) or whole blood. Seven studies investigated skeletal muscle tissue, five pancreatic tissue, seven adipose tissue, five blood vessels, five heart, three foot skin, two vitreous of the eye, two kidneys, one epithelial breast cancer tissue, one bone cells, one liver and two gingival crevicular fluid. Among the five pancreatic profiling studies, four studies used pancreatic islets and one used pancreatic tissue. Details are provided in ESM Table 1. Statistically significant dysregulation of microRNAs in different tissue types is shown in ESM Tables 4–9. In total, one statistically significant microRNA was found in the pancreas, one in the kidney, two in the heart, five in skeletal muscle, six in adipose tissue and 50 in blood. No statistically significantly dysregulated microRNAs were identified in foot skin, vitreous and gingival crevicular fluid. In addition, no statistically significantly dysregulated microRNAs were identified in multiple tissues.

In subgroup analysis of blood fractions, 15 studies extracted RNA from PBMCs, 39 studies used serum as the RNA source, 43 studies focused on plasma RNA and 26 studies used whole blood as the RNA source. Subgroup analysis identified 12, 38, 25 and 25 statistically significantly dysregulated microRNAs in PBMCs, serum, plasma and whole blood, respectively (ESM Tables 10–13). ESM Table 14 shows that 87 statistically significant microRNAs were identified from the four RNA sources. Of these, 76 microRNAs were identified in only one of the four RNA sources; 10 microRNAs (e.g. miR-125b-5p and miR-130b-3p) were upregulated in one source but downregulated in another; and one microRNA (miR-150-5p) was upregulated in both whole blood and serum.

In total, 150 studies detected microRNAs using PCR-based methods, three studies used sequencing technologies, two studies screened for microRNAs using sequencing technologies and validated the results using PCR-based methods, and one study used the NanoString assay. Subgroup analyses of microRNAs detected using PCR-based methods and sequencing technologies identified 61 and 11 statistically significantly dysregulated microRNAs, respectively, which are shown in ESM Tables 15 and 16, respectively. Two microRNAs (miR-144-3p and miR-30b-5p) were upregulated in PCR-based studies but downregulated in sequencing-based studies.

### Sensitivity analysis

Sensitivity analysis was conducted to examine the robustness of the findings. We first excluded those studies with sample sizes <25, and then further excluded studies whose sample sizes were <50, after which 114 and 78 studies remained, respectively. Analysis of the 114 and 78 studies identified 53 and 37 microRNAs, respectively, that were significantly differentially expressed (ESM Table 17). Some microRNAs were statistically significantly dysregulated both in sensitivity analysis and in the overall analysis, whereas others were not. For example, miR-93-5p was statistically significant in sensitivity analysis but not in the overall analysis, as several studies with small sample sizes (<25) but large effect sizes were excluded in sensitivity analysis. ESM Table 17 shows that the number of significant microRNAs decreased when the sample size increased. These data indicate that the small sample sizes used in microRNA profiling studies may explain some of the differences seen in the results.

### Publication bias

Funnel plots, Begg’s tests and Egger’s tests were performed to evaluate publication bias. The results of the analysis of publication bias for the top three most reported microRNAs according to the number of studies (miR-126-3p, miR-15a-5p, miR-155-5p) and the top three most reported upregulated microRNAs and the top three most reported downregulated microRNAs (according to both the number of studies and the statistical significance) are presented in the main text. The four substudies of the most reported downregulated microRNA, miR-593, were part of the same study, which does not fit the models in Egger’s and Begg’s tests; therefore, only two of the top three most reported downregulated microRNAs were tested and a total of eight microRNAs are presented in Table 1. Typical funnel plots are presented in ESM Fig. 1, showing various levels of asymmetry across the studies and indicating some publication bias in the case of miR-126-3p, miR-320a, miR-29a-3p, miR-29c-3p and miR-30c-5p. Begg’s tests and Egger’s tests confirmed the statistical significance of the publication bias in miR-126-3p, miR-320a, miR-29a-3p, miR-29c-3p and miR-30c-5p (Table 1).

**Table 1:** Results of Begg’s and Egger’s tests

Feature	MicroRNA	No. of studies	Begg’s test		Egger’s test	
			Kendall’s tau	*p* value	*Z*	*p* value
Top three most reported	miR-126-3p	29	0.5473	<0.0001	2.7148	0.0066
	miR-15a-5p	19	–0.1071	0.5274	–0.6692	0.5034
	miR-155-5p	18	0.1518	0.4193	0.2765	0.7822
Top three most upregulated	miR-320a	14	–0.7111	0.0004	–1.0196	0.3079
	miR-29a-3p	8	–1.0000	0.0007	–2.2346	0.0254
	miR-29c-3p	8	–1.0000	0.0007	–4.2115	<0.0001
Second and third most downregulated	miR-30c-5p	6	1.0000	0.0028	1.8795	0.0602
	miR-1-3p	4	1.0000	0.0833	0.1333	0.8939

### MicroRNA pathway modelling and biomarker selection

A total of 384,579 microRNA–target interaction pairs were identified from the miRTarBase, miRecords and TransmiR databases (ESM Table 18). After alignment of microRNA names to avoid duplication, 382,633 microRNA–target interaction pairs remained. After filtering by the number of supporting articles, the top 1.4% (108,812/7,765,056) of prediction pairs from TargetScanHuman and the 1203 articles on microRNAs from Retraction Watch, 3290 robust interaction pairs were identified from 1966 articles (ESM Table 36). The 3290 interactions were further combined with KEGG pathways, producing 225 microRNA-regulated pathways (see https://osf.io/e9v7f). Extensive meta-analyses identified 138 statistically significantly dysregulated microRNAs, of which 124 microRNAs were dysregulated in a consistent direction in various meta-analyses. Pathway analysis found that the 124 dysregulated microRNAs were statistically significantly enriched in type 2 diabetes-related pathways (ESM Table 19), such as diabetic cardiomyopathy, insulin resistance, advanced glycation end products (AGE)/receptor for advanced glycation end products (RAGE) signalling-mediated diabetic complications and the type 2 diabetes pathway. The priority verification order (according to the following order of importance: [1] detectable in blood or blood fractions; [2] statistically significance in different analyses) for the 16 microRNAs enriched in the type 2 diabetes pathway and meeting the criteria for biomarker selection (i.e. statistically significant and biologically relevant) is as follows: miR-29a-3p, miR-221-3p, miR-126-3p miR-26a-5p, miR-503-5p, miR-100-5p, miR-101-3p, mIR-103a-3p, miR-122-5p, miR-199a-3p, miR-30b-5p, miR-130a-3p, miR-143-3p, miR-145-5p, miR-19a-3p and miR-311-3p (ESM Table 20).

## Discussion

This deep and comprehensive meta-analysis identified specific microRNAs as biomarkers of type 2 diabetes, yielding more significant findings over our pilot meta-analysis [8] because of the sixfold growth in the number of microRNA profiling studies since the pilot study was carried out. In particular, we conducted extensive subgroup and sensitivity analyses, publication bias analyses and analysis of the biological significance of statistically significant microRNAs. To our knowledge, no other microRNA meta-analytical studies have employed all these methods. In particular, quality assessment has seldom been conducted in previous studies.

Our meta-analytical validation showed variations and discrepancies among the microRNA studies. As an overview, a Venn diagram of the microRNA categories and systematic review flow chart are provided in ESM Fig. 2. A total of 205 DEMs were reported in at least two independent substudies, of which 60 were identified as being statistically significant by meta-analysis. Several factors may explain these meta-analysis results, including publication bias (although a comprehensive literature search was conducted, publication bias did exist), biological complexity (e.g. variations in environmental background and gene susceptibility), the use of inconsistent methods to detect microRNAs and determine differential expression, and a lack of information about expression in different tissue types under various conditions.

The subgroup analyses of both tissue type and blood fractions revealed heterogeneity in microRNA dysregulation. Subgroup analyses of tissue type found that there were no overlap microRNAs among the tissues investigated and indicated that the microRNAs in all studies may be tissue specific. Subgroup analysis of blood fractions found inconsistencies in dysregulation for 10 of 11 microRNAs, which further revealed the heterogeneity among specimens.

**Fig. 3:**
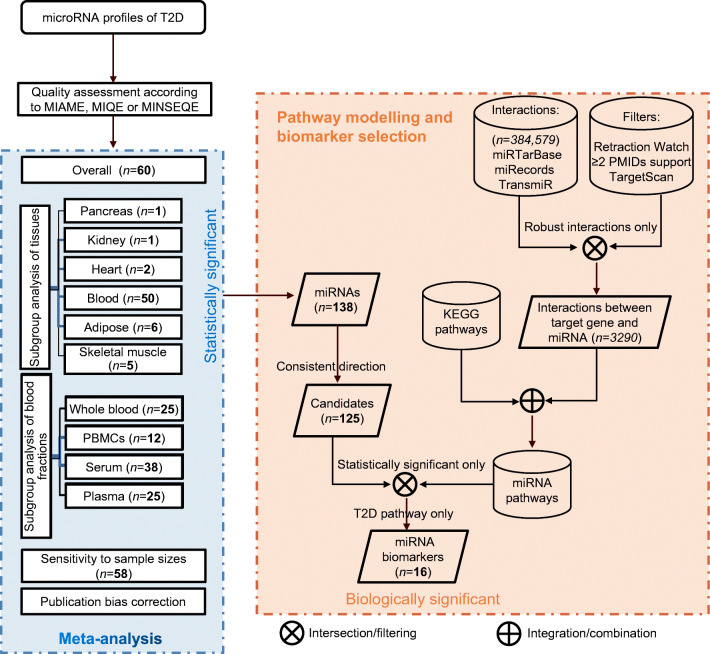
Flow diagram showing the stepwise selection of microRNA biomarkers. Statistically significant microRNAs were first identified by meta-analysis. Experimentally validated microRNA–target interactions were used to construct microRNA-regulated pathways after being filtered. Pathway analysis of dysregulated microRNAs was used to identify biologically relevant biomarkers. Numbers in bold and in italics indicate the numbers of statistically significant microRNAs and the numbers of interactions between microRNAs and targets, respectively. T2D, type 2 diabetes

**Fig. 4:**
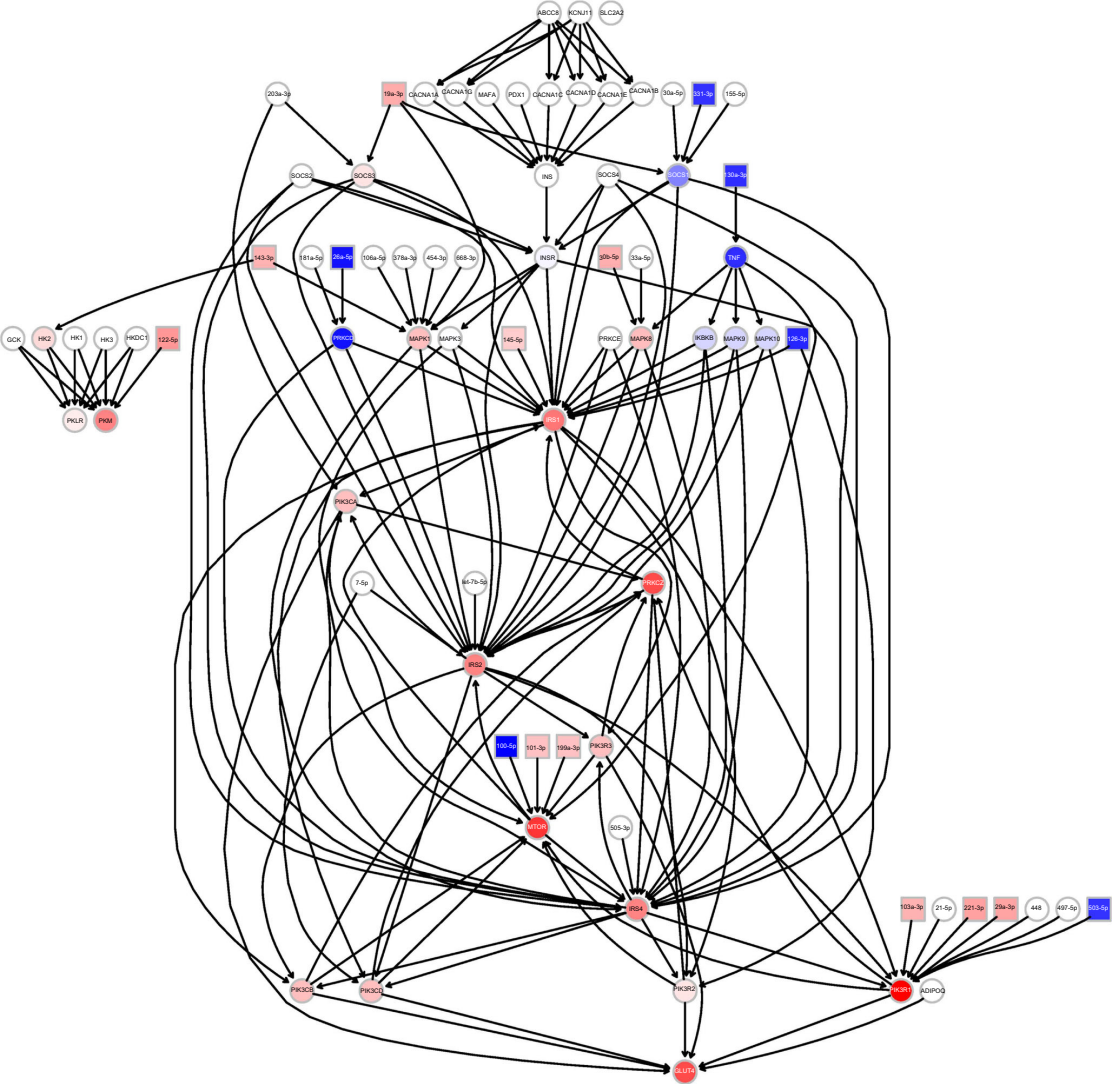
Perturbation propagation in the type 2 diabetes pathway. Coloured boxes indicate input microRNAs for which dysregulation was measured; red indicates upregulation and blue indicates downregulation. Coloured circles indicate affected genes; the darker the colour, the more affected the gene is. The prefix ‘miR-’ for the microRNA identifiers has been omitted

Our pathway analysis identified the biologically significant microRNAs from among those found to be the most statistically significant in meta-analyses. Figure 3 shows the workflow for biomarker selection. Our analysis has several important advantages over previous microRNA enrichment analyses: (1) a set of dysregulated microRNAs and their effect sizes (based on meta-analyses), as well as pathway topology, were considered by using a pathway impact analysis algorithm; and (2) the latest and most comprehensive microRNA-regulated pathways were used, which (3) were constructed from robust microRNA–target interactions, excluding data from retractions. Pathway analysis found that 16 microRNAs were enriched in the augmented KEGG type 2 diabetes pathway (Fig. 4). We recommend that miR-29a-3p is prioritised for verification, as it was statistically significant in various analyses and was one of the circulating biomarkers identified in our pilot study. According to Fig. 4, circulating miR-29a-3p, miR-221-3p, miR-103a-3p and miR-503-5p all target *PIK3R1*, which encodes regulatory subunit 1 of phosphoinositide-3-kinase [35–38]. Phosphoinositide-3-kinase (PI3K) plays an important role in the metabolic actions of insulin and a mutation in the *PIK3R1* gene has been associated with insulin resistance [39]. PI3K has a large impact on GLUT4, the insulin-regulated facilitative glucose transporter located downstream in the type 2 diabetes pathway (Fig. 4). This is partly why the dysregulated input microRNAs (especially the four mentioned above) are also enriched in the PI3K-Akt signalling pathway and in insulin resistance (ESM Table 19). miR-199a-3p was identified as a potential biomarker in both this study and our previous meta-analysis [8]; this microRNA regulates *MTOR*, which encodes the mechanistic target of rapamycin kinase, along with miR-100-5p and miR-101-3p. These three microRNAs mediate protein synthesis, cell growth and proliferation and the cell cycle through *MTOR* [40–42]. *IRS1*, which encodes insulin receptor substrate 1, is one of the key elements in Fig. 4, although it is affected only by dysregulated miR-145-5p and miR-126-3p [43, 44], *IRS1* is impacted greatly by upstream elements. In addition, miR-126-3p has been reported to inhibit *IRS1* expression, resulting in downregulation of PI3K in diabetic retinopathy [45], and miR-145-5p regulates glucose uptake and insulin signalling by targeting *IRS1* [46], both of which are consistent with Fig. 4. In addition to these published studies, it is encouraging that new clinical trials investigating the effects of microRNAs on type 2 diabetes and its complications (e.g. NCT02459106, NCT02316522, NCT04889053) and the associations between drug treatments and microRNAs in type 2 diabetes (e.g. NCT01334684, NCT03377335, NCT03472846) have been registered at https://clinicaltrials.gov/.

This deep meta-analysis identified, verified and corroborated the available evidence on both the biological and the statistical significance of microRNAs in type 2 diabetes. Significant findings compared with those of our pilot study conducted in 2014 include the identification of 13 new microRNAs with the potential to serve as biomarkers in type 2 diabetes. Seven microRNAs identified in our pilot study were abandoned because of inconsistencies with later studies, little biological significance and changes to biomarker selection criteria. For instance, miR-107 was found to be upregulated in two earlier studies included in our pilot study, but two later studies included in the present study showed that miR-107 is downregulated. After meta-analysis, miR-107 was no longer significant. ESM Table 21 summarises the contradictory findings on human microRNAs between our pilot study and the present meta-analysis.

Recent studies indicate that seminal findings from academic laboratories can be reproduced only 11–50% of the time [47, 48]. A survey on biomedical science also identified the issue of non-reproducibility of data [49]. This issue is exacerbated by a lack of reporting of experimental details, which hinders quality assessment and study reproducibility. The MIAME guideline (2001) and MIQE guideline (2009) were published over 20 and 10 years ago, respectively. The aim of these guidelines was to establish a standard for recording and reporting biomedical experiments, which in turn would facilitate the establishment of databases and public repositories, enable the development of data analysis tools and encourage the exchange of data. Of the 156 included studies published between 2009 and 2020, none referred to the MIAME guideline (2001) and MIQE guideline (2009), even though 155 were published after 2009. The quality assessment found that only one study met all the basic criteria of the MIAME guideline and MIQE guideline, while 85% of the studies did not report raw data on hybridisation and more than 80% did not provide sufficient information on experimental design. This indicates that it would not be possible to rely on any individual study for biomarker development. In addition, 1203 microRNA studies had been retracted or had concerns over data integrity according to our latest search (September 2021) of the Retraction Watch database. Although we did not include such studies, some microRNA-related databases did include them, such as the three microRNA–target interaction databases (miRTarBase, miRecords and TransmiR) that we used and the human microRNA disease database (HMDD) [50]. Our deep meta-analysis fills these technical gaps, enabling microRNAs to be selected with very high levels of statistical and biological confidence.

This study has several potential limitations. Differences in the characteristics of participants, study design, sample collection and measurement, microRNA detection methods and their performance, differential expression analysis methods and DEM selection criteria as well as normalisation strategies may lead to bias and errors in using microRNAs as molecular biomarkers. The present study identified potential microRNA biomarkers without normalising all the factors mentioned above. Future microRNA expression profiling studies should report data in more detail to enable evaluation of the clinical utility of (new) microRNA candidates. The discovery and selection of microRNA biomarkers was limited by the study designs of the included studies and the study design of this study, which were influenced by researchers’ experience, scientific background, personal preferences and interests, etc. In the future, systematic generation and evaluation of evidence will improve the scientific value of biomarker studies.

### Conclusion

This deep meta-analytical corroboration of type 2 diabetes microRNA expression profiling studies and microRNA-regulated pathways using stringent quality criteria identified 16 microRNA biomarkers for type 2 diabetes that are both statistically significant and biologically relevant. These should be prioritised in the following order for verification: miR-29a-3p, miR-221-3p, miR-126-3p, miR-26a-5p, miR-503-5p, miR-100-5p, miR-101-3p, mIR-103a-3p, miR-122-5p, miR-199a-3p, miR-30b-5p, miR-130a-3p, miR-143-3p, miR-145-5p, miR-19a-3p and miR-311-3p.

## Supplementary Information

ESM (PDF 862 kb)

ESM Table 36 (CSV 729 kb)

## Data Availability

All data generated or analysed during this study are included in this published article (and its supplementary information files and at https://osf.io/e9v7f).

## Abbreviations

DEM: Differentially expressed microRNA
EB: Empirical Bayes
KEGG: Kyoto Encyclopedia of Genes and Genomes
MIAME: Minimum Information About a Microarray Experiment
MINSEQE: Minimum Information for Publication of Quantitative Real-time PCR Experiments
MIQE: Minimum Information for Publication of Quantitative Real-time PCR Experiments
mirAP: MicroRNA-augmented pathways
PBMC: Peripheral blood mononuclear cell
PI3K: Phosphoinositide-3-kinase
REML: Restricted maximum likelihood
