# Supplementary material for: MicroRNA biomarkers of type 2 diabetes: evidence synthesis from meta-analyses and pathway modelling
Source: Diabetologia. 2022 Oct 21;66(2):288–99. doi: 10.1007/s00125-022-05809-z (PMC9807484; doi:10.1007/s00125-022-05809-z)
Supplement: Supplementary file 1 — (PDF 862 kb) [file 125_2022_5809_MOESM1_ESM.pdf]

Supplemental Data include two figures, thirty-six tables and references of included studies.

ESM Table 1. Characteristics of human miRNA expression profiling studies [T2D vs nondiabetic controls (CTR)]

ESM Table 2. Quality assessment according to the MIAME and MIQE guideline

ESM Table 3. Statistically significant dysregulation of miRNAs in type 2 diabetes (n= 60)

ESM Table 4. Statistically significant dysregulation of miRNAs in pancreas (n=1)

ESM Table 5. Statistically significant dysregulation of miRNAs in heart (n=2)

ESM Table 6. Statistically significant dysregulation of miRNAs in kidney (n=1)

ESM Table 7. Statistically significant dysregulation of miRNAs in adipose (n=6)

ESM Table 8. Statistically significant dysregulation of miRNAs in muscle (n=5)

ESM Table 9. Statistically significant dysregulation of miRNAs in blood (n=50)

ESM Table 10. Statistically significant dysregulation of miRNAs in PBMCs (n=12)

ESM Table 11 Statistically significant dysregulation of miRNAs in whole blood (n=25)

ESM Table 12. Statistically significant dysregulation of miRNAs in serum (n=38)

ESM Table 13. Statistically significant dysregulation of miRNAs in plasma (n=25)

ESM Table 14. Statistically significant dysregulation of miRNAs in blood fractions (n=87)

ESM Table 15. Statistically significant dysregulation of miRNAs detected by PCR-based methods (n = 61)

ESM Table 16. Statistically significant dysregulation of miRNAs detected by RNA-Seq (n = 11)

ESM Table 17. Statistically significant dysregulation of miRNAs in sensitivity analysis based on sample sizes (n= 90)

ESM Table 18. The characteristics of databases for human microRNA-target gene interactions

ESM Table 19. Pathway analysis on dysregulated microRNAs from meta-analyses with newly built microRNA regulated pathways

ESM Table 20. Potential microRNA biomarkers enriched in T2D pathway and their priority verification order

ESM Table 21. Contradictory findings on human microRNAs between previous and present

meta-analysis due to newly included studies
ESM Table 22. Statistically significant dysregulation of miRNAs in humans by empirical Bayes estimation (EBM) (n = 60)
ESM Table 23. Statistically significant dysregulation of miRNAs in human pancreas by EBM (n=1)
ESM Table 24. Statistically significant dysregulation of miRNAs in human heart by EBM (n=2)
ESM Table 25. Statistically significant dysregulation of miRNAs in kidney by EBM (n=1)
ESM Table 26. Statistically significant dysregulation of miRNAs in adipose by EBM (n=6)
ESM Table 27. Statistically significant dysregulation of miRNAs in human muscle by EBM (n=5)
ESM Table 28. Statistically significant dysregulation of miRNAs in human blood by EBM (n=50)
ESM Table 29. Sensitivity analysis based on human sample size by EBM (n=90)
ESM Table 30. Statistically significant dysregulation of miRNAs in PBMCs by empirical Bayes estimation (n = 12)
ESM Table 31. Statistically significant dysregulation of miRNAs in Whole blood by EBM (n = 25)
ESM Table 32. Statistically significant dysregulation of miRNAs in serum by EBM (n = 38)
ESM Table 33. Statistically significant dysregulation of miRNAs in plasma by EBM (n = 25)
ESM Table 34. Statistically significant dysregulation of miRNAs detected by PCR-based methods and EBM (n = 61)
ESM Table 35. Statistically significant dysregulation of miRNAs detected by RNA-Seq and EBM (n = 11)
ESM Table 36. Robust microRNA-gene interaction pairs supported by 1966 articles (n=3290)
ESM Fig. 1. Funnel plots of publication bias on different microRNAs
ESM Fig. 2. The Venn diagram of microRNA categories and flow chart of systematic review
Appendix
References of included studies

ESM Table 1. Characteristics of human miRNA expression profiling studies [T2D vs nondiabetic controls (CTR)]

Study	T2D								Differentially expressed miRNAs		
	Country	Period	Tissue	Clinical status	No. of samples (T2D/CTR)	Age (years)	BMI (kg/m^2)	HbA1c (%)	Platform	Cut-off criteria	Total (Up/Down)
Al-Hayali MA 2019 [1]	Turkey	NR	Serum	Diagnosed	T2D: 45<br>T2D+CAD: 45<br>T2D+HF: 45<br>CTR: 45	61.50±5.08<br>61.61±6.02<br>62.07±5.26<br>60.23±6.27	29.86±3.18<br>29.62±2.63<br>29.21±1.66<br>23.33±1.39	10.23±9.67<br>7.46±1.42<br>5.63±0.73<br>4.74±0.47	StepOnePlus RT-PCR system (Applied Biosystems) with MPLIFYME SYBR UniversalMix (BLIRT)	*p<0.05*	2 (1/1)
Alicka M 2019 [2]	Poland	NR	ASC (adipose); extracellular vesicles of ASC	Diagnosed	12 (6/6)<br>12 (6/6)	36-49<br>38-56	41.50±5.50<br>15.45±2.21	NR	CFX Connect™ RT- PCR Detection System (Bio-Rad)	*p<0.05*	6 (5/1)<br>6 (6/0)
Alipoor B 2018 [3]	Iran	NR	PBMCs<br>Plasma	Diagnosed	60 (30/30)	57<br>50.5	27.13±4.15<br>27.22±3.26	7.4<br>5.1	RT-PCR with SYBR Premix Ex TaqTM II (Takara)	*p*<0.05	1 (0/1)<br>1 (0/1)
Al-Kafaji G 2015 [4]	Kingdom of Bahrain	NR	Peripheral whole blood	Diagnosed	48 (24/24)	52±6.0<br>49±9.1	25.3±1.8<br>24.2±1.0	7.5±0.8<br>4.8±0.6	TaqMan miRNA Assays and RT-PCR system (Applied Biosystems)	*p*<0.05	1 (0/1)
Al-Kafaji G 2016 [5]	Kingdom of Bahrain	NR	Peripheral whole blood	Diagnosed	T2D: 52,<br>T2D+DN: 50,<br>CTR: 50	62.0±10.5<br>64.6±6.3<br>56±5.2	25.2±4.5<br>26.3±5.2<br>24.2±4.1	8.9±2.7<br>9.3±1.8<br>4.9±0.7	TaqMan miRNA Assays and 7900HT PCR system (Thermo Fisher Scientific)	*p*<0.05	1 (0/1)
Al-Kafaji G 2017 [6]	Kingdom of Bahrain	NR	Peripheral whole blood	Diagnosed	T2D: 45<br>T2D+CAD: 45<br>CTR: 45	61±12<br>64±11.7<br>53±8.6	25.4±4.8<br>26.1±4.3<br>24±4.5	7.4±8.3<br>9.6±3.2<br>3.64±1.1	TaqMan miRNA Assays (Applied BioSystems)	*p*<0.05<br>Ct <35	1 (0/1)
Avgeris M 2020 [7]	Greece	NR	Peripheral whole blood	Diagnosed	Screening: 12 (6/6)<br>Validation: 77 (40/37)	NR<br>35-75<br>19-69	NR<br>21.5-46.5<br>21.3-36.3	NR<br>5.2-12.1<br>5.0-6.1	miScript PCR Array and SYBR Green kit (Qiagen) in 7500 RT-PCR system (Applied Biosystems)	*p*<0.05	7 (0/7)<br>3 (0/3)

Bai L 2019 [8]	NR	NR	Blood	Diagnosed	40 (20/20)	60±5.3<br>61.2±5.5	34±6.5<br>32.5±5.5	7.8±7.1<br>5.0±4.1	RT-PCR	$p<0.05$	1 (1/0)
Bai X 2016 [9]	China	2012 to 2014	Plasma	Diagnosed	T2D+DN/CTR: 47 (27/20)	NR	NR	NR	miR-130b miScript Primer Assays (Qiagen)	$p<0.05$	1 (0/1)
Balasubramanyam M 2011 [10]	Chennai India	NR	PBMCs	Diagnosed	40 (20/20)	43.7±5.1<br>42.0±4.7	26.4±3.7<br>25.8±4.0	7.9±1.8<br>5.5±0.2	miRCURY LNA miRNA PCR system (Exiqon)	$p<0.05$	1 (0/1)
Baldeon RL 2015 [11]	Ecuador	2009 to 2012	PBMCs	Diagnosed	82 (48/34)	NR	NR	NR	TaqMan miRNA Assays and 7900 HT RT-PCR system (Applied Biosystems)	$p\leq0.05$	2 (2/0)
Baltaci OF 2018 [12]	Turkey	NR	Blood	Diagnosed	8 (5/3)	NR	NR	NR	TaqMan probes (Thermo Fisher Scientific) with qRT-PCR	$p<0.05$	1 (1/0)
Bao L 2015 [13]	China	2011 and 2013	Plasma	Diagnosed	T2D: 34<br>Pre-diabetes: 27<br>CTR: 30	NR	NR	NR	SYBR Green Master Mix; TaqMan Universal Master Mix II.	$p<0.05$	1 (0/1)
Blum A 2019 [14]	Israel	NR	Serum	Diagnosed	37 (15/22)	65±9<br>39±10	NR	NR	TaqMan Advanced miRNA assays (Applied Biosystems)	$p<0.05$	1 (0/1)
Bork-Jensen J 2015 [15]	Denmark	NR	Skeletal muscle	Diagnosed	22 (11/11)	68±8<br>68±8	32.5±6.7<br>30.4±6.5	7.7±1.5<br>5.9±0.5	miRCURY LNA array (Exiqon,)	pFDR<0.1	20 (0/20)
Brovkina O 2019 [16]	Russia	NR	Adipose	Diagnosed	70 (35/35)	48.5<br>44.5	41.80<br>43.02	8.00<br>5.40	TaqMan Advanced miRNA Assays (Thermo Fisher Scientific)	$p<0.05$	7 (5/2)
Candia PD 2017 [17]	Italy	NR	Plasma	Newly diagnosed	18 (9/9)	60.2±8<br>57.9±8.9	29.6±7.8<br>23.7±3.3	6.40±0.58<br>5.52±0.2	miRCURY RNA PCR panels (Exiqon, Denmark,)	$p<0.05$	3 (1/2)
Cheng S 2018 [18]	China	Aug 2016 to May 2017	$CD34^+$ cells from blood	Diagnosed	33 (18/15)	NR	NR	NR	SYBR PrimeScript miRNA RT-PCR (Takara)	$p<0.05$	1 (0/1)

Cheng Y 2020 [19]	Shanghai China	2015 to 2017	Kidney	Diagnosed	17 (10/7)	54.0±3.6<br>48.0±6.7	NR	NR	TaqMan miRNA Assay Kit (Life Technologies)	*p*<0.05	1 (0/1)
Conserva F 2019 [20]	Italy	NR	Kidney	Diagnosed	Discovery: (CTR: 4,<br>T2D+DN: 6,<br>T2D+MN: 6<br>Validation:17 (12/5)	38±14<br>59±16<br>63±16<br>NR	26±4<br>33±4<br>26±4<br>NR	NR	Agilent Human miRNA Microarray; miScript SYBR Green PCR Kit	FC≥1.5 & *pFDR*≤0.05	65 (29/36)<br><br>1 (0/1)
Corral-Fernandez NE 2013 [21]	Mexico	NR	PBMCs	Diagnosed	40 (20/20)	40-60<br>18-28	31.9±7.4<br>23.1±2.5	7.9±1.7<br>4.8±0.7	TaqMan miRNA Assays (Applied Biosystems)	*p*<0.05	2 (0/2)
Cui X 2017 [22]	Nanjing China	NR	Serum	Newly diagnosed	183 (101/82)	57.5±12.2<br>49.3±7.73	26.8±4.19<br>24.3±3.22	NR	TaqMan and ViiA7 qRT-PCR system (Applied Biosystems)	FC≥2 & *p*≤0.05	3 (3/0)
Dahlmans D 2017 [23]	Netherlands	NR	Skeletal muscle	Diagnosed	n=12 for each group (T2D, obese, lean and athlete)	58.64±1.22<br>57±2.29<br>21.91±0.72<br>25.08±1.25	32.37±1.11<br>30.83±1.14<br>21.84±0.57<br>20.98±0.44	NR	384-well Pick-&-Mix miRNA PCR Panel<br>plates (Exiqon)	*p*<0.05	4 (4/0)
Dai X 2016 [24]	Shanghai China	Apr 2009 to Oct 2012	Serum	Newly diagnosed	T2D+PC/CTR: 80/80	60.1±10.7<br>59.5±13.5	NR	NR	TaqMan low-density array; ABI7500 RT-PCR system (Applied Biosystems)	*p*<0.05	6 (6/0)
Dangwal S 2015 [25]	Germany	NR	Plasma	Diagnosed	43 (23/20)	61.0±2.1<br>NR	29.1±0.9<br>NR	8.6±0.4<br>NR	Affymetrix miRNA array; TaqMan assays.	*p*≤0.05	2 (0/2)
Deng X 2017 [26]	China	Sep 2015 to Jan 2016	Blood	Diagnosed	T2D+CHD: 28<br>CHD: 35<br>CTR: 31	65.1±7.2<br>61.3±6.6<br>60.3±7.3	NR	NR	qRT-PCR with ABI7500 system (Applied Biosystems)	*p*<0.05	1 (0/1)<br>1 (0/1)
El Samaloty NM 2019 [27]	Egypt	NR	Serum	Diagnosed	T2D: 21,<br>T2D+HCV: 19,<br>HCV: 21,<br>CTR: 19	44.2±1.6<br>42±1.5<br>41.1±1.4<br>39.4±1.2	24.4±0.3<br>24.2±0.4<br>24±0.3<br>23.5±0.3	7.6±0.1<br>6.5±0.14<br>5.2±0.15<br>5.1±0.19	miScript SYBR Green kit (Qiagen) with StepOne Plus RT-PCR system (Applied Biosystem)	*p*<0.05	2 (1/1)

Elemeery MN 2019 [28]	Egypt	NR	Serum	Diagnosed	T2D: 200<br>T2D+TCC: 200<br>CTR: 225	53.68±9.48<br>56.23±8.14<br>49.92±7.14	NR	NR	TaqMan miRNA assay (Applied Biosystems)	$p<0.05$	12 (5/7)
Fejes Z 2017 [29]	Debrecen, Hungary	NR	Platelets;<br>Plasma	Diagnosed	T2D: 28<br>Obese CTR: 19<br>CTR: 23	50-59<br>48-57<br>34-60	32.9<br>35.8<br>24	7.5<br>5.5<br>NR	TaqMan miRNA assay kits (Applied Biosystems)	$p\leq0.05$	3 (0/3)<br>4 (0/4)
Fomison-Nurse I 2018 [30]	New Zealand	NR	Heart<br>Plasma<br>Plasma	Diagnosed	T2D+IHD/IHD: 10/11 for heart and plasma; T2D: 11, 14, or 14 for plasma at different duration<br>CTR: 32	67.07<br>64.56<br>61.45<br>67.36<br>70.86<br>69.25	31.06<br>30.27<br>27.92<br>27.47<br>25.84<br>26.42	7.42<br>5.74<br>7.07<br>7.54<br>7.91<br>5.79	TaqMan hybridization probe	$p<0.05$	1 (1/0)<br>1 (1/0)<br><br>1 (1/0)
Fulzele S 2015 [31]	USA	NR	Vitreous	Diagnosed	12 (8/4)	NR	NR	NR	miScript reagents (Qiagen) & SYBR Green I	$p<0.05$	1 (0/1)
Gallagher IJ 2010 [32]	Scotland, UK	NR	Skeletal muscle tissue	Diagnosed	92 (45/47)	54.8±10.2<br>51.3±10.7	31.4±6.2<br>31.1±7.2	7.4±1.8<br>5.5±0.2	miRCURY LNA miRNA array (Exiqon) and TaqMan Assays (Applied Biosystems)	SAM; FDR<0.1 (FC>1.3)	62 (29/33)
Giannella A 2017 [33]	Padua Italy	NR	Plasma	Diagnosed	121 (68/53)	60±1<br>57±1	30±1.6<br>25±0.4	NR	TaqMan microRNA kit (Applied Biosystems) & PCR with CFX96 (Biorad)	$p<0.05$	1 (0/1)
Guo J 2017 [34]	Zhengzhou China	Oct 2014 to Jul 2015	Plasma	Diagnosed	68 (33/35)	55.9±2.96<br>45.33±4.12	25.57±1.64<br>22.26±0.44	9.10±0.44<br>5.25±0.16	miRCURY LNA miRNA array (Exiqon)	FC>2	1 (1/0)
Guglielmi V 2017 [35]	NR	NR	Subcutaneous adipose tissue	4 diagnosed; 5 newly diagnosed	16 (9/7)	43.5±8<br>39.2±10.9	47.6±8.1<br>45.9±9.9	7.57±1.64<br>5.41±0.90	TaqMan miRNA array with ABI 7000 PRISM (Applied Biosystems)	$p<0.05$	1 (1/0)
Han YL 2016 [36]	Shandong,	Jan 2012 to	Blood	Diagnosed	T2D+BC/BC: 72/144	52.72±6.23	24.68±4.74	10.57±2.11	ABI 7500 quantitative PCR	$p<0.05$	2 (1/1)

	China	Jan 2015				50.08±4.76	23.21±3.25	5.64±1.57	System (Life Technologies)		
He X 2016 [37]	China	NR	Plasma	12 diagnosed;<br>20 Newly diagnosed	n=12 for each group (CTR, ASO, T2D+ASO);<br>T2D: 20	NR	NR	NR	miRCURY LNA array (v.18.0); miScript SYBR Green PCR kit in ABI StepOne Plus PCR system	*p*<0.05	4 (3/1)
He X 2018 [38]	China	NR	Vascular tissues	Diagnosed	T2D+ASO/CTR: 26 (16/10)	NR	NR	NR	miScript SYBR Green PCR kit in ABI StepOne Plus PCR system	*p*<0.05	1 (1/0)
Henriksen TI 2017 [39]	Denmark	NR	Muscle stem cells	Diagnosed	16 (8/8)	51-65<br>50-67	26.2±2.8<br>25.4±1.8	NR	miRCURY LNA miRNA array (Exiqon)	*p*<0.05	5 (0/5)
Hou XY 2016 [40]	China	2010 to 2015	Plasma	Diagnosed	T2D+DN/CTR: 60 (30/30)	NR	NR	NR	TaqMan miRNA array; ABI7500 RT-PCR system (Applied Biosystems)	*p*<0.05	1 (1/0)
Hou X 2016 [41]	China	NR	Islets	Diagnosed	68 (34/34)	54±8<br>52±7	34±5<br>31±4	6.08±0.46<br>5.26±0.37	miScript SYBR Green PCR Kit (Qiagen)	*p*<0.05	1 (1/0)
Jansen F 2016 [42]	Bonn, Germany	Aug 2012 to Jul 2013	Plasma	Diagnosed	135 (55/80)	67.6±10.6<br>65.5±11.1	30.5±5.4<br>26.9±4.5	7.2±1.2<br>5.9±0.4	TaqMan miRNA Assays and 7500 HT RT-PCR system (Applied Biosystems)	*p*<0.05	2 (0/2)
Jiang Q 2017 [43]	Changchun, China	May 2013 to Mar 2015	Plasma	Diagnosed	T2D: 65<br>T2D+BDR: 73<br>T2D+PDR: 51<br>CTR: 115	47.76±8.05<br>48.83±7.13<br>50.75±10.2<br>48.53±7.26	22.67±1.98<br>23.06±1.85<br>23.13±2.02<br>22.82±2.33	6.25±0.58<br>7.02±0.66<br>8.16±0.79<br>4.30±0.42	qRT-PCR (Qiagen Company)	*p*<0.05	1 (1/0)
Jiang X 2015 [44]	Nanjing, China	Jul 2012 to Dec 2013.	Serum	Diagnosed	T2D+ED/CTR: 40/40	47.37±5.81<br>46.9±5.69	26.2±3.47<br>23.79±3.47	8.22±1.08<br>4.97±0.83	PCR 7500 (Applied Biosystems)	*p*<0.05	3 (3/0)
Jiao Y 2015 [45]	Urumqi China	NR	Peripheral blood<br>Adipose tissues	Diagnosed	36 (12/24)	56±10<br>49±13	30.9±5.8<br>26.3±3.6	NR	miRCURY array and chip (Exiqon); MiScript SYBR	*p*<0.05	3 (0/3)

					24 (12/12)	57±11<br>53±14	27.9±4.2<br>25.7±4.2		Green PCR (Qiagen)		1 (0/1)
Jones A 2017 [46]	New Zealand	NR	Plasma	Diagnosed	27 (15/12)	45±8<br>44±9	44.2±4.2<br>22.9±2.6	7.5±1.7<br>NR	miRCURY LNA miRNA PCR ExiLENT SYBR green master mix	*Adjusted*<br>*p*<0.05	42 (13/29)
Kamalden TA 2017 [47]	NR	NR	Plasma	Diagnosed	T2D: 41,<br>T2D+BDR: 22,<br>CTR: 19	62.1±13.2<br>61.4±8.7<br>64.5±8.3	NR	7.2±1.2<br>9.0±2.6<br>NR	miScript SYBR Green PCR kit (Qiagen)	*p*<0.05	1 (1/0)
Karolina DS 2011 [48]	Singapore	Jul 2008 to Apr 2009	Whole blood	Diagnosed	15 (8/7)	46.7±3.4<br>46.3±7.5	24.5±1.1<br>22.4±2.3	NR	miRCURY LNA miRNA array (Exiqon) and TaqMan Assays (Applied Biosystems)	*p*<0.05	82 (48/34)
Karolina DS 2012 [49]	Singapore	Jul 2008 to Jul 2012	Whole blood	Diagnosed	96 (50/46)	42.02<br>43.22	26.03<br>23.29	NR	miRCURY LNA miRNA array (Exiqon) and TaqMan Assays (Applied Biosystems)	*p*<0.05	11 (5/6)
Katayama M 2019 [50]	Sweden	NR	Exosomal from serum	Diagnosed	8 (4/4)<br>41 (21/20)	NR<br>61±1<br>60±1	NR<br>29.5±0.6<br>28.8±0.4	NR<br>6.34±0.3<br>4.6±0.1	StepOne Plus PCR (Applied Biosystems); miRCURY LNA miRNA PCR System (Exiqon)	*p*<0.05	6 (3/3)<br>2 (2/0)
Khan R 2020 [51]	India	NR	Serum	Diagnosed	Screening:10 (5/5)<br>Validation: same sample 10 (5/5)	42±7.16<br>34±6.26	23.3 ± 0.89<br>21.4 ± 0.89	NR	Illumina Next Seq500; miscript SYBR green PCR kit (GmBH)	*p*<0.05;<br>FC ≥ ± 2.	28 (9/19)<br>7 (3/4)
Kloting N 2009 [52]	Germany	6 months	Subcutaneous (SC)/omental adipose	Newly diagnosed	15 (6/9)	67±2.8<br>66±3.3	31.2±2.1<br>28.6±2.0	6.7±0.8<br>5.4±0.4	TaqMan miRNA Assays (Applied Biosystems)	*p*<0.05	11 (3/8)
Kokkinopoulou I 2019 [53]	NR	NR	Peripheral blood	Diagnosed	77 (40/37)	35-75<br>19-69	21.5-46.5<br>21.3-36.3	5.2-12.1<br>5.0-6.1	7500 RT-PCR System (Applied Biosystems)	*p*<0.05	6 (0/6)
Kong L 2010 [54]	Shandong,	Feb 2009 to	Serum	Newly	44 (18/26)	47.33±2.62	26.26±0.79	NR	TaqMan miRNA Assays (ABI	C_t=40;	1 (1/0)

	China	Jun 2009		diagnosed		43.81±2.22	25.57±3.58		company)	$p<0.01$	
Kong L 2011 [55]	Shandong,<br>China	NR	Serum	Newly<br>diagnosed	37 (18/19)	47.33±2.62<br>41.00±2.62	26.26±0.79<br>26.63±0.80	NR	TaqMan miRNA Assays<br>(Applied BioSystems)	$p<0.05$	7 (7/0)
Krause C 2020 [56]	Germany	NR	Liver	Diagnosed	78 (29/49)	49.84±11.9<br>39.6±10.87	51.35±9.27<br>53.93±11.39	8.15±1.68<br>5.48±1.02	TaqMan miRNAAssays<br>(Applied BioSystems)	$p<0.05$	1 (1/0)
Kuschnerus K 2019<br>[57]	Switzerland	NR	M2-like<br>macrophages；<br>Aortic wall	Diagnosed	T2D+CAD/CAD: 7/7<br>T2D+CAD/CAD: 7/5	NR	NR	NR	miRCURY LNA miRNA<br>RT-PCR Kit and In Situ<br>Hybridization kit (Exiqon)	$p<0.05$	1 (1/0)<br><br>1 (1/0)
Lareyre F 2019<br>[58]	France	NR	PBMCs<br>PBMCs<br>Serum<br>Aneurysmal<br>tissue (ANT)	Diagnosed	Screening: 6 (3/3)<br>Validation: 20<br>(10/10) for PBMCs<br>and serum; 6 (3/3)<br>for ANT	NR<br>63.8-75.3<br>66-74.5<br>NR	NR<br>24.4-28.8<br>22.2-27.6<br>NR	NR	miRCURY LNA Universal RT<br>miRNA PCR (Exiqon-<br>Qiagen)	$p<0.05$	4 (3/1)<br>3 (3/0)<br>1 (1/0)<br>2 (2/0)
Latouche C 2016<br>[59]	Australia	NR	Skeletal muscle	Diagnosed	11 (6/5)	53±2<br>48±3	33±2<br>25±1	NR	Agilent 8x60K Human<br>miRNA microarray	$p<0.05$	23 (13/10)
Latreille M 2014<br>[60]	Sweden	NR	Islets	Diagnosed	19 (9/10)	54.2±8.3<br>56.9±7.9	28.4±4.3<br>26.7±3.3	6.6±0.5<br>5.5±0.2	TaqMan miRNA Assays<br>(Applied Biosystems)	$p<0.05$	1 (0/1)
Li H 2019 [61]	China	Jan 2012 to<br>Oct 2014	Heart<br><br>Plasma	Diagnosed	16 (8/8)<br><br>30 (15/15)	52±4.3<br>46±3.2<br>60±4.0<br>59±1.8	NR	NR	miRCURY LNA<br>miRNA Arrays (Exiqon)	$p<0.05$	1 (1/0)<br><br>1 (1/0)
Li MY 2016 [62]	Shandong<br>China	NR	Serum	Newly<br>diagnosed	T2D: 30<br>T2D+BC: 30<br>BC-CTR: 30<br>CTR: 20	60.79±11.11<br>61.13±10.58<br>61.89±9.33<br>59.78±11.23	28.88±1.18<br>27.24±1.37<br>29.24±1.32<br>20.12±1.69	7.60±0.33<br>7.57±0.36<br>5.30±0.33<br>4.56±0.45	RT-PCR with SYBR ExTaq<br>Mix	$p<0.05$	2 (2/0)
Li YB 2017 [63]	Hubei	May 2014 to	Plasma	Diagnosed	20 (10/10)	NR	NR	NR	RT-PCR Master Mix	$p<0.05$	1 (1/0)

	China	Mar 2015<br>Jul 2011 to<br>Oct 2015	Lower limb skin	Diagnosed	60 (30/30)				SYBR-Green PCR (Toyobo<br>Co., Ltd., Osaka, Japan)		1 (1/0)
Lian W 2018 [64]	Shanghai,<br>China	NR	EPCs from blood	Diagnosed	50 (25/25)	58.2±8.3<br>60.1±8.7	23.23±4.90<br>13.35±2.90	NR	TaqMan qPCR	$p<0.05$	1 (1/0)
Liang L 2016 [65]	USA	NR	Fibroblasts from<br>foot skin<br>specimens	Diagnosed	T2D+DFU: 7<br>CTR: 8	NR	NR	NR	nCounter microRNA<br>expression (NanoString);<br>Quantification System (Quanta<br>BioSciences)	FC≥2.0;<br>$p<0.05$	3 (3/0)
Liang YZ 2018<br>[66]	Beijing<br>China	First stage:<br>2015;<br>validation<br>stage: 2017	Plasma	Newly<br>diagnosed	First 20 (10/10);<br><br>Validation T2D: 112<br>pre-diabetic: 72<br>CTR: 94	49.10±3.84<br>49.00±3.97<br>54.75±7.53<br>52.53±7.51<br>52.84±8.85	26.84±2.85<br>22.74±1.91<br>27.11±3.17<br>26.26±2.97<br>23.86±3.27	8.66±0.87<br>4.97±0.24<br>7.58±1.54<br>5.74±0.60<br>5.16±0.39	Agilent miRNA microarrays;<br>miScript RT Kit (Qiagen) with<br>GeneAmp PCR System 9700<br>(Applied Biosystems)	FC≥2.0,<br>$p<0.05$	51 (33/18)<br><br>4 (3/1)<br>2 (2/0)
Liang Z 2018 [67]	Shenzhen,<br>China	NR	Serum	Diagnosed	17 (11/6)	NR	NR	NR	SYBR-Green PCR Master<br>Mix (Applied Biosystems)	FC≥2.0,<br>FDR<0.05	4 (4/0)
Liu H 2016 [68]	Shandong<br>China	Jan2011 to<br>Dec 2014	Epithelial BC<br>tissues	Diagnosed	T2D+BC/BC: 257<br>(117/140)	53.57±6.08<br>54.36±5.52	22.83±2.78<br>24.05±3.32	NR	ABI PRISM 7000 detection<br>system (Applied Biosystems)	$p<0.05$	1 (0/1)
Liu Y 2017 [69]	China	Aug 2014 to<br>Feb 2017	Serum	Diagnosed	42 (21/21)	46.9±2.0<br>37.4±3.3	NR	NR	Real-time PCR with AB7300<br>(Stratagene, Valencia, CA)	$p<0.05$	1 (0/1)
Locke JM 2014<br>[70]	USA and UK	NR	Pancreatic islets	Diagnosed	20 (11/9)<br>20 (10/10)	53±8/47±9<br>55±9/51±6	36±14/30±6<br>32±4/29±5	NR	TaqMan miRNA Assays<br>(Life Technologies)	$p<0.05$	2 (2/0)
Long Y 2017 [71]	Hunan,<br>China	Mar 2013 to<br>Sep 2014	PBMCs	Diagnosed	34 (16/18)	57±9<br>53±11	NR	NR	TaqMan miRNA Assays<br>(Applied BioSystems)	$p<0.05$	1 (0/1)
Lu H 2010 [72]	UK	NR	Left ventricular<br>biopsies	Diagnosed	12 (6/6)	59±7<br>58±14	27.7±4.3<br>27.0±2.5	6.6±1.0<br>5.5±0.2	TaqMan miRNA Assay<br>(Applied Biosystems)	FC<-2<br>or >2,	18 (10/8)

										$p≤0.05$	
Luo M 2015 [73]	Sichuan China	NR	Peripheral blood platelets	Diagnosed	T2D: 43<br>T2D+CHD: 36,<br>CTR: 46	59±9.3<br>64.8±9.8<br>51.4±9.4	23.3±5.4<br>23.4±4.6<br>21.9±2.9	7.0±1.3<br>7.8±1.1<br>5.±0.5	SYBR Green Master Mix (TaKaRa); ABI PRISM 7500 system	$p<0.05$	1 (0/1)
Luo M 2019 [74]	Sichuan China	Sep 2017 to Oct 2018	Plasma	Diagnosed	T2D:47; T2D+CHD: 27; CHD: 34;<br>CTR: 32	60.5±11.14<br>64.5±6.5<br>60.9±5.3<br>58.6±8.1	24.76±3.29<br>25.02±3.12<br>24.57±3.01<br>24.49±2.30	9.15±1.02<br>9.13±1.01<br>6.28±0.69<br>5.36±0.35	Stem-loop qRT-PCR	$p<0.05$	1 (0/1)
Luo M 2019 [75]	Sichuan China	Aug 2018 to Jan 2019	Plasma	Diagnosed	T2D: 48<br>Pre-diabetic: 47<br>CTR: 50	52.6±9.13<br>49.23±6.45<br>45.62±8.58	25.52±2.89<br>25.47±3.02<br>24.56±3.40	9.16±1.05<br>6.78±0.73<br>5.15±0.32	Stem-loop qRT-PCR	$p<0.05$	2 (1/1)
Mao G 2014 [76]	Jinan China	Jan 2013 to Dec 2013	Serum	Diagnosed	66 (33/33)	35-72<br>30-76	NR	NR	qPCR	$p<0.05$	1 (0/1)
Massaro JD 2019 [77]	Brazil	NR	PBMCs	Diagnosed	72 (32/40) with complication T2D +DN: 5; T2D+DC: 21; T2D+DU:5	59.31±8.56<br>44.55±13.6	21.4–47.45<br>23.8 ± 5.1	8.13±1.70<br>NR	MiSeq Reagent Kit v2 on MiSeq system (Illumina)	$p≤0.05$, $\|FC\|>1.0$, FDR<0.1	10 (3/7)
Massart J 2017 [78]	NR	NR	Skeletal muscle	Diagnosed	22 (12/10)	62±1<br>59±1.5	31.6±1<br>29.0±0.5	6.0±0.2<br>4.6±0.1	TaqMan miRNA Assays	$p<0.05$	2 (2/0)
Matsha TE 2018 [79]	South Africa	NR	Whole blood	Newly diagnosed	T2D: 12<br>pre-diabetic: 12<br>CTR: 12	54.8±7.5<br>53.5±8.5<br>52.1±7.8	33.5±8.9<br>33.3±9.1<br>27.3±5.8	NR	Illumina HiSeq with Agilent 2100 Bioanalyzer	$\|FC\|≥1.3$, $p<0.1$	31 (30/1)<br>15 (6/9)
Mazloom H 2015 [80]	Iran	NR	PBMCs	Diagnosed	40 (20/20)	46.5±5.8<br>47.5±4.4	28.7±4.9<br>26.2±4.0	7.02±0.5<br>5.7±0.7	SYBR Premix EX Taq II (Takara); Rotogene Q (Qiagene)	$p<0.05$	1 (0/1)
Meng S 2012 [81]	Shanghai,	NR	EPCs from	Diagnosed	30 (15/15)	67±8	29±9	NR	miRNA array probes (LC	$p<0.01$	5 (0/5)

	China		PBMCs			65±9	27±7		Sciences) and TaqMan miRNA Assays (Applied BioSystems)		
Meng S 2013 [82]	Shanghai China	NR	EPCs from blood	Diagnosed	40 (20/20)	64±8<br>62±9	28±7<br>26±9	NR	Roche LightCycler 480 system	$p<0.05$	1 (0/1)
Mensà E 2019 [83]	Italy	NR	Plasma	Diagnosed	158 (66/92)	NR	NR	NR	RT q-PCR	$p<0.05$	1 (0/1)
Monfared YK 2020 [84]	Iran	NR	Plasma	Newly diagnosed	80 (40/40)	53.69±5.69<br>33.59±7.58	30.11±1.01<br>25.23±2.43	9.13±0.41<br>4.67±1.22	Platinum SYBR Green qPCR SuperMix-UDG	$p<0.05$	1 (1/0)
Mononen N 2019 [85]	Finland	NR	Whole blood	Diagnosed	T2D: 24<br>Pre-diabetic: 252<br>CTR: 589	45.1±3.5<br>43.2±4.7<br>41.9±4.9	30.7±6.7<br>28.2±5.5<br>25.3±3.9	6.6±1.0<br>5.6±0.3<br>5.4±0.2	TaqMan OpenArray miRNA Panel (Applied BioSystems)	$p<0.05$	4 (3/1)
Ortega FJ 2010 [86]	Girona, Spain	NR	Adipose	Diagnosed	T2D+obese: 9<br>CTR+obese: 13<br>CTR: 6	45±10<br>41±9<br>42±9	44.5±4.9<br>44.5±3.0<br>26.6±2.7	5.7±1.4<br>4.9±0.4<br>4.4±0.2	miRNA microarrays (Agilent Technologies)<br>TaqMan miRNA Assays (Applied BioSystems)	$p<0.05$	8 (4/4)
Ortega FJ 2014 [87]	Girona, Spain	NR	Plasma	Diagnosed	T2D/CTR:30/35<br><br>T2D+obese/obese:18 /10	54±10<br>48.1±10.1<br>57.7±8<br>50.6±14.4	26.4±2.4<br>25.2±1.8<br>33.4±3.3<br>32.2±2.4	7.67±1.46<br>4.73±0.35<br>7.06±2.14<br>4.81±0.33	TaqMan Low-Density Arrays (Applied Biosystems)	$p<0.05$	6 (3/3)
Ou L 2019 [88]	China	Jan to Dec 2016	Gingival tissues	Diagnosed	T2D+P: 63<br>Non-T2D+P: 45<br>CTR: 50	50-60 for all samples	29.43±2.78<br>27.20±5.16<br>24.90±5.16	8.39±0.83<br>5.59±0.41<br>5.61±0.49	Bulge-Loop miRNA qPCR Kit (Ribobio)	$p<0.05$	1 (1/0)
Parrizas M 2015 [89]	NR	NR	Serum	Newly diagnosed	27 (10/17)	NR	NR	NR	384-well panels (Exiqon) LC480 II thermocycler (Roche);	$p<0.05$	3 (2/1)
Parrizas M 2020 [90]	Spain	NR	Serum	Newly diagnosed	T2D: 7<br>pre-diabetic: 17	NR	NR	NR	384-well panels (Exiqon) in 7900HT RT-PCR system	$p<0.05$	4 (2/2)

					CTR: 8				(Thermo Fisher Scientific)		
Pek SLT 2016 [91]	Singapore	Jul 2008 to Mar 2012<br><br>NR	Whole blood;<br><br><br>Visceral adipose tissues	Newly diagnosed;<br><br>Diagnosed	T2D-lean: 8<br>T2D-obese: 8<br>CTR-Lean: 8<br>CTR-obese: 8<br>83 (26/57)	50.5±9.8<br>38.0±8.1<br>42.3±9.3<br>36.6±6.72<br>42.7±12.3<br>38.1±10.2	22.4±2.6<br>37.0±13.0<br>21.3±1.4<br>33.3±5.8<br>39.1±7.4<br>41.1±8.3	NR	LNA qPCR	$p<0.05$	5 (0/5)<br><br><br>1 (0/1)
Peng R 2015 [92]	Chongqing, China	Jan 2012 to Sep 2014	Plasma	Diagnosed	T2D+DN: 20,<br>CTR: 20	52.2±5.1<br>50.3±5.7	NR	7.6±1.0<br>NR	TaqMan miRNA Assays (Applied BioSystems)	$p<0.05$	2 (0/2)
Pescador N 2013 [93]	Madrid, Spain	NR	Serum	Diagnosed	T2D: 13<br>T2D+obese: 16<br>obese: 20<br>CTR: 20	69.40±7.12<br>67.55±11<br>41.7±11.18<br>42.9±12.13	24.86±1.49<br>33.38±2.86<br>42.73±4.67<br>22.7±2.43	NR	MiRCURY LNA microRNA PCR system	FC>5	4 (2/2)
Poddar S 2016 [94]	NR	NR	Skeletal muscle	Diagnosed	6 (3/3)	65-75 for all samples	NR	NR	TaqMan miRNA Assays and 7900 HT RT-PCR system (Applied Biosystems)	$p<0.05$	1 (0/1)
Polina ER 2019 [95]	Brazil	NR	Plasma	Diagnosed	20 each for T2D, T2D+PDR, T2D+NPDR, CTR	63±8 for all T2D groups	NR	8.4±1.5 for all T2D groups	TaqMan miRNA RT-PCR (Thermo Fisher Scientific)	$p<0.05$	1 (0/1)
Prabu P 2015 [96]	India	NR	Serum	Diagnosed	T2D: 49<br>Pre-diabetes: 47<br>CTR: 49	44.4±8.1<br>44.1±7.0<br>44.3±6.9	25.7±3.5<br>24.9±2.9<br>24.5±2.6	7.8±1.6<br>6.3±0.8<br>5.6±0.4	miRCURY LNA & SYBR Green Master Mix (Exiqon); ABI 7000 thermocycler	$p<0.05$	2 (2/0)
Prabu P 2020 [97]	India	NR	Serum	Diagnosed	40 each for T2D, T2D+DEP, CTR	54±6.0<br>54±7.0<br>48±10	25.4±4.9<br>25.5±3.7<br>26.0±4.2	8.8±2.0<br>8.6±2.2<br>5.7±0.45	miRCURY LNA & SYBR Green Master Mix (Exiqon)	$p<0.05$	1 (1/0)
Radović N 2018	Serbia	NR	Gingival	Diagnosed	T2D: 24	33.2	26.93	NR	TaqMan miRNA Assays and	$p<0.05$	2 (2/0)

[98]			crevicular fluid		T2D+CP: 24<br>CTR: 24	54.9<br>33.4	25.45<br>26.37		7500 HT RT-PCR system (Applied Biosystems)		
Ramirez HA 2015 [99]	Miami, USA	NR	Foot skin	Diagnosed	20 (10/10)	62.2±7.6<br>53.6±15.1	NR	NR	miRCURY LNA (Exiqon); Quanta qScript miRNA Quantification System	$p≤0.05$	1 (1/0)
Rawal S 2017 [100]	New Zealand	NR	Right atrial appendage & left ventricular	Diagnosed	T2D+IHD: 8<br>IHD: 8<br>CTR: 5	NR	NR	NR	Quantitative RT-PCR analysis	$p<0.05$	2 (0/2)
Rawal S 2019 [101]	New Zealand	NR	Right atrial appendage & left ventricular	Diagnosed	T2D+IHD: 10<br>IHD: 10<br>CTR: 5	70.2±11.79<br>69.9±10.65<br>NR	28.74±5.59<br>27.51±3.58<br>NR	6.94±1.38<br>5.30±0.44<br>NR	Taqman hybridization probes ((Thermofisher, NZ)	$p<0.05$	1 (1/0)
Regmi A 2019 [102]	Hubei China	May 2014 to Mar 2015	Serum	Diagnosed	T2D: 50<br>T2D+DN: 42<br>CTR: 25	50.3±10.3<br>51.5±9.3<br>47.6±7.9	25.5±4.4<br>24.4±3.4<br>22.8±3.4	8.97±1.72<br>10.08±2.25<br>5.45±0.13	PrimeScript RT reagent kit with SYBR Premix Ex Taq (Takara Japan)	$p<0.05$	4 (2/2)
Rezk NA 2016 [103]	Egypt	NR	Serum	Diagnosed and newly diagnosed	T2D: 100<br>Pre-diabetes: 86<br>CTR: 100	48.2±8.2<br>46.3±7.9<br>45.7±8.4	22.9±5.1<br>21.6±3.8<br>23±3.1	8.7±1.9<br>6.6±0.72<br>5.4±0.6	MiScript SYBR Green PCR (Qiagen); ABI7500 RT-PCR system (Applied Biosystems)	$p<0.05$	1 (0/1)
Riches K 2014 [104]	Leeds, UK	Aug 2008 to Jan 2014	Smooth muscle cells of vessels	Diagnosed	20 (10/10)	NR	NR	NR	TaqMan miRNA Assays and 7500 HT RT-PCR system (Applied Biosystems)	$p<0.05$	2 (2/0)
Rojas LB 2016 [105]	Quito, Ecuador	2009 to 2012	Serum	Diagnosed	96 (56/40) for miR-146a<br>90 (53/37) for miR-574-3p	37-85 for all T2D<br>32-87 for all CTR	22-49 for all T2D<br>23-42 for all CTR	7.0 for all T2D<br>5.6 for all CTR	TaqMan miRNA Assays and 7900 HT RT-PCR system (Applied Biosystems)	$p≤0.05$	2 (0/2)
Rong Y 2013 [106]	Hubei, China	Mar 2011 to Jun 2011	Plasma	Newly diagnosed	180 (90/90)	48.5<br>48	24.58±3.66<br>23.38±2.95	NR	TaqMan miRNA Assays (Applied Biosystems)	$p<0.05$	1 (1/0)

Rubie C 2019 [107]	Germany	NR	PBMCs	Diagnosed	56 (20/36)	56-88 for all samples	NR	NR	TaqMan miRNA RT Kit (Thermo Fisher)	$p<0.05$	1 (0/1)
Santovito D 2014 [108]	NR	NR	Plasma exosome	Diagnosed	30 (18/12)	57.2±9.6<br>48.5±12.4	31.6±5.1<br>32.9±5.4	9.6±1.5<br>5.7±0.5	mirCURY LNA (Exiqon); 7900HT (Applied Biosystems)	$p<0.05$	3 (1/2)
Sebastiani G 2015 [109]	NR	NR	Pancreatic islets	Diagnosed	11 (5/6)	71.2±9.8<br>NR	26.36<br>NR	NR	TaqMan miRNA Assays and 7900 HT RT-PCR system (Applied Biosystems)	$p<0.05$	1 (1/0)
Silva M 2019 [110]	Brazil	Mar 2015 to Nov 2017	Plasma	Diagnosed	111 (91/20) for miR-29b<br>199 (179/20) for miR-200b	61.1±8.2 for all T2D<br>45.5±7.5 for CTR	31.1±6.3 for all T2D<br>NR for CTR	8.4±1.6 for all T2D<br>NR for CTR	StepOnePlus RT-PCR System (Life Technologies)	$p<0.05$	2 (1/1)
Smit-McBride Z 2020 [111]	USA	Jun 2011 to Apr 2014	First: aqueous humor, plasma, vitreous humor<br>validation: plasma	Diagnosed	First: T2D+PDR: 6<br>T2D+NPDR: 4<br>CTR: 10<br>Validation: same sample	NR	NR	NR	Affymetrix miRNA Array 3.0 (Affymetrix, Santa Clara)<br><br>TaqMan assays (ThermoFisher Scientific)	$\|logFC\|\geq1.5$<br>$p<0.05$<br><br>$p<0.05$	3 (3/0)<br>35 (6/29)<br>16 (14/2)<br>28 (0/2)
Spinetti G 2013 [112]	NR	NR	Serum and PACs from PBMCs	Diagnosed	T2D+CLI/CTR:99(101)/18; T2D/CTR:6/5<br>T2D+CLI/CLI:6/6<br>T2D/CTR:52/17	71.2±9.3 for all T2D;<br>77.7±9.7 for CLI;<br>36.7±7.3for all CTR	NR	7.8±1.5 for all T2D	TaqMan-validated RT-PCR (Applied Biosystems)	$p<0.05$	19 (17/2)
Spinetti G 2020 [113]	NR	NR	BM-CD34^{+} cells<br>BM-CD34^{+} cells; plasma, extracellular	Diagnosed	Screening: T2D: 7, T2D+CLI: 6, CTR: 6; Validation1:T2D: 4,T2D+CLI: 4, 5 for	NR	NR	NR	Screening: miRCURY LNA Universal RT-PCR (Exiqon)<br>Validation: QuantStudio 6 Flex RT-PCR with miR	$p<0.05$	16 (8/8)<br><br>3 (0/3)

			vesicles of plasma		CRT; validation2: 4/4; validation3: 9/10; validation4: 4/5				TaqMan probes (Thermo Fisher)		1 (0/1)<br>1 (0/1)<br>1 (0/1)
Stępień EL 2018 [114]	Poland	NR	Ectosomes from plasma	Diagnosed	22 (11/11)	NR	NR	NR	miRCURY LNA PCR Panel with SYBR Green Master Mix Universal RT (Exiqon)	FC>2 & $p<0.05$	8 (7/1)
Sucharita S 2018 [115]	India	NR	Plasma	Diagnosed	60 (30/30)	50.5±6.3<br>42.1±7.8	27.3±4.6<br>27.3±4.7	8.4±2.0<br>5.5±0.4	TaqMan miRNA Assays (Applied Biosystems)	$p<0.05$	1 (1/0)
Sun K 2014 [116]	Xinjiang, China	2010 to 2011	Plasma	Diagnosed	200 (100/100)	51.33±11.75<br>48.55±12.41	26.30±4.08<br>24.44±4.63	NR	TaqMan miRNA Assays (Applied Biosystems)	$p<0.05$	1 (1/0)
Sun Q 2020 [117]	Sichuan China	Jun to Oct 2019	Serum	Newly diagnosed	60 (30/30)	42 – 75 for all samples	NR	NR	RT-qPCR	$p<0.05$	2 (2/0)
Tang WB 2018 [118]	China	Dec 2013 to Mar 2015	Serum	Diagnosed	T2D: 22<br>T2D+Micro:20<br>CTR: 30	59.5±8.7<br>60.7±9.6<br>59.8±12.4	25.1±3.9<br>26.2±3.5<br>24.3±5.6	9.4±2.3<br>8.9±2.3<br>NR	RT-qPCR	$p<0.05$	1 (1/0)<br>1 (1/0)
Togliatto G 2018 [119]	Torino Italy	NR	Carotid vascular tissue	Diagnosed	17 (11/6)	74.4±6.07<br>73.6±4.3	NR	NR	TaqMan miRNA Assays (Applied Biosystems)	$p<0.05$	1 (1/0)
Wang C 2016 [120]	Nanjing, China	Sep 2013 to Mar 2015.	Serum	Diagnosed and newly diagnosed	T2D: 92<br>T2D+complication: 92; CTR: 92<br>n=31 for each group of 4 miRNAs	47.7±13.9<br>52.4±13.1<br>50.2±14.2<br>NR	25.6±4.5<br>25.5±2.9<br>23.6±2.0<br>NR	9.8±2.9<br>10.3±2.7<br>5.3±0.4<br>NR	RT-qPCR	$p<0.05$	5 (5/0)<br>4 (4/0)
Wang H 2012 [121]	Guangxi, China	Mar 2010 to Mar 2011	PBMCs	Diagnosed	116 (30/86)	NR	NR	NR	All-in-One miRNA Q-PCR (GeneCopoeia)	$p<0.05$	1 (1/0)
Wang H 2018 [122]	China	Jan 2016 to	Serum	Diagnosed	56 (36/20)	52.5±7.0	NR	NR	miScript SYBR-Green PCR	$p<0.05$	1 (0/1)

		Jan 2017				55.6±4.5			kit (Qiagen)		
Wang JM 2014 [123]	China	NR	BM-Angiogencc ells	Newly diagnosed	11 (6/5)	56±6.3<br>54±1.8	NR	NR	ABI PRISM 7500 Sequence Detection System (Ambion)	$p<0.05$	1 (0/1)
Wang S 2019 [124]	Jilin China	NR	Serum	Diagnosed	62 (33/29)	57.55±1.34<br>53.52±1.67	25.38±0.46<br>24.17±0.41	8.70±0.21<br>5.38±0.08	FastStart Universal SYBR Green Master (Roche)	$p<0.05$	2 (2/0)
Wang SS 2017 [125]	Beijing China	Mar 2015 to Oct 2015	PBMCs	Newly diagnosed	T2D: 117<br>Pre-diabetic: 74<br>CTR: 105	51.68±8.77<br>51.09±7.48<br>49.26±9.09	27.44±3.08<br>26.70±3.25<br>24.18±2.86	7.51±1.42<br>5.75±0.59<br>5.19±0.42	GeneAmp PCR System 9700; TaqMan miRNA assay (Applied Biosystems)	$p<0.05$	2 (1/1)<br>1 (1/0)
Wang WY 2019 [126]	Tianjin China	Oct 2014 to Jun 2015	Serum	Diagnosed	T2D: 69<br>T2D+CAD: 117<br>CTR: 68	64.29±3.77<br>64.73±8.22<br>62.98±7.42	25.61±5.76<br>26.44±3.31<br>24.08±2.42	6.80±2.41<br>8.22±2.64<br>5.29±0.33	SYBR Premix Ex Taq TM II (TaKaRa) with 7300 q-PCR system (Applied Biosystems)	$p<0.05$	1 (1/0)
Wang X 2014 [127]	Sweden and Iraq	Feb 2010 to Mar 2010	Plasma	Diagnosed	152 (33/119)	55.76±5.8 for all samples	28.33±1.27 for all samples	NR	miRCURY LNA miRNA PCR system (Exiqon)	FC>1.5	9 (9/0)
Widlansky ME 2018 [128]	USA	NR	Arterioles	Diagnosed	38 (18/20)	57±8<br>51±11	37.5±7.6<br>26.5±4.2	7.1±1.6<br>5.3±0.5	TaqMan chemistry with SYBR Green chemistry	$p<0.05$	3 (3/0)
Witkowski M 2020 [129]	Germany	NR	Plasma	Diagnosed	66 (46/20)	65.6±9.3<br>48.7±7.3	30.6<br>27.0±4.2	8.39±1.61<br>5.27±0.2	SYBR Green assays with SYBR Select Master Mix (applied Biosystems)	$p<0.05$	1 (0/1)
Wu L 2015 [130]	Guangzhou China	NR	Serum	Diagnosed	T2D: 25<br>T2D+obesity: 25<br>Obesity: 25<br>CTR: 25	46.7±8.6<br>46.1±12.6<br>42.6±11.8<br>45.5±10.2	23.5±2.1<br>33.1±1.9<br>34.2±1.5<br>22.6±2.6	NR	miRNeasy Mini Kit (Qiagen) 7900HT Fast RT-PCR System (Life Technologies)	$p<0.05$	4 (1/3)
Xavier DJ 2015 [131]	Brazil	NR	PBMCs	Diagnosed	18 (9/9)<br>T2D+Hy/CTR: 9/9	NR	NR	NR	Human miRNAMicroarray kit (Agilent Technologies)	$p<0.01$,<br>PFP<0.05	19 (13/6)<br>6 (4/2)
Xiang Y 2015 [132]	USA	NR	Plasma	Diagnosed	38 (30/8)	NR	NR	NR	qPCR	$p<0.05$	1 (0/1)

Xu Q 2014 [133]	Shanghai China	NR	EPCs from blood	Diagnosed	6 (3/3)	NR	NR	BR	TaqMan miRNA assay kits (Applied Biosystems)	$p<0.05$	1 (0/1)
Yan S 2016 [134]	Guizhou China	NR	Plasma	Newly diagnosed	T2D: 50<br>Pre-diabetic: 50<br>CTR: 50	46.22±6.90<br>44.18±6.13<br>45.52±6.22	25.41±0.32<br>25.39±0.32<br>25.56±0.38	8.69±0.36<br>6.53±0.31<br>5.41±0.29	MiScript SYBR Green PCR (Qiagen); ABI7500 RT-PCR system (Applied Biosystems)	$p<0.05$	3 (1/2)<br>4 (2/2)
Yan YX 2020 [135]	Beijing China	Aug 2016 to Dec 2017	Plasma	Newly diagnosed	362 (179/183)	56.9±10.17<br>55.2±10.65	26.80±3.20<br>24.47±3.34	7.47±1.45<br>5.23±0.42	LightCycler 480 SYBR Green I Master (Roche)	$p<0.05$	1 (1/0)
Yang F 2020 [136]	Jilin China	Aug 2017 to Jun 2018	Blood	Newly diagnosed	6 (3/3)	46.67<br>51.33	27.55<br>24.82	NR	Illumina Hiseq 4000 and 2500/2000 (Illumina Inc.)	$\|logFC\|\geq1.5$<br>$p<0.05$	40 (20/20)
Yang S 2019 [137]	Jiangsu China	NR	Plasma	Diagnosed	150 (75/75)	NR	NR	BR	TaqMan miRNA assay kits with ABI RT-PCR 7900 system (Applied Biosystems)	$p<0.05$	12 (12/2)
Yang TT 2015 [138]	Shandong China	NR	Peripheral blood	Diagnosed	20 each for T2D, T2D+BDR, T2D+PDR, CTR	38-70 for all T2D, 35-65 for CTR	NR	NR	SYBR® Premix Ex Taq (Takara); CFX96TM RT PCR Detection System amplifier	$p<0.05$	1 (1/0)
Yang X 2017 [139]	Tianjin China	Jul 2015 to Mar 2016	Serum	Diagnosed	T2D: 92<br>T2D+micro: 87<br>T2D+macro: 51<br>CTR: 53	55.19±12.58<br>58.28±11.68<br>58.40±11.78<br>58.26±9.50	25.86±2.41<br>25.29±2.34<br>23.39±2.74<br>22.00±1.41	8.61±0.23<br>8.71±0.26<br>6.31±0.40<br>5.37±0.09	Mir-X miRNA qRT-PCR SYBR Kit (Takara)	$p<0.05$	2 (1/1)
Yang Z 2014 [140]	Zhejiang China	NR	Serum	Diagnosed	T2D: 24<br>Pre-diabetes: 20,<br>CTR: 20	51.13±9.21<br>52.63±11.25<br>46.65±16.18	25.27±2.90<br>25.68±2.25<br>25.55±5.27	9.49±2.45<br>6.89±1.19<br>5.98±0.80	Solexa deep sequencing (Illumina); Platinum SYBR Green qPCR (Invitrogen)	$p<0.05$	8 (0/8)
Yang ZM 2016 [141]	Guangzhou, China	Oct 2013 to Dec 2013	Serum	Diagnosed	T2D+SDS: 5,<br>T2D+BSS: 5,<br>CTR: 5	57.0±7.0<br>59.4±9.0<br>NR	NR	NR	miRCURY LNA Universal RT miRNA PCR system (Exiqon)	FC>2.0 or <0.5; $p<0.05$	11 (11/0)
Yang ZM 2017	Guangzhou,	Oct 2013 to	Serum	Diagnosed	15 (10/5)	58.2±7.7	NR	NR	miRCURY LNA Universal RT	FC>3 or	7 (4/3)

[142]	China	Dec 2013				56.4±3.7			miRNA PCR system (Exiqon)	<0.33<br>$p<0.05$	
Ye M 2015 [143]	Shanghai China	NR	EPCs from blood	Diagnosed	40 (20/20)	64±9<br>60±10	24.7±5.1<br>24.7±1.8	NR	TaqMan real-time PCR (Germany)	$p<0.05$	1 (0/1)
Yi B 2016 [144]	Changsha, China	2012 and 2015	PBMCs	Diagnosed	30 each for T2D+Micro, CTR, T2D+Normo, T2D+Macro	NR	NR	NR	Applied Biosystems 7300 Real-time PCR system	$p<0.05$	1 (1/0)
Yin C 2020 [145]	Shandong China	2015 to 2017	Serum	Diagnosed	T2D: 40<br>T2D+NPDR: 60<br>T2D+PDR: 50<br>CTR: 60	50.23±3.81<br>50.73±3.80<br>51.40±3.93<br>50.85±5.20	21.78±1.84<br>22.07±2.732<br>2.61±3.1221<br>.81±2.20	6.61±0.59<br>7.31±0.28<br>8.00±0.34<br>4.92±0.46	SYBR green I Master Mix kit (Invitrogen) and 7300 PCR system (Applied Biosystems)	$p<0.05$	1 (1/0)
Zampetaki A 2010 [146]	Bolzano, Italy	1995 to 2005	Plasma	Diagnosed	160 (80/80)	66.3±8.9<br>66.3±8.9	28.0±4.4<br>25.0±4.0	6.5±1.4<br>5.4±0.3	TaqMan miRNA Assays (Applied Biosystems)	$p<0.05$	13 (1/12)
Zhang J 2017 [147]	Nanjing China	NR	Peripheral blood	Diagnosed	T2D: 93,<br>T2D+DFU: 90<br>CTR: 79	66.22<br>67.19<br>63.04	25.0±3.9<br>23.8±4.3<br>24.1±3.2	NR	Taqman microRNA RT kit (Invitrogen); 7300 PCR System (Thermo Fisher Scientific)	$p<0.05$	1 (0/1)
Zhang JY 2016 [148]	Tianjin China	NR	Plasma	Diagnosed	T2D+DA/CTR: 240/240	NR	NR	NR	TaqMan miRNA Assays (Invitrogen)	$p<0.05$	6 (4/2)
Zhang T 2013 [149]	Shenzhen, China	NR	Plasma	Diagnosed	60 (30/30)	63±8.56<br>61±9	NR	NR	Bio-Rad CFX-96 qPCR system (Bio-Rad)	$p<0.05$	1 (0/1)
Zhang T 2015 [150]	Shenzhen China	2011 to 2013	Plasma	Diagnosed	40 (20/20)	61.20±10.62<br>57.25±9.64	24.53±2.87<br>23.90±2.34	NR	SYBR® Premix Dimer-Eraser kit (TaKaRa); Roche LightCycler RT-PCR	$p<0.05$	1 (0/1)
Zhang Y 2015	Jinan,	May 2012 to	Peripheral blood	Diagnosed	T2D+LLI-LM: 70	60.5±8.53	24.75±3.13	8.25±2.11	SYBR-green fluorescence	$p<0.05$	1 (0/1)

[151]	China	Dec 2013	platelets		T2D+LLI-S: 69<br>Nonidabetic: 60	60.8±8.2<br>51.0±10.2	26.42±3.51<br>22.32±1.73	8.66±1.78<br>5.09±0.41	RT-PCR		
Zhao B 2016 [152]	China	NR	Serum	Diagnosed	42 (21/21)	46.9±2.0<br>37.4±3.3	NR	NR	Quantitative RT-PCR analysis	$p<0.05$	1 (0/1)
Zhao H 2010 [153]	Hong Kong, China	NR	Pancreatic tissue	Diagnosed	55 (40/15)	69±13<br>54±14	NR	NR	TaqMan miRNA Assays (Applied BioSystems)	$p<0.05$	1 (1/0)
Zhou J 2013 [154]	Chongqing, China	2010 to 2012	Whole blood	Diagnosed	16 (7/9)	NR	NR	NR	LC sciences (Houston) TaqMan miRNA Assays (Applied Biosystems)	$p<0.05$	1 (1/0)
Zhu J 2020 [155]	Xinjiang China	2016 to 2018	Serum	Diagnosed	131 (59/72)	48.45±7.36<br>44.56±3.58	28.50±4.69<br>21.94±1.33	NR	miRcute plus miRNA SYBR Green qPCR Kit (cat#FP401, TianGen).	$p<0.05$	2 (2/0)
Zou HL 2017 [156]	Nanjing, China	Apr 2013 to Apr 2015	Plasma	Diagnosed	T2D: 65;<br>T2D+DR: 75<br>CTR: 127	49.28±8.54<br>48.33±8.58<br>47.25±9.75	21.86±1.65<br>21.75±1.14<br>22.08±1.59	8.94±2.35<br>12.41±1.56<br>4.28±0.65	qRT-PCR (Qiagen Company)	$p<0.05$	1 (1/0)

Age, BMI and HbA1c are presented as mean±SD, range or mean. ASC, adipose-derived mesenchymal stem cells; ASO, arteriosclerosis of low extremity artery; BC, breast cancer; BDR, background diabetic retinopathy; BM, bone marrow-derived; BMI, body mass index; BSS, blood stasis syndrome; CAD, coronary artery disease; CHD, coronary heart disease; CLI, critical limb ischemia; CP or P, chronic periodontitis or periodontitis; DA, diabetic atherosclerosis; DC, diabetic complication; DFU, diabetic foot ulcers; DN, diabetic nephropathy; DR, diabetic retinopathy; DU, diabetic neuropathy; ED, erectile dysfunction; EPCs, endothelial progenitor cells; FC, fold change; FDR, false discovery rate; HCC, hepatocellular carcinoma; HF, heart failure; Hy, hyperglycemia; IHD, ischaemic heart disease; LLI-LM, low to moderate lower limb ischemia; LLI-S, severe lower limb ischemia; Macro, macroalbuminuria; Micro, microalbuminuria; MN, membranous nephropathy; Normo, normal albuminuria; NR, not reported; PACs, proangiogenic cells; PBMCs, peripheral blood mononuclear cells; PC, pancreatic cancer; (N)PDR, (non-)proliferative diabetic retinopathy, PFP, prediction of false positive; SAM, significance analysis of microarray; SDS, spleen-deficiency syndrome; T2D, type 2 diabetes

ESM Table 2. Quality assessment according to the MIAME and MIQE guideline

Source of bias	Raw data of hybridization	Actual data processing	Sample annotation and experimental variables	Experiment design	Annotation of array design	Experimental and data processing protocols
Al-Hayali MA 2019	H	L	U	U	L	L
Alicka M 2019	H	L	U	U	L	L
Alipoor B 2017	H	L	U	U	U	L
Al-Kafaji G 2015	H	L	L	L	U	L
Al-Kafaji G 2016	H	L	U	L	U	L
Al-Kafaji G 2017	H	L	L	L	L	L
Avgeris M 2020	H	L	U	L	L	L
Bai L 2019	H	L	U	H	H	L
Bai X 2016	H	L	H	U	U	L
Balasubramanyam M 2011	H	L	L	U	H	L
Baldeon RL 2015	H	L	H	H	H	L
Baltaci OF 2018	H	L	H	H	U	U
Bao L 2015	H	L	H	U	H	L
Blum A 2019	H	L	H	U	U	U
Bork-Jensen J 2015	L	L	H	U	U	U
Brovkina O 2019	H	L	U	H	U	U
Candia PD 2017	L	L	L	H	U	L
Cheng S 2018	H	L	H	H	L	U
Cheng Y 2020	H	U	H	H	U	H
Conserva F 2019	L	L	U	U	L	L
Corral-Fernandez NE 2013	H	L	H	H	U	L
Cui X 2017	H	L	U	H	U	U
Dahlmans D 2017	H	L	U	U	U	L

Dai X 2016	H	L	U	U	H	L
Dangwal S 2015	L	U	U	H	H	U
Deng X 2017	H	U	H	L	U	L
El Samaloty NM 2019	H	L	L	U	U	L
Elemeery MN 2019	H	L	H	H	L	L
Fejes Z 2017	H	L	L	L	U	L
Fomison-Nurse I 2018	H	L	U	H	U	L
Fulzele S 2015	H	U	H	H	H	H
Gallagher IJ 2010	L	L	U	L	L	L
Giannella A 2017	H	L	U	H	U	U
Guglielmi V 2017	H	U	H	U	H	L
Guo J 2017	H	U	H	U	U	L
Han YL 2016	H	L	U	L	L	L
He X 2016	H	L	U	U	L	L
He X 2018	H	L	H	U	U	L
Henriksen TI 2017	L	L	U	U	U	L
Hou X 2016	H	L	U	U	U	U
Hou XY 2016	H	L	H	U	U	L
Jansen F 2016	H	L	L	U	U	L
Jiang Q 2017	H	L	L	U	U	L
Jiang X 2015	H	L	L	L	U	L
Jiao Y 2015	H	L	H	H	H	L
Jones A 2017	H	L	L	H	L	L
Kamalden TA 2017	H	U	U	U	U	U
Karolina DS 2011	L	L	U	U	H	U
Karolina DS 2012	H	L	U	H	H	U

Katayama M 2019	L	L	H	H	L	U
Khan R 2020	L	L	L	U	U	U
Kloting N 2009	H	U	U	H	H	L
Kokkinopoulou I 2019	H	L	U	L	U	L
Kong L 2010	H	L	H	L	H	U
Kong L 2011	H	L	L	L	U	L
Krause C 2020	H	L	U	U	U	L
Kuschnerus K 2019	H	U	H	H	U	U
Lareyre F 2019	H	L	U	H	L	L
Latouche C 2016	L	L	U	H	U	L
Latreille M 2014	L	U	U	H	U	U
Li H 2019	H	L	H	U	U	U
Li MY 2016	H	L	L	U	H	L
Li YB 2017	H	L	U	U	U	L
Lian W 2018	H	L	L	H	L	L
Liang L 2016	L	L	H	U	U	L
Liang YZ 2018	L	L	L	L	L	L
Liang Z 2018	H	L	L	L	L	L
Liu H 2016	H	L	U	U	U	L
Liu Y 2017	H	L	H	H	U	L
Locke JM 2014	H	L	H	H	H	U
Long Y 2017	H	L	U	U	U	L
Lu H 2010	L	L	U	U	L	U
Luo M 2015	H	L	H	L	U	L
Luo M 2019 [74]	H	L	H	H	H	L
Luo M 2019 [75]	H	L	L	H	U	L

Mao G 2014	H	U	H	H	U	L
Massaro JD 2019	L	L	U	L	L	L
Massart J 2017	H	L	U	H	U	U
Matsha TE 2018	H	L	H	H	U	U
Mazloom H 2015	H	U	U	H	U	L
Meng S 2012	H	L	U	U	U	U
Meng S 2013	H	U	U	U	U	U
Mensà E 2019	H	L	H	H	H	L
Monfared YK 2019	H	L	H	U	U	U
Mononen N 2019	H	L	L	H	L	L
Ortega FJ 2010	L	L	U	U	U	L
Ortega FJ 2014	H	L	U	H	U	U
Ou L 2019	H	L	U	U	U	U
Parrizas M 2015	H	L	U	H	L	L
Parrizas M 2019	H	L	U	H	L	L
Pek SLT 2016	H	L	U	H	H	L
Peng R 2015	H	L	H	L	U	L
Pescador N 2013	L	L	L	H	U	U
Poddar S 2016	H	L	H	U	U	U
Polina ER 2019	L	L	U	L	L	L
Prabu P 2015	H	L	L	H	U	L
Prabu P 2020	H	L	L	H	U	L
Radović N 2018	H	L	U	H	U	L
Ramirez HA 2015	L	L	H	U	U	L
Rawal S 2017	H	L	H	H	U	U
Rawal S 2019	H	L	U	H	U	U

Regmi A 2019	H	L	L	U	L	L
Rezk NA 2016	H	U	L	U	U	L
Riches K 2014	H	L	H	H	H	U
Rojas LB 2016	H	L	U	U	H	L
Rong Y 2013	H	L	U	U	U	L
Rubie C 2019	H	L	H	L	U	U
Santovito D 2014	H	L	H	H	H	L
Sebastiani G 2015	H	L	H	L	U	L
Silva M 2019	L	L	U	L	L	L
Smit-McBride Z 2020	L	U	U	U	U	L
Spinetti G 2013	H	L	U	U	U	U
Spinetti G 2020	H	L	H	H	L	L
Stępień EL 2018	H	L	L	U	U	L
Sucharita S 2018	H	L	L	L	U	U
Sun K 2014	H	L	U	U	U	L
Sun Q 2020	H	L	H	H	H	U
Tang WB 2018	H	U	U	H	H	H
Togliatto G 2018	H	L	U	U	U	L
Wang C 2016	H	L	L	L	U	L
Wang H 2012	H	L	H	L	H	U
Wang H 2018	H	L	U	L	U	L
Wang JM 2014	H	L	U	H	U	U
Wang S 2019	H	L	H	H	U	U
Wang SS 2017	H	L	L	L	U	L
Wang WY 2019	H	L	U	L	L	L
Wang X 2014	H	U	U	L	U	L

Widlansky ME 2018	L	L	L	H	U	L
Witkowski M 2020	H	L	U	H	U	L
Wu L 2015	H	L	H	H	U	U
Xavier DJ 2015	L	L	H	H	H	L
Xiang Y 2015	H	L	H	U	H	U
Xu Q 2014	H	U	H	U	H	U
Yan S 2016	H	L	L	L	U	L
Yan YX 2020	H	U	U	U	U	U
Yang F 2020	U	L	H	U	U	L
Yang S 2019	H	U	H	L	U	L
Yang TT 2015	H	U	U	H	H	U
Yang X 2017	H	L	L	L	U	U
Yang Z 2014	H	L	U	L	U	L
Yang ZM 2016	H	L	H	U	H	L
Yang ZM 2017	H	L	U	U	H	L
Ye M 2015	H	L	U	H	U	U
Yi B 2016	H	L	U	H	U	L
Yin C 2020	H	L	U	H	U	L
Zampetaki A 2010	H	L	L	L	H	U
Zhang J 2017	H	L	H	H	U	L
Zhang JY 2016	H	L	H	H	H	L
Zhang T 2013	H	U	H	H	U	U
Zhang T 2015	H	L	H	H	H	U
Zhang Y 2015	H	L	H	L	U	L
Zhao B 2016	H	L	H	H	H	U
Zhao H 2010	H	U	H	L	U	L

Zhou J 2013	H	U	H	U	H	U
Zhu J 2020	H	L	U	U	U	L
Zou HL 2017	H	L	L	L	U	L

Abbreviations: H, high risk; L, low risk; U, unclear risk.

ESM Table 3. Statistically significant dysregulation of miRNAs in type 2 diabetes (n= 60)

	miRNA	No. of sub-studies	No. of samples	Overall effect		
				LogOR	[95%CI]	Adjusted *p* values
Up-regulation	miR-29a-3p	8	352	6.838	[5.4211, 8.2548]	2.77E-19
	miR-375	4	307	7.8447	[5.7837, 9.9057]	7.69E-12
	miR-589-3p	2	889	10.5404	[7.7535, 13.3273]	1.10E-11
	miR-4463	5	138	6.6571	[4.8722, 8.4420]	2.38E-11
	miR-346	3	180	8.2217	[5.9401, 10.5034]	1.45E-10
	miR-9-5p	4	152	6.8044	[4.8003, 8.8084]	2.53E-09
	miR-1303	2	246	9.3691	[6.5826, 12.1555]	3.92E-09
	miR-571	2	246	9.3691	[6.5826, 12.1555]	3.92E-09
	miR-661	2	246	9.3691	[6.5826, 12.1555]	3.92E-09
	miR-770-5p	2	246	9.3691	[6.5826, 12.1555]	3.92E-09
	miR-892b	2	246	9.3691	[6.5826, 12.1555]	3.92E-09
	miR-302a-3p	4	124	6.4617	[4.4525, 8.4709]	2.59E-08
	miR-208a	4	70	5.769	[3.7527, 7.7854]	1.82E-06
	miR-181a-2-3p	2	116	7.77	[4.9635, 10.5764]	5.12E-06
	miR-376a-3p	2	69	7.1099	[4.2982, 9.9217]	6.41E-05
	miR-142-5p	2	110	7.5032	[4.3913, 10.6150]	2.04E-04
	miR-140-3p	3	48	5.576	[3.2412, 7.9109]	2.55E-04
	miR-19b-3p	3	49	5.5337	[3.1943, 7.8730]	3.16E-04
	miR-148a-3p	4	913	7.7662	[4.4735, 11.0589]	3.37E-04
	miR-32-5p	2	45	6.2739	[3.4413, 9.1064]	1.26E-03
	miR-187-3p	2	40	6.0845	[3.2472, 8.9218]	2.34E-03
	miR-589	2	107	7.3325	[3.8801, 10.7850]	2.80E-03
	miR-136-5p	2	37	5.7482	[2.8907, 8.6058]	7.17E-03
	miR-4695-5p	2	32	5.6095	[2.7527, 8.4663]	1.06E-02
	miR-29c-3p	8	178	4.8667	[2.3283, 7.4050]	1.52E-02
	miR-296-5p	2	29	5.4174	[2.5522, 8.2826]	1.88E-02
	miR-302b-3p	2	28	5.3521	[2.4830, 8.2212]	2.28E-02
	miR-483-3p	2	26	5.2619	[2.3911, 8.1327]	2.91E-02
	miR-455-3p	2	28	5.2417	[2.3620, 8.1214]	3.20E-02
	miR-548o-3p	2	30	5.2248	[2.3322, 8.1173]	3.56E-02
	miR-320a	14	867	5.2885	[2.2857, 8.2914]	4.96E-02
Down-regulation	miR-30c-5p	6	305	7.4205	[5.7924, 9.0485]	3.68E-17
	miR-1-3p	4	347	8.9444	[6.9732, 10.9155]	5.27E-17
	miR-198	2	850	12.1054	[9.3304, 14.8805]	1.09E-15
	miR-593	4	200	7.8637	[5.8846, 9.8427]	6.08E-13
	miR-103b	3	269	9.0039	[6.7282, 11.2797]	7.89E-13
	miR-152-3p	3	192	8.2663	[5.9844, 10.5483]	1.11E-10
	miR-10b-5p	3	150	7.4974	[5.2028, 9.7920]	1.34E-08
	miR-133a-3p	2	169	8.8885	[6.1004,11.6766]	3.69E-08
	miR-423-3p	4	149	6.5331	[4.4836, 8.5826]	3.70E-08

	miR-374b-5p	3	134	7.1157	[4.8121, 9.4194]	1.25E-07
	miR-200b-3p	2	242	8.5849	[5.7880, 11.3818]	1.59E-07
	miR-4477a	2	133	8.3626	[5.5690, 11.1563]	3.95E-07
	miR-4714-3p	2	133	8.3626	[5.5690, 11.1563]	3.95E-07
	miR-378	4	64	5.6664	[3.6496, 7.6832]	3.25E-06
	miR-126a	2	93	7.5237	[4.7159, 10.3316]	1.34E-05
	miR-182-5p	3	56	5.8221	[3.4929, 8.1512]	8.56E-05
	miR-1228-3p	3	37	5.0521	[2.6954, 7.4087]	2.36E-03
	miR-766-3p	3	35	4.9924	[2.6343, 7.3505]	2.96E-03
	miR-652-3p	2	111	7.3736	[3.8398, 10.9074]	3.84E-03
	miR-1281	3	34	4.9235	[2.5616, 7.2853]	3.91E-03
	miR-660-5p	3	35	4.9138	[2.5467, 7.2809]	4.21E-03
	miR-409-3p	2	37	5.8618	[3.0131, 8.7104]	4.90E-03
	miR-1246	3	30	4.7846	[2.4197, 7.1495]	6.52E-03
	miR-1275	2	30	5.44	[2.5724, 8.3076]	1.78E-02
	miR-1973	2	30	5.44	[2.5724, 8.3076]	1.78E-02
	miR-199b-5p	2	104	7.1309	[3.2754, 10.9865]	2.57E-02
	miR-331-3p	2	104	7.1309	[3.2754, 10.9865]	2.57E-02
	miR-18b-5p	2	26	5.1858	[2.3077, 8.0638]	3.68E-02
	miR-30a-3p	2	25	5.1408	[2.2614, 8.0203]	4.16E-02

ESM Table 4. Statistically significant dysregulation of miRNAs in pancreas (n=1)

	miRNA	No. of sub-studies	No. of samples	Overall effect		
				LogOR	[95%CI]	Adjusted *p* values
Up-regulation	miR-187-3p	2	40	6.0845	[3.2472-8.9218]	2.63E-05

ESM Table 5. Statistically significant dysregulation of miRNAs in heart (n=2)

	miRNA	No. of sub-studies	No. of samples	Overall effect		
				LogOR	[95%CI]	Adjusted *p* values
Up-regulation	miR-208a	4	70	5.7690	[3.7527, 7.7854]	4.10E-08
Down-regulation	miR-15b-5p	3	42	5.3777	[3.0372, 7.7181]	1.34E-05

ESM Table 6. Statistically significant dysregulation of miRNAs in kidney (n=1)

	miRNA	No. of sub-studies	No. of samples	Overall effect		
				LogOR	[95%CI]	Adjusted *p* values
Up-regulation	miR-1228-3p	3	37	5.0521	[2.6954, 7.4087]	1.09E-03

ESM Table 7. Statistically significant dysregulation of miRNAs in adipose (n=6)

	miRNA	No. of sub-studies	No. of samples	Overall effect		
				LogOR	[95%CI]	Adjusted *p* values
Up-regulation	miR-125b-5p	2	85	7.051	[4.0961, 10.0059]	1.75E-05
	miR-140-3p	2	24	5.1299	[2.2535, 8.0063]	2.84E-03
	miR-145-5p	2	24	5.1299	[2.2535, 8.0063]	2.84E-03
	miR-146a-5p	2	24	5.1299	[2.2535, 8.0063]	2.84E-03
	miR-16-5p	2	24	5.1299	[2.2535, 8.0063]	2.84E-03
	miR-24-3p	2	24	5.1299	[2.2535, 8.0063]	2.84E-03

ESM Table 8. Statistically significant dysregulation of miRNAs in muscle (n=5)

	miRNA	No. of sub-studies	No. of samples	Overall effect		
				LogOR	[95%CI]	Adjusted *p* values
Up-regulation	miR-143-3p	2	112	7.6017	[4.6859-10.5174]	1.61E-06
Down-regulation	miR-10b-5p	2	114	7.6901	[4.8813-10.4989]	4.02E-07
	miR-10a-5p	2	108	7.3939	[4.0640-10.7237]	6.75E-05
	miR-27b-3p	2	108	7.3939	[4.0640-10.7237]	6.75E-05
	miR-363-3p	2	33	5.6293	[2.7711-8.4876]	5.65E-04

ESM Table 9. Statistically significant dysregulation of miRNAs in blood (n=50)

	miRNA	No. of sub-studies	No. of samples	Overall effect		
				LogOR	[95%CI]	Adjusted *p* values
Up-regulation	miR-29a-3p	4	272	7.8323	[5.8456, 9.8190]	6.71E-13
	miR-589-3p	2	889	10.5404	[7.7535, 13.3273]	7.56E-12
	miR-346	3	180	8.2217	[5.9401, 10.5034]	9.94E-11
	miR-9-5p	4	152	6.8044	[4.8003, 8.8084]	1.73E-09
	miR-770-5p	2	246	9.3691	[6.5826, 12.1555]	2.68E-09
	miR-661	2	246	9.3691	[6.5826, 12.1555]	2.68E-09
	miR-1303	2	246	9.3691	[6.5826, 12.1555]	2.68E-09
	miR-571	2	246	9.3691	[6.5826, 12.1555]	2.68E-09
	miR-892b	2	246	9.3691	[6.5826, 12.1555]	2.68E-09
	miR-4463	4	112	6.686	[4.6910, 8.6810]	3.10E-09
	miR-572	2	200	9.2302	[6.4447, 12.0157]	5.08E-09
	miR-128-3p	2	160	8.7889	[6.0000, 11.5778]	4.00E-08
	miR-302a-3p	3	112	6.8914	[4.5807, 9.2022]	3.09E-07
	miR-374a-5p	2	120	7.7535	[4.8930, 10.6139]	6.59E-06
	miR-375	3	252	7.836	[4.9199, 10.7521]	8.48E-06
	miR-376a-3p	2	69	7.1099	[4.2982, 9.9217]	4.39E-05
	miR-320c	3	49	5.6282	[3.2948, 7.9617]	1.38E-04
	miR-148a-3p	4	913	7.7662	[4.4735, 11.0589]	2.31E-04
	miR-193b-3p	2	49	6.4626	[3.6362, 9.2890]	4.52E-04
	miR-101-3p	2	45	6.2739	[3.4413, 9.1064]	8.66E-04
	miR-32-5p	2	45	6.2739	[3.4413, 9.1064]	8.66E-04
	miR-1260a	2	42	6.165	[3.3298, 9.0002]	1.24E-03
	miR-19b-3p	2	39	5.9061	[3.0575, 8.7546]	2.95E-03
	miR-136-5p	2	37	5.7482	[2.8907, 8.6058]	4.92E-03
	miR-302b-3p	2	28	5.3521	[2.4830, 8.2212]	1.56E-02
	miR-548o-3p	2	30	5.2248	[2.3322, 8.1173]	2.44E-02
	miR-335-5p	2	165	7.8129	[3.4098, 12.2160]	3.05E-02
	miR-132-3p	2	23	5.0466	[2.1656, 7.9277]	3.63E-02
	miR-135a-5p	2	90	6.8317	[2.9193, 10.7441]	3.78E-02
	miR-188-3p	2	26	5.0382	[2.1401, 7.9363]	4.00E-02
Down-regulation	miR-1-3p	4	347	8.9444	[6.9732, 10.9155]	3.61E-17
	miR-30c-5p	5	295	7.9181	[6.1442, 9.6919]	1.32E-16
	miR-198	2	850	12.1054	[9.3304, 14.8805]	7.50E-16
	miR-593	4	200	7.8637	[5.8846, 9.8427]	4.17E-13
	miR-103b	3	269	9.0039	[6.7282, 11.2797]	5.41E-13
	miR-197-3p	3	283	8.6733	[6.3905, 10.9560]	5.83E-12
	miR-200b-3p	2	242	8.5849	[5.7880, 11.3818]	1.09E-07
	miR-4477a	2	133	8.3626	[5.5690, 11.1563]	2.71E-07
	miR-4714-3p	2	133	8.3626	[5.5690, 11.1563]	2.71E-07
	miR-378	4	64	5.6664	[3.6496, 7.6832]	2.23E-06

miR-152-3p	2	100	7.8637	[5.0648, 10.6625]	2.23E-06
miR-126a	2	93	7.5237	[4.7159, 10.3316]	9.21E-06
miR-503-5p	2	69	7.1099	[4.2982, 9.9217]	4.39E-05
miR-182-5p	3	56	5.8221	[3.4929, 8.1512]	5.87E-05
miR-423-3p	3	57	5.6437	[3.3040, 7.9835]	1.38E-04
miR-374b-5p	2	42	6.1041	[3.2648, 8.9434]	1.53E-03
miR-652-3p	2	111	7.3736	[3.8398, 10.9074]	2.64E-03
miR-660-5p	3	35	4.9138	[2.5467, 7.2809]	2.89E-03
miR-409-3p	2	37	5.8618	[3.0131, 8.7104]	3.36E-03
miR-214-3p	2	89	6.9567	[3.4471, 10.4663]	6.22E-03

ESM Table 10. Statistically significant dysregulation of miRNAs in PBMCs (n = 12)

	miRNA	No. of sub-studies	No. of samples	Overall effect		
				LogOR	[95%CI]	Adjusted p values
Up-regulation	miR-18a-5p	2	413	10.6376	[7.8589, 13.4163]	7.48E-13
	miR-346	3	180	8.2217	[5.9401, 10.5034]	1.96E-11
	miR-29b-3p	2	36	5.8889	[3.0451, 8.7327]	5.93E-04
	miR-222-3p	2	36	5.8889	[3.0451, 8.7327]	5.93E-04
	miR-186-5p	2	36	5.8889	[3.0451, 8.7327]	5.93E-04
	miR-188-3p	2	26	5.0382	[2.1401, 7.9363]	7.87E-03
	miR-20a-5p	2	26	5.0382	[2.1401, 7.9363]	7.87E-03
Down-regulation	miR-146a-5p	3	140	7.6934	[5.4058, 9.9810]	5.22E-10
	miR-144-5p	2	133	8.3626	[5.5690, 11.1563]	5.33E-08
	miR-4477a	2	133	8.3626	[5.5690, 11.1563]	5.33E-08
	miR-4714-3p	2	133	8.3626	[5.5690, 11.1563]	5.33E-08
	miR-155-5p	2	80	7.4271	[4.6217, 10.2326]	2.54E-06

ESM Table 11. Statistically significant dysregulation of miRNAs in whole blood (n = 25)

	miRNA	No. of sub-studies	No. of samples	Overall effect		
				LogOR	[95%CI]	Adjusted p values
Up-regulation	miR-122-5p	2	889	10.5404	[7.7535, 13.3273]	3.10E-12
	miR-589-3p	2	889	10.5404	[7.7535, 13.3273]	3.10E-12
	miR-155-5p	3	120	7.4271	[5.1365, 9.7177]	5.20E-09
	miR-103a-3p	2	120	7.8123	[5.0062, 10.6185]	1.22E-06
	miR-15b-5p	2	48	6.4378	[3.6111, 9.2645]	2.01E-04
	miR-26b-5p	2	39	5.9944	[3.1524, 8.8363]	8.90E-04
	miR-151a-5p	2	39	5.9944	[3.1524, 8.8363]	8.90E-04
	miR-150-5p	2	111	7.3736	[3.8398, 10.9074]	1.08E-03
	miR-192-5p	2	111	7.3736	[3.8398, 10.9074]	1.08E-03
	miR-27a-3p	2	111	7.3736	[3.8398, 10.9074]	1.08E-03
	miR-320a	2	111	7.3736	[3.8398, 10.9074]	1.08E-03
	miR-148a-3p	3	895	8.3771	[4.0588, 12.6953]	3.58E-03
	miR-548o-3p	2	30	5.2248	[2.3322, 8.1173]	1.00E-02
	miR-151a-3p	2	21	4.7465	[1.8376, 7.6554]	3.45E-02
Down-regulation	miR-24-3p	4	211	7.6194	[5.6304, 9.6085]	1.50E-12
	miR-378	4	64	5.6664	[3.6496, 7.6832]	9.13E-07
	miR-486-5p	2	48	6.4378	[3.6111, 9.2645]	2.01E-04
	miR-342-3p	2	48	6.305	[3.4702, 9.1397]	3.25E-04
	miR-17-5p	2	111	7.3736	[3.8398, 10.9074]	1.08E-03
	miR-652-3p	2	111	7.3736	[3.8398, 10.9074]	1.08E-03
	miR-210-3p	2	32	5.6664	[2.8143, 8.5186]	2.47E-03
	let-7f-5p	2	89	6.9567	[3.4471, 10.4663]	2.55E-03
	miR-214-3p	2	89	6.9567	[3.4471, 10.4663]	2.55E-03
	miR-20a-5p	2	21	4.7465	[1.8376, 7.6554]	3.45E-02
	miR-660-5p	2	21	4.7465	[1.8376, 7.6554]	3.45E-02

ESM Table 12. Statistically significant dysregulation of miRNAs in serum (n = 38)

	miRNA	No. of sub-studies	No. of samples	Overall effect		
				LogOR	[95%CI]	Adjusted p values
Up-regulation	miR-210-3p	4	540	9.6747	[7.7066, 11.6429]	2.29E-20
	miR-221-3p	6	793	8.7013	[6.8069, 10.5957]	8.80E-18
	miR-29a-3p	3	257	8.5626	[6.2811, 10.8441]	7.60E-12
	miR-15a-5p	4	209	6.8546	[4.8481, 8.8610]	8.60E-10
	miR-125b-5p	2	246	9.3691	[6.5826, 12.1555]	1.76E-09
	miR-1303	2	246	9.3691	[6.5826, 12.1555]	1.76E-09
	miR-571	2	246	9.3691	[6.5826, 12.1555]	1.76E-09
	miR-661	2	246	9.3691	[6.5826, 12.1555]	1.76E-09
	miR-770-5p	2	246	9.3691	[6.5826, 12.1555]	1.76E-09
	miR-892b	2	246	9.3691	[6.5826, 12.1555]	1.76E-09
	miR-130b-3p	2	194	9.1696	[6.3837, 11.9555]	4.44E-09
	miR-99b-5p	4	162	6.6902	[4.6173, 8.7631]	1.01E-08
	miR-128-3p	2	160	8.7889	[6.0000, 11.5778]	2.62E-08
	miR-302a-3p	3	112	6.8914	[4.5807, 9.2022]	2.02E-07
	miR-9-5p	3	107	6.8083	[4.4964, 9.1202]	3.14E-07
	miR-27b-3p	3	85	6.1699	[3.8419, 8.4978]	8.20E-06
	miR-24-3p	4	200	6.5303	[4.0615, 8.9991]	8.68E-06
	miR-376a-3p	2	69	7.1099	[4.2982, 9.9217]	2.88E-05
	miR-124-3p	2	47	6.0729	[3.2224, 8.9235]	1.19E-03
	miR-194-5p	2	39	5.9049	[3.0577, 8.7521]	1.92E-03
	miR-150-5p	2	49	5.9913	[2.9747, 9.0079]	3.96E-03
	miR-20b-5p	2	49	5.9913	[2.9747, 9.0079]	3.96E-03
	miR-107	2	23	5.0466	[2.1656, 7.9277]	2.39E-02
	miR-132-3p	2	23	5.0466	[2.1656, 7.9277]	2.39E-02
	miR-1304-5p	2	20	4.7958	[1.9007, 7.6908]	4.67E-02
	miR-302d-3p	2	20	4.7958	[1.9007, 7.6908]	4.67E-02
Down-regulation	miR-198	2	850	12.1054	[9.3304, 14.8805]	4.92E-16
	miR-593	4	200	7.8637	[5.8846, 9.8427]	2.73E-13
	miR-1-3p	3	270	9.0217	[6.7461, 11.2973]	3.13E-13
	miR-192-5p	4	381	7.7446	[5.3532, 10.1360]	8.76E-09
	miR-152-3p	2	100	7.8637	[5.0648, 10.6625]	1.46E-06
	miR-186-5p	2	88	7.6054	[4.8027, 10.4081]	4.20E-06
	miR-503-5p	2	69	7.1099	[4.2982, 9.9217]	2.88E-05
	miR-423-3p	3	57	5.6437	[3.3040, 7.9835]	9.08E-05
	let-7i-5p	2	54	6.2461	[3.3984, 9.0939]	6.88E-04
	miR-1246	2	20	4.7958	[1.9007, 7.6908]	4.67E-02
	miR-6131	2	20	4.7958	[1.9007, 7.6908]	4.67E-02
	miR-98-5p	2	20	4.7958	[1.9007, 7.6908]	4.67E-02

ESM Table 13. Statistically significant dysregulation of miRNAs in plasma (n = 25)

	miRNA	No. of sub-studies	No. of samples	Overall effect		
				LogOR	[95%CI]	Adjusted p values
Up-regulation	miR-144-3p	6	951	9.2375	[7.4044, 11.0707]	1.37E-21
	miR-93-5p	4	564	9.3044	[7.2507, 11.3582]	1.75E-17
	miR-34a-5p	5	183	7.0493	[5.2683, 8.8303]	2.25E-13
	miR-4463	4	112	6.686	[4.6910, 8.6810]	1.32E-09
	miR-572	2	200	9.2302	[6.4447, 12.0157]	2.16E-09
	miR-29c-3p	2	95	7.5697	[4.7632, 10.3762]	3.25E-06
	miR-30b-5p	2	172	8.1724	[4.4843, 11.8604]	3.64E-04
	miR-199a-3p	2	42	6.1802	[3.3460, 9.0144]	4.99E-04
	miR-320c	2	34	5.6717	[2.8136, 8.5298]	2.60E-03
Down-regulation	miR-30c-5p	4	280	8.4854	[6.5111, 10.4596]	9.46E-16
	miR-223-3p	4	309	8.4201	[6.4439, 10.3962]	1.76E-15
	miR-130b-3p	3	140	7.5897	[5.2989, 9.8804]	2.18E-09
	miR-200b-3p	2	242	8.5849	[5.7880, 11.3818]	4.65E-08
	miR-185-5p	2	125	8.2932	[5.4995, 11.0870]	1.55E-07
	miR-126-3p	14	1421	7.5404	[4.8784, 10.2024]	7.36E-07
	miR-26a-5p	2	162	8.2401	[5.1650, 11.3151]	3.90E-06
	miR-126a	2	93	7.5237	[4.7159, 10.3316]	3.93E-06
	miR-125b-5p	2	93	7.5237	[4.7159, 10.3316]	3.93E-06
	miR-26b-5p	2	78	7.2781	[4.4681, 10.0880]	9.98E-06
	miR-195-5p	2	85	7.2492	[4.4345, 10.0639]	1.16E-05
	miR-197-3p	2	187	8.4249	[4.9854, 11.8644]	4.11E-05
	miR-192-5p	2	79	6.8486	[3.7805, 9.9167]	3.15E-04
	miR-15b-5p	2	41	5.9617	[3.1116, 8.8118]	1.08E-03
	miR-182-5p	2	41	5.9617	[3.1116, 8.8118]	1.08E-03
	miR-20a-5p	2	30	5.4271	[2.5589, 8.2953]	5.41E-03

ESM Table 14. Statistically significant dysregulation of miRNAs in blood fractions (n=87)

miRNA	Whole blood	PBMCs	Serum	Plasma
let-7f-5p	-1			
let-7i-5p			-1	
miR-1-3p			-1	
miR-103a-3p	1			
miR-107			1	
miR-122-5p	1			
miR-124-3p			1	
miR-1246			-1	
miR-125b-5p			1	-1
miR-126-3p				-1
miR-126a				-1
miR-128-3p			1	
miR-1303			1	
miR-130b-3p			1	-1
miR-132-3p			1	
miR-1304-5p			1	
miR-144-3p				1
miR-144-5p		-1		
miR-146a-5p		-1		
miR-148a-3p	1			
miR-150-5p	1		1	
miR-151a-3p	1			
miR-151a-5p	1			
miR-152-3p			-1	
miR-155-5p	1	-1		
miR-15a-5p			1	
miR-15b-5p	1			-1
miR-17-5p	-1			
miR-182-5p				-1
miR-185-5p				-1
miR-186-5p		1	-1	
miR-188-3p		1		
miR-18a-5p		1		
miR-192-5p	1		-1	-1
miR-194-5p			1	
miR-195-5p				-1
miR-197-3p				-1
miR-198			-1	
miR-199a-3p				1
miR-20a-5p	-1	1		-1
miR-20b-5p			1	
miR-200b-3p				-1

miR-210-3p	-1		1	
miR-214-3p	-1			
miR-221-3p			1	
miR-222-3p		1		
miR-223-3p				-1
miR-24-3p	-1		1	
miR-26a-5p				-1
miR-26b-5p	1			-1
miR-27a-3p	1			
miR-27b-3p			1	
miR-29a-3p			1	
miR-29b-3p		1		
miR-29c-3p				1
miR-30b-5p				1
miR-30c-5p				-1
miR-302a-3p			1	
miR-302d-3p			1	
miR-320a	1			
miR-320c				1
miR-342-3p	-1			
miR-346		1		
miR-34a-5p				1
miR-376a-3p			1	
miR-378	-1			
miR-423-3p			-1	
miR-4463				1
miR-4477a		-1		
miR-4714-3p		-1		
miR-486-5p	-1			
miR-503-5p			-1	
miR-548o-3p	1			
miR-571			1	
miR-572				1
miR-589-3p	1			
miR-593			-1	
miR-6131			-1	
miR-652-3p	-1			
miR-660-5p	-1			
miR-661			1	
miR-770-5p			1	
miR-892b			1	
miR-9-5p			1	
miR-93-5p				1
miR-98-5p			-1	

miR-99b-5p	1

“1” indicates up-regulation and “-1” indicates down-regulation

ESM Table 15. Statistically significant dysregulation of miRNAs detected by PCR-based methods (n = 61)

	miRNA	No. of sub-studies	No. of samples	Overall effect		
				LogOR	[95%CI]	Adjusted p values
Up-regulation	miR-144-3p	12	1105	7.4909	[5.9441, 9.0376]	1.99E-19
	miR-29a-3p	8	352	6.838	[5.4211, 8.2548]	2.74E-19
	miR-29c-3p	7	172	6.1063	[4.5842, 7.6284]	3.30E-13
	miR-375	4	307	7.8447	[5.7837, 9.9057]	7.60E-12
	miR-589-3p	2	889	10.5404	[7.7535, 13.3273]	1.09E-11
	miR-4463	5	138	6.6571	[4.8722, 8.4420]	2.35E-11
	miR-346	3	180	8.2217	[5.9401, 10.5034]	1.43E-10
	miR-1303	2	246	9.3691	[6.5826, 12.1555]	3.87E-09
	miR-571	2	246	9.3691	[6.5826, 12.1555]	3.87E-09
	miR-661	2	246	9.3691	[6.5826, 12.1555]	3.87E-09
	miR-770-5p	2	246	9.3691	[6.5826, 12.1555]	3.87E-09
	miR-892b	2	246	9.3691	[6.5826, 12.1555]	3.87E-09
	miR-302a-3p	3	114	6.9902	[4.6842, 9.2962]	2.49E-07
	miR-148a-3p	3	907	9.0134	[5.9592, 12.0675]	6.42E-07
	miR-9-5p	3	107	6.8083	[4.4964, 9.1202]	6.90E-07
	miR-208a	4	70	5.769	[3.7527, 7.7854]	1.80E-06
	miR-376a-3p	2	69	7.1099	[4.2982, 9.9217]	6.34E-05
	miR-142-5p	2	110	7.5032	[4.3913, 10.6150]	2.02E-04
	miR-19b-3p	3	49	5.5337	[3.1943, 7.8730]	3.12E-04
	miR-30b-5p	3	182	7.0629	[3.9743, 10.1516]	6.51E-04
	miR-32-5p	2	45	6.2739	[3.4413, 9.1064]	1.25E-03
	miR-187-3p	2	40	6.0845	[3.2472, 8.9218]	2.31E-03
	miR-589	2	107	7.3325	[3.8801, 10.7850]	2.77E-03
	miR-136-5p	2	37	5.7482	[2.8907, 8.6058]	7.09E-03
	miR-4695-5p	2	32	5.6095	[2.7527, 8.4663]	1.05E-02
	miR-296-5p	2	29	5.4174	[2.5522, 8.2826]	1.85E-02
	miR-483-3p	2	26	5.2619	[2.3911, 8.1327]	2.88E-02
	miR-455-3p	2	28	5.2417	[2.3620, 8.1214]	3.17E-02
	miR-140-3p	2	24	5.1299	[2.2535, 8.0063]	4.16E-02
	miR-320a	14	867	5.2885	[2.2857, 8.2914]	4.90E-02
Down-regulation	miR-30c-5p	6	305	7.4205	[5.7924, 9.0485]	3.63E-17
	miR-1-3p	4	347	8.9444	[6.9732, 10.9155]	5.21E-17
	miR-198	2	850	12.1054	[9.3304, 14.8805]	1.08E-15
	miR-100-5p	6	239	6.7735	[5.1393, 8.4077]	3.98E-14
	miR-593	4	200	7.8637	[5.8846, 9.8427]	6.01E-13
	miR-103b	3	269	9.0039	[6.7282, 11.2797]	7.81E-13
	miR-152-3p	3	192	8.2663	[5.9844, 10.5483]	1.10E-10
	miR-10b-5p	3	150	7.4974	[5.2028, 9.7920]	1.33E-08
	miR-133a-3p	2	169	8.8885	[6.1004,11.6766]	3.65E-08

	miR-374b-5p	3	134	7.1157	[4.8121, 9.4194]	1.24E-07
	miR-200b-3p	2	242	8.5849	[5.7880, 11.3818]	1.58E-07
	miR-423-3p	3	139	7.0851	[4.6864, 9.4838]	6.22E-07
	miR-98-5p	3	179	7.5765	[4.9474, 10.2056]	1.43E-06
	miR-378	4	64	5.6664	[3.6496, 7.6832]	3.21E-06
	miR-126a	2	93	7.5237	[4.7159, 10.3316]	1.33E-05
	miR-363-3p	4	55	5.2327	[3.1969, 7.2685]	4.14E-05
	miR-182-5p	3	56	5.8221	[3.4929, 8.1512]	8.47E-05
	miR-30e-3p	2	49	6.4626	[3.6362, 9.2890]	6.52E-04
	miR-1228-3p	3	37	5.0521	[2.6954, 7.4087]	2.33E-03
	miR-766-3p	3	35	4.9924	[2.6343, 7.3505]	2.93E-03
	miR-652-3p	2	111	7.3736	[3.8398, 10.9074]	3.80E-03
	miR-1281	3	34	4.9235	[2.5616, 7.2853]	3.86E-03
	miR-409-3p	2	37	5.8618	[3.0131, 8.7104]	4.85E-03
	miR-1275	2	30	5.44	[2.5724, 8.3076]	1.76E-02
	miR-1973	2	30	5.44	[2.5724, 8.3076]	1.76E-02
	miR-660-5p	2	29	5.3927	[2.5242, 8.2612]	2.02E-02
	miR-199b-5p	2	104	7.1309	[3.2754, 10.9865]	2.54E-02
	miR-331-3p	2	104	7.1309	[3.2754, 10.9865]	2.54E-02
	miR-223-3p	8	392	5.9465	[2.7269, 9.1661]	2.59E-02
	miR-18b-5p	2	26	5.1858	[2.3077, 8.0638]	3.63E-02
	miR-30a-3p	2	25	5.1408	[2.2614, 8.0203]	4.11E-02

ESM Table 16. Statistically significant dysregulation of miRNAs detected by RNA-Seq (n=11)

	miRNA	No. of sub-studies	No. of samples	Overall effect		
				LogOR	[95%CI]	Adjusted *p* values
Up-regulation	miR-15b-5p	2	48	6.4378	[3.6111, 9.2645]	8.86E-05
	miR-99b-5p	2	34	5.6364	[2.7761, 8.4967]	1.23E-03
	miR-548o-3p	2	30	5.2248	[2.3322, 8.1173]	4.40E-03
Down-regulation	miR-144-3p	2	133	8.3626	[5.5690, 11.1563]	4.88E-08
	miR-144-5p	2	133	8.3626	[5.5690, 11.1563]	4.88E-08
	miR-4477a	2	133	8.3626	[5.5690, 11.1563]	4.88E-08
	miR-4714-3p	2	133	8.3626	[5.5690, 11.1563]	4.88E-08
	miR-486-5p	3	58	5.9077	[3.5816, 8.2339]	7.07E-06
	miR-126-5p	2	16	4.3543	[1.4258, 7.2828]	3.93E-02
	miR-16-5p	2	16	4.3543	[1.4258, 7.2828]	3.93E-02
	miR-30b-5p	2	16	4.3543	[1.4258, 7.2828]	3.93E-02

ESM Table 17. Statistically significant dysregulation of miRNAs in sensitivity analysis based on sample sizes (n=90)

Sample size	Direction of regulation	miRNA	No. of sub-studies	Pooled sample size	Overall effect		
					LogOR	[95%CI]	Adjusted *p* values
No less than 25	Up- regulation	miR-93-5p	5	716	9.7747	[8.0145, 11.5348]	7.67E-26
		miR-210-3p	4	540	9.6747	[7.7066, 11.6429]	3.20E-20
		miR-29a-3p	4	295	8.2552	[6.2771, 10.2333]	1.60E-15
		miR-222-3p	4	203	7.7746	[5.7929, 9.7562]	8.29E-13
		miR-30d-5p	3	313	8.7249	[6.4439, 11.0060]	3.67E-12
		miR-148a-3p	2	889	10.5404	[7.7535, 13.3273]	6.94E-12
		miR-589-3p	2	889	10.5404	[7.7535, 13.3273]	6.94E-12
		miR-375	3	292	8.5832	[6.3000, 10.8664]	9.69E-12
		miR-346	3	180	8.2217	[5.9401, 10.5034]	9.13E-11
		miR-1303	2	246	9.3691	[6.5826, 12.1555]	2.46E-09
		miR-661	2	246	9.3691	[6.5826, 12.1555]	2.46E-09
		miR-770-5p	2	246	9.3691	[6.5826, 12.1555]	2.46E-09
		miR-892b	2	246	9.3691	[6.5826, 12.1555]	2.46E-09
		miR-571	2	246	9.3691	[6.5826, 12.1555]	2.46E-09
		miR-572	2	200	9.2302	[6.4447, 12.0157]	4.66E-09
		miR-9-5p	3	142	7.4373	[5.1390, 9.7355]	1.27E-08
		miR-4463	3	90	6.8025	[4.5007, 9.1044]	3.89E-07
		miR-146b-5p	2	221	8.8849	[5.8523, 11.9175]	5.23E-07
		miR-302a-3p	2	102	7.871	[5.0718, 10.6702]	1.99E-06
		miR-451a	2	119	7.8737	[5.0699, 10.6776]	2.08E-06
		miR-29c-3p	2	95	7.5697	[4.7632, 10.3762]	7.00E-06
		miR-376a-3p	2	69	7.1099	[4.2982, 9.9217]	4.03E-05

	miR-4306	2	64	6.9324	[4.1164, 9.7485]	7.84E-05
	miR-19a-3p	2	187	8.4249	[4.9854, 11.8644]	8.85E-05
Down-regulation	miR-223-3p	5	343	8.16	[6.3904, 9.9296]	8.96E-18
	miR-1-3p	4	347	8.9444	[6.9732, 10.9155]	3.32E-17
	miR-197-3p	4	353	8.6362	[6.6601, 10.6123]	6.05E-16
	miR-198	2	850	12.1054	[9.3304, 14.8805]	6.89E-16
	miR-30c-5p	4	280	8.4854	[6.5111, 10.4596]	2.04E-15
	miR-144-5p	3	746	9.2328	[6.9538, 11.5119]	1.13E-13
	miR-593	4	200	7.8637	[5.8846, 9.8427]	3.82E-13
	miR-103b	3	269	9.0039	[6.7282, 11.2797]	4.97E-13
	miR-23a-3p	4	211	7.7607	[5.7785, 9.7428]	9.35E-13
	miR-26b-5p	4	176	7.54	[5.5565, 9.5234]	5.20E-12
	miR-152-3p	3	192	8.2663	[5.9844, 10.5483]	7.00E-11
	miR-320b	2	200	9.2302	[6.4447, 12.0157]	4.66E-09
	let-7f-5p	3	134	7.4077	[5.1138, 9.7017]	1.38E-08
	miR-130a-3p	3	116	7.3221	[5.0293, 9.6148]	2.17E-08
	miR-96-5p	3	133	7.3394	[5.0404, 9.6385]	2.20E-08
	miR-100-5p	2	175	8.8902	[6.1015, 11.6790]	2.32E-08
	miR-133a-3p	2	169	8.8885	[6.1004,11.6766]	2.32E-08
	miR-98-5p	2	169	8.8885	[6.1004, 11.6766]	2.32E-08
	miR-200b-3p	2	242	8.5849	[5.7880, 11.3818]	1.00E-07
	miR-4477a	2	133	8.3626	[5.5690, 11.1563]	2.49E-07
	miR-4714-3p	2	133	8.3626	[5.5690, 11.1563]	2.49E-07
	miR-423-3p	2	129	8.1601	[5.3621, 10.9580]	6.10E-07
	miR-10b-5p	2	128	8.097	[5.2971, 10.8969]	8.06E-07
	miR-342-3p	2	125	8.0642	[5.2646, 10.8637]	9.18E-07

		miR-374b-5p	2	119	7.8737	[5.0699, 10.6776]	2.08E-06
		miR-186-5p	2	88	7.6054	[4.8027, 10.4081]	5.88E-06
		miR-26a-5p	2	162	8.2401	[5.1650, 11.3151]	8.40E-06
		miR-126a	2	93	7.5237	[4.7159, 10.3316]	8.46E-06
		miR-126-3p	24	2748	5.8539	[2.9560, 8.7519]	4.21E-03
No less than 50	Up-regulation	miR-93-5p	5	716	9.7747	[8.0145, 11.5348]	5.34E-26
		miR-221-3p	5	781	9.3868	[7.6232, 11.1503]	6.90E-24
		miR-210-3p	4	540	9.6747	[7.7066, 11.6429]	2.23E-20
		miR-148a-3p	2	889	10.5404	[7.7535, 13.3273]	4.84E-12
		miR-589-3p	2	889	10.5404	[7.7535, 13.3273]	4.84E-12
		miR-27a-3p	3	218	8.5517	[6.2725, 10.8309]	7.49E-12
		miR-99b-5p	3	212	8.4823	[6.2024, 10.7622]	1.19E-11
		miR-346	3	180	8.2217	[5.9401, 10.5034]	6.36E-11
		miR-222-3p	3	175	8.1404	[5.8577, 10.4231]	1.08E-10
		miR-15b-5p	2	275	9.7437	[6.9607, 12.5267]	2.65E-10
		miR-30d-5p	2	276	9.4392	[6.6525, 12.2258]	1.23E-09
		miR-1303	2	246	9.3691	[6.5826, 12.1555]	1.72E-09
		miR-661	2	246	9.3691	[6.5826, 12.1555]	1.72E-09
		miR-770-5p	2	246	9.3691	[6.5826, 12.1555]	1.72E-09
		miR-892b	2	246	9.3691	[6.5826, 12.1555]	1.72E-09
		miR-571	2	246	9.3691	[6.5826, 12.1555]	1.72E-09
		miR-572	2	200	9.2302	[6.4447, 12.0157]	3.24E-09
		miR-375	2	255	9.2294	[6.4389, 12.0199]	3.52E-09
		miR-29a-3p	2	220	9.1972	[6.4098, 11.9846]	3.90E-09
		miR-302a-3p	2	102	7.871	[5.0718, 10.6702]	1.39E-06
		miR-138-5p	2	100	7.8637	[5.0648, 10.6625]	1.43E-06

	Down-regulation	miR-1-3p	4	347	8.9444	[6.9732, 10.9155]	2.31E-17
		miR-198	2	850	12.1054	[9.3304, 14.8805]	4.80E-16
		miR-30c-5p	4	280	8.4854	[6.5111, 10.4596]	1.42E-15
		miR-197-3p	3	326	9.2808	[7.0061, 11.5554]	4.99E-14
		miR-144-5p	3	746	9.2328	[6.9538, 11.5119]	7.88E-14
		miR-593	4	200	7.8637	[5.8846, 9.8427]	2.66E-13
		miR-103b	3	269	9.0039	[6.7282, 11.2797]	3.46E-13
		miR-223-3p	3	262	8.6563	[6.3763, 10.9364]	3.90E-12
		miR-152-3p	3	192	8.2663	[5.9844, 10.5483]	4.88E-11
		miR-320b	2	200	9.2302	[6.4447, 12.0157]	3.24E-09
		miR-100-5p	2	175	8.8902	[6.1015, 11.6790]	1.62E-08
		miR-133a-3p	2	169	8.8885	[6.1004,11.6766]	1.62E-08
		miR-98-5p	2	169	8.8885	[6.1004, 11.6766]	1.62E-08
		miR-4477a	2	133	8.3626	[5.5690, 11.1563]	1.73E-07
		miR-4714-3p	2	133	8.3626	[5.5690, 11.1563]	1.73E-07
		miR-26b-5p	2	102	7.8932	[5.0946, 10.6918]	1.26E-06

ESM Table 18. The characteristics of databases for human microRNA-target gene interactions

Database	miRTarBase	miRecords	TransmiR
No. of miRNA-target interactions	380639	1685	2255
No. of microRNAs	2599	224	402
No. of target genes	15064	1097	369
No. of articles	7204	541	1075
Release date of the latest version	15 Sept 2021	27 Apr 2013	May 2018
Present version	9.0	4.0	2.0

ESM Table 19. Pathway analysis on dysregulated microRNAs from meta-analyses with newly built microRNA regulated pathways

Pathway name	Original ID	totalAcc	pPert	pORA	pComb	pPert.fdr	pORA.fdr	pComb.fdr
MAPK signaling pathway	hsa04010	587.8251	0.0165	3.10E-37	3.12E-07	0.3670	9.84E-36	7.68E-06
Regulation of actin cytoskeleton	hsa04810	601.8163	0.0140	1.90E-28	2.67E-07	0.3568	1.40E-27	7.68E-06
Diabetic cardiomyopathy	hsa05415	338.1408	0.0110	4.68E-25	2.12E-07	0.3495	1.96E-24	7.68E-06
Chemokine signaling pathway	hsa04062	381.1079	0.0180	1.80E-23	3.39E-07	0.3670	6.15E-23	7.68E-06
Prolactin signaling pathway	hsa04917	403.4317	0.0100	3.47E-23	1.94E-07	0.3495	1.17E-22	7.68E-06
p53 signaling pathway	hsa04115	275.2800	0.0270	1.08E-26	4.97E-07	0.4235	6.16E-26	8.45E-06
Platelet activation	hsa04611	338.3987	0.0295	4.04E-18	5.41E-07	0.4296	8.71E-18	8.49E-06
Cell cycle	hsa04110	12566.8436	0.0365	5.87E-30	6.61E-07	0.4651	5.92E-29	8.99E-06
Vascular smooth muscle contraction	hsa04270	245.2489	0.0880	1.42E-14	1.52E-06	0.6351	2.46E-14	1.19E-05
AGE-RAGE signaling pathway in diabetic complications	hsa04933	302.4132	0.1324	4.59E-28	2.23E-06	0.6351	3.18E-27	1.24E-05
Insulin resistance	hsa04931	183.9960	0.2284	5.67E-23	3.72E-06	0.7359	1.83E-22	1.37E-05
PI3K-Akt signaling pathway	hsa04151	356.9245	0.4468	9.91E-40	6.98E-06	0.9024	5.50E-38	1.58E-05
Lipid and atherosclerosis	hsa05417	421.1504	0.4943	3.07E-28	7.67E-06	0.9620	2.20E-27	1.69E-05
MicroRNAs in cancer	hsa05206	252.9818	0.7571	4.29E-42	1.14E-05	0.9866	4.77E-40	1.71E-05
Type II diabetes mellitus	hsa04930	208.8111	0.6967	2.82E-14	1.06E-05	0.9866	4.74E-14	1.71E-05

ESM Table 20. Potential microRNA biomarkers enriched in T2D pathway and their priority verification order

Priority	miRNA	Direction	Presented in following meta-analyses
1	miR-29a-3p	1	overall, blood, serum, sensi-25, sensi-50
2	miR-221-3p	1	serum, sensi-50
3	miR-126-3p	-1	plasma, sensi-25
3	miR-26a-5p	-1	plasma, sensi-25
4	miR-503-5p	-1	blood, serum
5	miR-100-5p	-1	sensi-25, sensi-50
6	miR-101-3p	1	blood
6	miR-103a-3p	1	whole blood
6	miR-122-5p	1	whole blood
6	miR-199a-3p	1	plasma
6	miR-30b-5p	1	plasma
7	miR-130a-3p	-1	sensi-25
7	miR-143-3p	1	muscle
7	miR-145-5p	1	adipose
7	miR-19a-3p	1	sensi-25
7	miR-331-3p	-1	overall

"1" indicates up-regulation and "-1" indicates down-regulation; "sensi-25" and "sensi-50" indicates sensitivity analysis with sample size no less of 25 and 50, respectively.

The priority is according to the following order of importance (a) detectable in blood or blood fractions; (b) statistically significance in different analyses.

ESM Table 21. Contradictory findings on overall effect of human microRNAs between previous and present meta-analysis due to newly included studies

microRNA	Meta 2015			Meta 2022		
	Significant*	Up	Down	Significant*	Up	Down
miR-1-3p	No	0	0	Yes	0	4
miR-103a-3p	Yes	2	0	No	8	4
miR-103b	No	0	0	Yes	0	3
miR-106b-5p	Yes	2	0	No	3	4
miR-107	Yes	2	0	No	2	2
miR-10b-5p	No	0	1	Yes	0	3
miR-1228-3p	No	0	0	Yes	0	3
miR-1246	No	0	0	Yes	0	3
miR-1275	No	0	0	Yes	0	2
miR-1281	No	0	0	Yes	0	3
miR-1303	No	0	0	Yes	2	0
miR-133a-3p	No	0	1	Yes	0	2
miR-136-5p	No	0	0	Yes	2	0
miR-140-3p	No	0	0	Yes	3	0
miR-140-5p	Yes	2	0	No	2	2
miR-142-3p	Yes	4	0	No	4	4
miR-142-5p	No	1	0	Yes	2	0
miR-144-3p	Yes	3	0	No	12	2
miR-148a-3p	No	0	0	Yes	4	0
miR-152-3p	No	0	1	Yes	0	3
miR-155-5p	Yes	0	2	No	7	11
miR-16-5p	Yes	4	0	No	12	5
miR-181a-2-3p	No	1	0	Yes	2	0
miR-18a-5p	Yes	2	0	No	5	2
miR-18b-5p	No	0	0	Yes	0	2
miR-182-5p	No	0	1	Yes	0	3
miR-185-5p	Yes	2	0	No	2	3
miR-1973	No	0	0	Yes	0	2
miR-198	No	0	0	Yes	0	2
miR-19b-3p	No	1	0	Yes	3	0
miR-200b-3p	No	0	0	Yes	0	2
miR-208a	No	0	0	Yes	4	0
miR-20b-5p	Yes	0	2	No	3	5
miR-221-3p	Yes	3	0	No	9	2
miR-296-5p	No	1	0	Yes	2	0
miR-29c-3p	No	1	0	Yes	7	1
miR-302a-3p	No	1	0	Yes	4	0
miR-302b-3p	No	0	0	Yes	2	0
miR-30a-3p	No	0	1	Yes	0	2
miR-30c-5p	No	0	1	Yes	0	6
miR-30e-5p	Yes	0	3	No	1	7
miR-32-5p	No	0	0	Yes	2	0
miR-342-3p	Yes	0	2	No	2	4
miR-346	No	0	0	Yes	3	0
miR-34a-5p	Yes	2	0	No	11	2
miR-378	No	0	0	Yes	0	4
miR-409-3p	No	0	0	Yes	0	2
miR-423-3p	No	0	1	Yes	0	4
miR-4463	No	0	0	Yes	5	0
miR-4477a	No	0	0	Yes	0	2

miR-455-3p	No	0	0	Yes	2	0
miR-4695-5p	No	0	0	Yes	2	0
miR-4714-3p	No	0	0	Yes	0	2
miR-483-3p	No	0	0	Yes	2	0
miR-548o-3p	No	0	0	Yes	2	0
miR-571	No	0	0	Yes	2	0
miR-589-3p	No	0	0	Yes	2	0
miR-593	No	0	0	Yes	0	4
miR-660-5p	No	0	1	Yes	0	3
miR-661	No	0	0	Yes	2	0
miR-665	Yes	2	0	No	2	1
miR-766-3p	No	0	0	Yes	0	3
miR-770-5p	No	0	0	Yes	2	0
miR-892b	No	0	0	Yes	2	0
miR-9-5p	No	1	0	Yes	4	0

*indicates whether the microRNA was statistical significant in corresponding meta-analysis and number under Up and Down indicates the number of sub-studies reported the corresponding microRNA up-regulated and down-regulated, respectively.

ESM Table 22. Statistically significant dysregulation of miRNAs in humans by empirical Bayes estimation (n = 60)

	miRNA	No. of sub-studies	No. of samples	Overall effect		
				LogOR	[95%CI]	Adjusted p values
Up-regulation	miR-29a-3p	8	352	6.838	[5.4211, 8.2548]	2.77E-19
	miR-375	4	307	7.8447	[5.7837, 9.9057]	7.69E-12
	miR-589-3p	2	889	10.5404	[7.7535, 13.3273]	1.10E-11
	miR-4463	5	138	6.6571	[4.8722, 8.4420]	2.38E-11
	miR-346	3	180	8.2217	[5.9401, 10.5034]	1.45E-10
	miR-9-5p	4	152	6.8044	[4.8003, 8.8084]	2.53E-09
	miR-1303	2	246	9.3691	[6.5826, 12.1555]	3.92E-09
	miR-571	2	246	9.3691	[6.5826, 12.1555]	3.92E-09
	miR-661	2	246	9.3691	[6.5826, 12.1555]	3.92E-09
	miR-770-5p	2	246	9.3691	[6.5826, 12.1555]	3.92E-09
	miR-892b	2	246	9.3691	[6.5826, 12.1555]	3.92E-09
	miR-302a-3p	4	124	6.4617	[4.4525, 8.4709]	2.59E-08
	miR-208a	4	70	5.769	[3.7527, 7.7854]	1.82E-06
	miR-181a-2-3p	2	116	7.77	[4.9635, 10.5764]	5.12E-06
	miR-376a-3p	2	69	7.1099	[4.2982, 9.9217]	6.41E-05
	miR-142-5p	2	110	7.5032	[4.3913, 10.6150]	2.04E-04
	miR-140-3p	3	48	5.576	[3.2412, 7.9109]	2.55E-04
	miR-19b-3p	3	49	5.5337	[3.1943, 7.8730]	3.16E-04
	miR-148a-3p	4	913	7.7659	[4.4635, 11.0683]	3.60E-04
	miR-32-5p	2	45	6.2739	[3.4413, 9.1064]	1.26E-03
	miR-187-3p	2	40	6.0845	[3.2472, 8.9218]	2.34E-03
	miR-589	2	107	7.3325	[3.8801, 10.7850]	2.80E-03
	miR-136-5p	2	37	5.7482	[2.8907, 8.6058]	7.17E-03
	miR-4695-5p	2	32	5.6095	[2.7527, 8.4663]	1.06E-02
	miR-29c-3p	8	178	4.8661	[2.3085, 7.4237]	1.71E-02
	miR-296-5p	2	29	5.4174	[2.5522, 8.2826]	1.88E-02
	miR-302b-3p	2	28	5.3521	[2.4830, 8.2212]	2.28E-02
	miR-483-3p	2	26	5.2619	[2.3911, 8.1327]	2.91E-02
	miR-455-3p	2	28	5.2417	[2.3620, 8.1214]	3.20E-02
	miR-548o-3p	2	30	5.2248	[2.3322, 8.1173]	3.56E-02
	miR-320a	14	867	5.2885	[2.2827, 8.2899]	4.92E-02
Down-regulation	miR-30c-5p	6	305	7.4205	[5.7924, 9.0485]	3.68E-17
	miR-1-3p	4	347	8.9444	[6.9732, 10.9155]	5.27E-17
	miR-198	2	850	12.1054	[9.3304, 14.8805]	1.09E-15
	miR-593	4	200	7.8637	[5.8846, 9.8427]	6.08E-13
	miR-103b	3	269	9.0039	[6.7282, 11.2797]	7.89E-13
	miR-152-3p	3	192	8.2663	[5.9844, 10.5483]	1.11E-10
	miR-10b-5p	3	150	7.4974	[5.2028, 9.7920]	1.34E-08
	miR-423-3p	4	149	6.5335	[4.4917, 8.5753]	3.18E-08
	miR-133a-3p	2	169	8.8885	[6.1004,11.6766]	3.69E-08
	miR-374b-5p	3	134	7.1157	[4.8121, 9.4194]	1.25E-07
	miR-200b-3p	2	242	8.5849	[5.7880, 11.3818]	1.59E-07
	miR-4477a	2	133	8.3626	[5.5690, 11.1563]	3.95E-07
	miR-4714-3p	2	133	8.3626	[5.5690, 11.1563]	3.95E-07
	miR-378	4	64	5.6664	[3.6496, 7.6832]	3.25E-06
	miR-126a	2	93	7.5237	[4.7159, 10.3316]	1.34E-05
	miR-182-5p	3	56	5.8221	[3.4929, 8.1512]	8.56E-05
	miR-1228-3p	3	37	5.0521	[2.6954, 7.4087]	2.36E-03
	miR-766-3p	3	35	4.9924	[2.6343, 7.3505]	2.96E-03
	miR-652-3p	2	111	7.3736	[3.8398, 10.9074]	3.84E-03
	miR-1281	3	34	4.9235	[2.5616, 7.2853]	3.91E-03
	miR-660-5p	3	35	4.9138	[2.5467, 7.2809]	4.21E-03
	miR-409-3p	2	37	5.8618	[3.0131, 8.7104]	4.90E-03

	miR-1246	3	30	4.7846	[2.4197, 7.1495]	6.52E-03
	miR-1275	2	30	5.44	[2.5724, 8.3076]	1.78E-02
	miR-1973	2	30	5.44	[2.5724, 8.3076]	1.78E-02
	miR-199b-5p	2	104	7.1309	[3.2754, 10.9865]	2.57E-02
	miR-331-3p	2	104	7.1309	[3.2754, 10.9865]	2.57E-02
	miR-18b-5p	2	26	5.1858	[2.3077, 8.0638]	3.68E-02
	miR-30a-3p	2	25	5.1408	[2.2614, 8.0203]	4.16E-02

ESM Table 23. Statistically significant dysregulation of miRNAs in human pancreas by empirical Bayes estimation (n=1)

	miRNA	No. of sub-studies	No. of samples	Overall effect		
				LogOR	[95%CI]	Adjusted *p* values
Up-regulation	miR-187-3p	2	40	6.0845	[3.2472-8.9218]	2.63E-05

ESM Table 24. Statistically significant dysregulation of miRNAs in human heart by empirical Bayes estimation (n=2)

	miRNA	No. of sub-studies	No. of samples	Overall effect		
				LogOR	[95%CI]	Adjusted *p* values
Up-regulation	miR-208a	4	70	5.7690	[3.7527, 7.7854]	4.10E-08
Down-regulation	miR-15b-5p	3	42	5.3777	[3.0372, 7.7181]	1.34E-05

ESM Table 25. Statistically significant dysregulation of miRNAs in kidney by empirical Bayes estimation (n=1)

	miRNA	No. of sub-studies	No. of samples	Overall effect		
				LogOR	[95%CI]	Adjusted *p* values
Up-regulation	miR-1228-3p	3	37	5.0521	[2.6954, 7.4087]	1.09E-03

ESM Table 26. Statistically significant dysregulation of miRNAs in adipose by empirical Bayes estimation (n=6)

	miRNA	No. of sub-studies	No. of samples	Overall effect		
				LogOR	[95%CI]	Adjusted *p* values
Up-regulation	miR-125b-5p	2	85	7.051	[4.0961, 10.0059]	1.75E-05
	miR-140-3p	2	24	5.1299	[2.2535, 8.0063]	2.84E-03
	miR-145-5p	2	24	5.1299	[2.2535, 8.0063]	2.84E-03
	miR-146a-5p	2	24	5.1299	[2.2535, 8.0063]	2.84E-03
	miR-16-5p	2	24	5.1299	[2.2535, 8.0063]	2.84E-03
	miR-24-3p	2	24	5.1299	[2.2535, 8.0063]	2.84E-03

ESM Table 27. Statistically significant dysregulation of miRNAs in human muscle by empirical Bayes estimation (n=5)

	miRNA	No. of sub-studies	No. of samples	Overall effect		
				LogOR	[95%CI]	Adjusted *p* values
Up- regulation	miR-143-3p	2	112	7.6017	[4.6859-10.5174]	1.61E-06
Down-regulation	miR-10b-5p	2	114	7.6901	[4.8813-10.4989]	4.02E-07
	miR-10a-5p	2	108	7.3939	[4.0640-10.7237]	6.75E-05
	miR-27b-3p	2	108	7.3939	[4.0640-10.7237]	6.75E-05
	miR-363-3p	2	33	5.6293	[2.7711-8.4876]	5.65E-04

ESM Table 28. Statistically significant dysregulation of miRNAs in human blood by empirical Bayes estimation (n=50)

	miRNA	No. of sub-studies	No. of samples	Overall effect		
				LogOR	[95%CI]	Adjusted *p* values
Up-regulation	miR-29a-3p	4	272	7.8323	[5.8456, 9.8190]	6.71E-13
	miR-589-3p	2	889	10.5404	[7.7535, 13.3273]	7.56E-12
	miR-346	3	180	8.2217	[5.9401, 10.5034]	9.94E-11
	miR-9-5p	4	152	6.8044	[4.8003, 8.8084]	1.73E-09
	miR-770-5p	2	246	9.3691	[6.5826, 12.1555]	2.68E-09
	miR-661	2	246	9.3691	[6.5826, 12.1555]	2.68E-09
	miR-1303	2	246	9.3691	[6.5826, 12.1555]	2.68E-09
	miR-571	2	246	9.3691	[6.5826, 12.1555]	2.68E-09
	miR-892b	2	246	9.3691	[6.5826, 12.1555]	2.68E-09
	miR-4463	4	112	6.686	[4.6910, 8.6810]	3.10E-09
	miR-572	2	200	9.2302	[6.4447, 12.0157]	5.08E-09
	miR-128-3p	2	160	8.7889	[6.0000, 11.5778]	4.00E-08
	miR-302a-3p	3	112	6.8914	[4.5807, 9.2022]	3.09E-07
	miR-374a-5p	2	120	7.7535	[4.8930, 10.6139]	6.59E-06
	miR-375	3	252	7.836	[4.9199, 10.7521]	8.48E-06
	miR-376a-3p	2	69	7.1099	[4.2982, 9.9217]	4.39E-05
	miR-320c	3	49	5.6282	[3.2948, 7.9617]	1.38E-04
	miR-148a-3p	4	913	7.7659	[4.4635, 11.0683]	2.46E-04
	miR-193b-3p	2	49	6.4626	[3.6362, 9.2890]	4.52E-04
	miR-101-3p	2	45	6.2739	[3.4413, 9.1064]	8.66E-04
	miR-32-5p	2	45	6.2739	[3.4413, 9.1064]	8.66E-04
	miR-1260a	2	42	6.165	[3.3298, 9.0002]	1.24E-03
	miR-19b-3p	2	39	5.9061	[3.0575, 8.7546]	2.95E-03
	miR-136-5p	2	37	5.7482	[2.8907, 8.6058]	4.92E-03
	miR-302b-3p	2	28	5.3521	[2.4830, 8.2212]	1.56E-02
	miR-548o-3p	2	30	5.2248	[2.3322, 8.1173]	2.44E-02
	miR-335-5p	2	165	7.8129	[3.4098, 12.2160]	3.05E-02
	miR-132-3p	2	23	5.0466	[2.1656, 7.9277]	3.63E-02
	miR-135a-5p	2	90	6.8317	[2.9193, 10.7441]	3.78E-02
	miR-188-3p	2	26	5.0382	[2.1401, 7.9363]	4.00E-02
Down-regulation	miR-1-3p	4	347	8.9444	[6.9732, 10.9155]	3.61E-17
	miR-30c-5p	5	295	7.9181	[6.1442, 9.6919]	1.32E-16
	miR-198	2	850	12.1054	[9.3304, 14.8805]	7.50E-16
	miR-593	4	200	7.8637	[5.8846, 9.8427]	4.17E-13
	miR-103b	3	269	9.0039	[6.7282, 11.2797]	5.41E-13
	miR-197-3p	3	283	8.6733	[6.3905, 10.9560]	5.83E-12
	miR-200b-3p	2	242	8.5849	[5.7880, 11.3818]	1.09E-07
	miR-4477a	2	133	8.3626	[5.5690, 11.1563]	2.71E-07
	miR-4714-3p	2	133	8.3626	[5.5690, 11.1563]	2.71E-07
	miR-378	4	64	5.6664	[3.6496, 7.6832]	2.23E-06
	miR-152-3p	2	100	7.8637	[5.0648, 10.6625]	2.23E-06
	miR-126a	2	93	7.5237	[4.7159, 10.3316]	9.21E-06
	miR-503-5p	2	69	7.1099	[4.2982, 9.9217]	4.39E-05
	miR-182-5p	3	56	5.8221	[3.4929, 8.1512]	5.87E-05
	miR-423-3p	3	57	5.6437	[3.3040, 7.9835]	1.38E-04
	miR-374b-5p	2	42	6.1041	[3.2648, 8.9434]	1.53E-03
	miR-652-3p	2	111	7.3736	[3.8398, 10.9074]	2.64E-03
	miR-660-5p	3	35	4.9138	[2.5467, 7.2809]	2.89E-03
	miR-409-3p	2	37	5.8618	[3.0131, 8.7104]	3.36E-03
	miR-214-3p	2	89	6.9567	[3.4471, 10.4663]	6.22E-03

ESM Table 29. Sensitivity analysis based on human sample size by empirical Bayes estimation (n=90)

Sample size	Direction of regulation	miRNA	No. of sub-studies	Pooled sample size	Overall effect		
					LogOR	[95%CI]	Adjusted *p* values
No less than 25	Up- regulation	miR-93-5p	5	716	9.7747	[8.0145, 11.5348]	7.67E-26
		miR-210-3p	4	540	9.6747	[7.7066, 11.6429]	3.20E-20
		miR-29a-3p	4	295	8.2552	[6.2771, 10.2333]	1.60E-15
		miR-222-3p	4	203	7.7746	[5.7929, 9.7562]	8.29E-13
		miR-30d-5p	3	313	8.7249	[6.4439, 11.0060]	3.67E-12
		miR-148a-3p	2	889	10.5404	[7.7535, 13.3273]	6.94E-12
		miR-589-3p	2	889	10.5404	[7.7535, 13.3273]	6.94E-12
		miR-375	3	292	8.5832	[6.3000, 10.8664]	9.69E-12
		miR-346	3	180	8.2217	[5.9401, 10.5034]	9.13E-11
		miR-1303	2	246	9.3691	[6.5826, 12.1555]	2.46E-09
		miR-661	2	246	9.3691	[6.5826, 12.1555]	2.46E-09
		miR-770-5p	2	246	9.3691	[6.5826, 12.1555]	2.46E-09
		miR-892b	2	246	9.3691	[6.5826, 12.1555]	2.46E-09
		miR-571	2	246	9.3691	[6.5826, 12.1555]	2.46E-09
		miR-572	2	200	9.2302	[6.4447, 12.0157]	4.66E-09
		miR-9-5p	3	142	7.4373	[5.1390, 9.7355]	1.27E-08
		miR-4463	3	90	6.8025	[4.5007, 9.1044]	3.89E-07
		miR-146b-5p	2	221	8.8849	[5.8523, 11.9175]	5.23E-07
		miR-302a-3p	2	102	7.871	[5.0718, 10.6702]	1.99E-06
		miR-451a	2	119	7.8737	[5.0699, 10.6776]	2.08E-06
		miR-29c-3p	2	95	7.5697	[4.7632, 10.3762]	7.00E-06
		miR-376a-3p	2	69	7.1099	[4.2982, 9.9217]	4.03E-05
		miR-4306	2	64	6.9324	[4.1164, 9.7485]	7.84E-05
		miR-19a-3p	2	187	8.4249	[4.9854, 11.8644]	8.85E-05
	Down-regulation	miR-223-3p	5	343	8.16	[6.3904, 9.9296]	8.96E-18
		miR-1-3p	4	347	8.9444	[6.9732, 10.9155]	3.32E-17
		miR-197-3p	4	353	8.6362	[6.6601, 10.6123]	6.05E-16
		miR-198	2	850	12.1054	[9.3304, 14.8805]	6.89E-16
		miR-30c-5p	4	280	8.4854	[6.5111, 10.4596]	2.04E-15
		miR-144-5p	3	746	9.2328	[6.9538, 11.5119]	1.13E-13
		miR-593	4	200	7.8637	[5.8846, 9.8427]	3.82E-13

		miR-103b	3	269	9.0039	[6.7282, 11.2797]	4.97E-13
		miR-23a-3p	4	211	7.7607	[5.7785, 9.7428]	9.35E-13
		miR-26b-5p	4	176	7.54	[5.5565, 9.5234]	5.20E-12
		miR-152-3p	3	192	8.2663	[5.9844, 10.5483]	7.00E-11
		miR-320b	2	200	9.2302	[6.4447, 12.0157]	4.66E-09
		let-7f-5p	3	134	7.4077	[5.1138, 9.7017]	1.38E-08
		miR-130a-3p	3	116	7.3221	[5.0293, 9.6148]	2.17E-08
		miR-96-5p	3	133	7.3394	[5.0404, 9.6385]	2.20E-08
		miR-100-5p	2	175	8.8902	[6.1015, 11.6790]	2.32E-08
		miR-133a-3p	2	169	8.8885	[6.1004,11.6766]	2.32E-08
		miR-98-5p	2	169	8.8885	[6.1004, 11.6766]	2.32E-08
		miR-200b-3p	2	242	8.5849	[5.7880, 11.3818]	1.00E-07
		miR-4477a	2	133	8.3626	[5.5690, 11.1563]	2.49E-07
		miR-4714-3p	2	133	8.3626	[5.5690, 11.1563]	2.49E-07
		miR-423-3p	2	129	8.1601	[5.3621, 10.9580]	6.10E-07
		miR-10b-5p	2	128	8.097	[5.2971, 10.8969]	8.06E-07
		miR-342-3p	2	125	8.0642	[5.2646, 10.8637]	9.18E-07
		miR-374b-5p	2	119	7.8737	[5.0699, 10.6776]	2.08E-06
		miR-186-5p	2	88	7.6054	[4.8027, 10.4081]	5.88E-06
		miR-26a-5p	2	162	8.2401	[5.1650, 11.3151]	8.40E-06
		miR-126a	2	93	7.5237	[4.7159, 10.3316]	8.46E-06
		miR-126-3p	24	2748	5.8539	[2.9564, 8.7515]	4.20E-03
No less than 50	Up-regulation	miR-93-5p	5	716	9.7747	[8.0145, 11.5348]	5.34E-26
		miR-221-3p	5	781	9.3868	[7.6232, 11.1503]	6.90E-24
		miR-210-3p	4	540	9.6747	[7.7066, 11.6429]	2.23E-20
		miR-148a-3p	2	889	10.5404	[7.7535, 13.3273]	4.84E-12
		miR-589-3p	2	889	10.5404	[7.7535, 13.3273]	4.84E-12
		miR-27a-3p	3	218	8.5517	[6.2725, 10.8309]	7.49E-12
		miR-99b-5p	3	212	8.4823	[6.2024, 10.7622]	1.19E-11
		miR-346	3	180	8.2217	[5.9401, 10.5034]	6.36E-11
		miR-222-3p	3	175	8.1404	[5.8577, 10.4231]	1.08E-10
		miR-15b-5p	2	275	9.7437	[6.9607, 12.5267]	2.65E-10
		miR-30d-5p	2	276	9.4392	[6.6525, 12.2258]	1.23E-09
		miR-1303	2	246	9.3691	[6.5826, 12.1555]	1.72E-09
		miR-661	2	246	9.3691	[6.5826, 12.1555]	1.72E-09

	miR-770-5p	2	246	9.3691	[6.5826, 12.1555]	1.72E-09
	miR-892b	2	246	9.3691	[6.5826, 12.1555]	1.72E-09
	miR-571	2	246	9.3691	[6.5826, 12.1555]	1.72E-09
	miR-572	2	200	9.2302	[6.4447, 12.0157]	3.24E-09
	miR-375	2	255	9.2294	[6.4389, 12.0199]	3.52E-09
	miR-29a-3p	2	220	9.1972	[6.4098, 11.9846]	3.90E-09
	miR-302a-3p	2	102	7.871	[5.0718, 10.6702]	1.39E-06
	miR-138-5p	2	100	7.8637	[5.0648, 10.6625]	1.43E-06
Down-regulation	miR-1-3p	4	347	8.9444	[6.9732, 10.9155]	2.31E-17
	miR-198	2	850	12.1054	[9.3304, 14.8805]	4.80E-16
	miR-30c-5p	4	280	8.4854	[6.5111, 10.4596]	1.42E-15
	miR-197-3p	3	326	9.2808	[7.0061, 11.5554]	4.99E-14
	miR-144-5p	3	746	9.2328	[6.9538, 11.5119]	7.88E-14
	miR-593	4	200	7.8637	[5.8846, 9.8427]	2.66E-13
	miR-103b	3	269	9.0039	[6.7282, 11.2797]	3.46E-13
	miR-223-3p	3	262	8.6563	[6.3763, 10.9364]	3.90E-12
	miR-152-3p	3	192	8.2663	[5.9844, 10.5483]	4.88E-11
	miR-320b	2	200	9.2302	[6.4447, 12.0157]	3.24E-09
	miR-100-5p	2	175	8.8902	[6.1015, 11.6790]	1.62E-08
	miR-133a-3p	2	169	8.8885	[6.1004,11.6766]	1.62E-08
	miR-98-5p	2	169	8.8885	[6.1004, 11.6766]	1.62E-08
	miR-4477a	2	133	8.3626	[5.5690, 11.1563]	1.73E-07
	miR-4714-3p	2	133	8.3626	[5.5690, 11.1563]	1.73E-07
	miR-26b-5p	2	102	7.8932	[5.0946, 10.6918]	1.26E-06

ESM Table 30. Statistically significant dysregulation of miRNAs in PBMCs by empirical Bayes estimation (n = 12)

	miRNA	No. of sub-studies	No. of samples	Overall effect		
				LogOR	[95%CI]	Adjusted p values
Up-regulation	miR-18a-5p	2	413	10.6376	[7.8589, 13.4163]	7.48E-13
	miR-346	3	180	8.2217	[5.9401, 10.5034]	1.96E-11
	miR-29b-3p	2	36	5.8889	[3.0451, 8.7327]	5.93E-04
	miR-222-3p	2	36	5.8889	[3.0451, 8.7327]	5.93E-04
	miR-186-5p	2	36	5.8889	[3.0451, 8.7327]	5.93E-04
	miR-188-3p	2	26	5.0382	[2.1401, 7.9363]	7.87E-03
	miR-20a-5p	2	26	5.0382	[2.1401, 7.9363]	7.87E-03
Down-regulation	miR-146a-5p	3	140	7.6934	[5.4058, 9.9810]	5.22E-10
	miR-144-5p	2	133	8.3626	[5.5690, 11.1563]	5.33E-08
	miR-4477a	2	133	8.3626	[5.5690, 11.1563]	5.33E-08
	miR-4714-3p	2	133	8.3626	[5.5690, 11.1563]	5.33E-08
	miR-155-5p	2	80	7.4271	[4.6217, 10.2326]	2.54E-06

ESM Table 31. Statistically significant dysregulation of miRNAs in Whole blood by empirical Bayes estimation (n = 25)

	miRNA	No. of sub-studies	No. of samples	Overall effect		
				LogOR	[95%CI]	Adjusted p values
Up-regulation	miR-122-5p	2	889	10.5404	[7.7535, 13.3273]	3.10E-12
	miR-589-3p	2	889	10.5404	[7.7535, 13.3273]	3.10E-12
	miR-155-5p	3	120	7.4271	[5.1365, 9.7177]	5.20E-09
	miR-103a-3p	2	120	7.8123	[5.0062, 10.6185]	1.22E-06
	miR-15b-5p	2	48	6.4378	[3.6111, 9.2645]	2.01E-04
	miR-26b-5p	2	39	5.9944	[3.1524, 8.8363]	8.90E-04
	miR-151a-5p	2	39	5.9944	[3.1524, 8.8363]	8.90E-04
	miR-150-5p	2	111	7.3736	[3.8398, 10.9074]	1.08E-03
	miR-192-5p	2	111	7.3736	[3.8398, 10.9074]	1.08E-03
	miR-27a-3p	2	111	7.3736	[3.8398, 10.9074]	1.08E-03
	miR-320a	2	111	7.3736	[3.8398, 10.9074]	1.08E-03
	miR-148a-3p	3	895	8.3764	[4.0322, 12.7206]	3.93E-03
	miR-548o-3p	2	30	5.2248	[2.3322, 8.1173]	1.00E-02
	miR-151a-3p	2	21	4.7465	[1.8376, 7.6554]	3.45E-02
Down-regulation	miR-24-3p	4	211	7.6194	[5.6304, 9.6085]	1.50E-12
	miR-378	4	64	5.6664	[3.6496, 7.6832]	9.13E-07
	miR-486-5p	2	48	6.4378	[3.6111, 9.2645]	2.01E-04
	miR-342-3p	2	48	6.305	[3.4702, 9.1397]	3.25E-04
	miR-17-5p	2	111	7.3736	[3.8398, 10.9074]	1.08E-03
	miR-652-3p	2	111	7.3736	[3.8398, 10.9074]	1.08E-03
	miR-210-3p	2	32	5.6664	[2.8143, 8.5186]	2.47E-03
	let-7f-5p	2	89	6.9567	[3.4471, 10.4663]	2.55E-03
	miR-214-3p	2	89	6.9567	[3.4471, 10.4663]	2.55E-03
	miR-20a-5p	2	21	4.7465	[1.8376, 7.6554]	3.45E-02
	miR-660-5p	2	21	4.7465	[1.8376, 7.6554]	3.45E-02

ESM Table 32. Statistically significant dysregulation of miRNAs in serum by empirical Bayes estimation (n = 38)

	miRNA	No. of sub-studies	No. of samples	Overall effect		
				LogOR	[95%CI]	Adjusted p values
Up-regulation	miR-210-3p	4	540	9.6747	[7.7066, 11.6429]	2.29E-20
	miR-221-3p	6	793	8.701	[6.7983, 10.6037]	1.27E-17
	miR-29a-3p	3	257	8.5626	[6.2811, 10.8441]	7.60E-12
	miR-15a-5p	4	209	6.8546	[4.8481, 8.8610]	8.60E-10
	miR-125b-5p	2	246	9.3691	[6.5826, 12.1555]	1.76E-09
	miR-1303	2	246	9.3691	[6.5826, 12.1555]	1.76E-09
	miR-571	2	246	9.3691	[6.5826, 12.1555]	1.76E-09
	miR-661	2	246	9.3691	[6.5826, 12.1555]	1.76E-09
	miR-770-5p	2	246	9.3691	[6.5826, 12.1555]	1.76E-09
	miR-892b	2	246	9.3691	[6.5826, 12.1555]	1.76E-09
	miR-130b-3p	2	194	9.1696	[6.3837, 11.9555]	4.44E-09
	miR-99b-5p	4	162	6.6902	[4.6165, 8.7638]	1.02E-08
	miR-128-3p	2	160	8.7889	[6.0000, 11.5778]	2.62E-08
	miR-302a-3p	3	112	6.8914	[4.5807, 9.2022]	2.02E-07
	miR-9-5p	3	107	6.8083	[4.4964, 9.1202]	3.14E-07
	miR-27b-3p	3	85	6.1699	[3.8419, 8.4978]	8.20E-06
	miR-24-3p	4	200	6.5303	[4.0615, 8.9991]	8.68E-06
	miR-376a-3p	2	69	7.1099	[4.2982, 9.9217]	2.88E-05
	miR-124-3p	2	47	6.0729	[3.2224, 8.9235]	1.19E-03
	miR-194-5p	2	39	5.9049	[3.0577, 8.7521]	1.92E-03
	miR-150-5p	2	49	5.9913	[2.9747, 9.0079]	3.96E-03
	miR-20b-5p	2	49	5.9913	[2.9747, 9.0079]	3.96E-03
	miR-107	2	23	5.0466	[2.1656, 7.9277]	2.39E-02
	miR-132-3p	2	23	5.0466	[2.1656, 7.9277]	2.39E-02
	miR-1304-5p	2	20	4.7958	[1.9007, 7.6908]	4.67E-02
	miR-302d-3p	2	20	4.7958	[1.9007, 7.6908]	4.67E-02
Down-regulation	miR-198	2	850	12.1054	[9.3304, 14.8805]	4.92E-16
	miR-593	4	200	7.8637	[5.8846, 9.8427]	2.73E-13
	miR-1-3p	3	270	9.0217	[6.7461, 11.2973]	3.13E-13
	miR-192-5p	4	381	7.7444	[5.3454, 10.1433]	1.00E-08
	miR-152-3p	2	100	7.8637	[5.0648, 10.6625]	1.46E-06
	miR-186-5p	2	88	7.6054	[4.8027, 10.4081]	4.20E-06
	miR-503-5p	2	69	7.1099	[4.2982, 9.9217]	2.88E-05
	miR-423-3p	3	57	5.6437	[3.3040, 7.9835]	9.08E-05
	let-7i-5p	2	54	6.2461	[3.3984, 9.0939]	6.88E-04
	miR-1246	2	20	4.7958	[1.9007, 7.6908]	4.67E-02
	miR-6131	2	20	4.7958	[1.9007, 7.6908]	4.67E-02
	miR-98-5p	2	20	4.7958	[1.9007, 7.6908]	4.67E-02

ESM Table 33. Statistically significant dysregulation of miRNAs in plasma by empirical Bayes estimation (n = 25)

	miRNA	No. of sub-studies	No. of samples	Overall effect		
				LogOR	[95%CI]	Adjusted p values
Up-regulation	miR-144-3p	6	951	9.2373	[7.3972, 11.0775]	1.99E-21
	miR-93-5p	4	564	9.3044	[7.2507, 11.3582]	1.75E-17
	miR-34a-5p	5	183	7.0493	[5.2683, 8.8303]	2.25E-13
	miR-4463	4	112	6.686	[4.6910, 8.6810]	1.32E-09
	miR-572	2	200	9.2302	[6.4447, 12.0157]	2.16E-09
	miR-29c-3p	2	95	7.5697	[4.7632, 10.3762]	3.25E-06
	miR-30b-5p	2	172	8.1724	[4.4843, 11.8604]	3.64E-04
	miR-199a-3p	2	42	6.1802	[3.3460, 9.0144]	4.99E-04
	miR-320c	2	34	5.6717	[2.8136, 8.5298]	2.60E-03
Down-regulation	miR-30c-5p	4	280	8.4854	[6.5111, 10.4596]	9.46E-16
	miR-223-3p	4	309	8.4201	[6.4439, 10.3962]	1.76E-15
	miR-130b-3p	3	140	7.5897	[5.2989, 9.8804]	2.18E-09
	miR-200b-3p	2	242	8.5849	[5.7880, 11.3818]	4.65E-08
	miR-185-5p	2	125	8.2932	[5.4995, 11.0870]	1.55E-07
	miR-126-3p	14	1421	7.5404	[4.8793, 10.2016]	7.28E-07
	miR-26a-5p	2	162	8.2401	[5.1650, 11.3151]	3.90E-06
	miR-126a	2	93	7.5237	[4.7159, 10.3316]	3.93E-06
	miR-125b-5p	2	93	7.5237	[4.7159, 10.3316]	3.93E-06
	miR-26b-5p	2	78	7.2781	[4.4681, 10.0880]	9.98E-06
	miR-195-5p	2	85	7.2492	[4.4345, 10.0639]	1.16E-05
	miR-197-3p	2	187	8.4249	[4.9854, 11.8644]	4.11E-05
	miR-192-5p	2	79	6.8486	[3.7805, 9.9167]	3.15E-04
	miR-15b-5p	2	41	5.9617	[3.1116, 8.8118]	1.08E-03
	miR-182-5p	2	41	5.9617	[3.1116, 8.8118]	1.08E-03
	miR-20a-5p	2	30	5.4271	[2.5589, 8.2953]	5.41E-03

ESM Table 34. Statistically significant dysregulation of miRNAs detected by PCR-based methods and empirical Bayes estimation (n = 61)

	miRNA	No. of sub-studies	No. of samples	Overall effect		
				LogOR	[95%CI]	Adjusted p values
Up-regulation	miR-144-3p	12	1105	7.4907	[5.9419, 9.0395]	2.25E-19
	miR-29a-3p	8	352	6.838	[5.4211, 8.2548]	2.74E-19
	miR-29c-3p	7	172	6.1063	[4.5842, 7.6284]	3.30E-13
	miR-375	4	307	7.8447	[5.7837, 9.9057]	7.60E-12
	miR-589-3p	2	889	10.5404	[7.7535, 13.3273]	1.09E-11
	miR-4463	5	138	6.6571	[4.8722, 8.4420]	2.35E-11
	miR-346	3	180	8.2217	[5.9401, 10.5034]	1.43E-10
	miR-1303	2	246	9.3691	[6.5826, 12.1555]	3.87E-09
	miR-571	2	246	9.3691	[6.5826, 12.1555]	3.87E-09
	miR-661	2	246	9.3691	[6.5826, 12.1555]	3.87E-09
	miR-770-5p	2	246	9.3691	[6.5826, 12.1555]	3.87E-09
	miR-892b	2	246	9.3691	[6.5826, 12.1555]	3.87E-09
	miR-302a-3p	3	114	6.9902	[4.6842, 9.2962]	2.49E-07
	miR-9-5p	3	107	6.8083	[4.4964, 9.1202]	6.90E-07
	miR-148a-3p	3	907	9.0132	[5.9479, 12.0786]	7.27E-07
	miR-208a	4	70	5.769	[3.7527, 7.7854]	1.80E-06
	miR-376a-3p	2	69	7.1099	[4.2982, 9.9217]	6.34E-05
	miR-142-5p	2	110	7.5032	[4.3913, 10.6150]	2.02E-04
	miR-19b-3p	3	49	5.5337	[3.1943, 7.8730]	3.12E-04
	miR-30b-5p	3	182	7.0631	[3.9827, 10.1436]	6.15E-04
	miR-32-5p	2	45	6.2739	[3.4413, 9.1064]	1.25E-03
	miR-187-3p	2	40	6.0845	[3.2472, 8.9218]	2.31E-03
	miR-589	2	107	7.3325	[3.8801, 10.7850]	2.77E-03
	miR-136-5p	2	37	5.7482	[2.8907, 8.6058]	7.09E-03
	miR-4695-5p	2	32	5.6095	[2.7527, 8.4663]	1.05E-02
	miR-296-5p	2	29	5.4174	[2.5522, 8.2826]	1.85E-02
	miR-483-3p	2	26	5.2619	[2.3911, 8.1327]	2.88E-02
	miR-455-3p	2	28	5.2417	[2.3620, 8.1214]	3.17E-02
	miR-140-3p	2	24	5.1299	[2.2535, 8.0063]	4.16E-02
	miR-320a	14	867	5.2885	[2.2827, 8.2899]	4.87E-02
Down-regulation	miR-30c-5p	6	305	7.4205	[5.7924, 9.0485]	3.63E-17
	miR-1-3p	4	347	8.9444	[6.9732, 10.9155]	5.21E-17
	miR-198	2	850	12.1054	[9.3304, 14.8805]	1.08E-15
	miR-100-5p	6	239	6.7735	[5.1393, 8.4077]	3.98E-14
	miR-593	4	200	7.8637	[5.8846, 9.8427]	6.01E-13
	miR-103b	3	269	9.0039	[6.7282, 11.2797]	7.81E-13
	miR-152-3p	3	192	8.2663	[5.9844, 10.5483]	1.10E-10
	miR-10b-5p	3	150	7.4974	[5.2028, 9.7920]	1.33E-08
	miR-133a-3p	2	169	8.8885	[6.1004,11.6766]	3.65E-08
	miR-374b-5p	3	134	7.1157	[4.8121, 9.4194]	1.24E-07
	miR-200b-3p	2	242	8.5849	[5.7880, 11.3818]	1.58E-07
	miR-423-3p	3	139	7.0846	[4.6737, 9.4955]	7.42E-07
	miR-98-5p	3	179	7.5755	[4.9202, 10.2308]	1.98E-06
	miR-378	4	64	5.6664	[3.6496, 7.6832]	3.21E-06
	miR-126a	2	93	7.5237	[4.7159, 10.3316]	1.33E-05
	miR-363-3p	4	55	5.2327	[3.1969, 7.2685]	4.14E-05
	miR-182-5p	3	56	5.8221	[3.4929, 8.1512]	8.47E-05
	miR-30e-3p	2	49	6.4626	[3.6362, 9.2890]	6.52E-04
	miR-1228-3p	3	37	5.0521	[2.6954, 7.4087]	2.33E-03
	miR-766-3p	3	35	4.9924	[2.6343, 7.3505]	2.93E-03
	miR-652-3p	2	111	7.3736	[3.8398, 10.9074]	3.80E-03
	miR-1281	3	34	4.9235	[2.5616, 7.2853]	3.86E-03

	miR-409-3p	2	37	5.8618	[3.0131, 8.7104]	4.85E-03
	miR-1275	2	30	5.44	[2.5724, 8.3076]	1.76E-02
	miR-1973	2	30	5.44	[2.5724, 8.3076]	1.76E-02
	miR-660-5p	2	29	5.3927	[2.5242, 8.2612]	2.02E-02
	miR-199b-5p	2	104	7.1309	[3.2754, 10.9865]	2.54E-02
	miR-331-3p	2	104	7.1309	[3.2754, 10.9865]	2.54E-02
	miR-223-3p	8	392	5.9464	[2.7181, 9.1748]	2.69E-02
	miR-18b-5p	2	26	5.1858	[2.3077, 8.0638]	3.63E-02
	miR-30a-3p	2	25	5.1408	[2.2614, 8.0203]	4.11E-02

ESM Table 35. Statistically significant dysregulation of miRNAs detected by RNA-Seq and empirical Bayes estimation (n=11)

	miRNA	No. of sub-studies	No. of samples	Overall effect		
				LogOR	[95%CI]	Adjusted *p* values
Up-regulation	miR-15b-5p	2	48	6.4378	[3.6111, 9.2645]	8.86E-05
	miR-99b-5p	2	34	5.6364	[2.7761, 8.4967]	1.23E-03
	miR-548o-3p	2	30	5.2248	[2.3322, 8.1173]	4.40E-03
Down-regulation	miR-144-3p	2	133	8.3626	[5.5690, 11.1563]	4.88E-08
	miR-144-5p	2	133	8.3626	[5.5690, 11.1563]	4.88E-08
	miR-4477a	2	133	8.3626	[5.5690, 11.1563]	4.88E-08
	miR-4714-3p	2	133	8.3626	[5.5690, 11.1563]	4.88E-08
	miR-486-5p	3	58	5.9077	[3.5816, 8.2339]	7.07E-06
	miR-126-5p	2	16	4.3543	[1.4258, 7.2828]	3.93E-02
	miR-16-5p	2	16	4.3543	[1.4258, 7.2828]	3.93E-02
	miR-30b-5p	2	16	4.3543	[1.4258, 7.2828]	3.93E-02

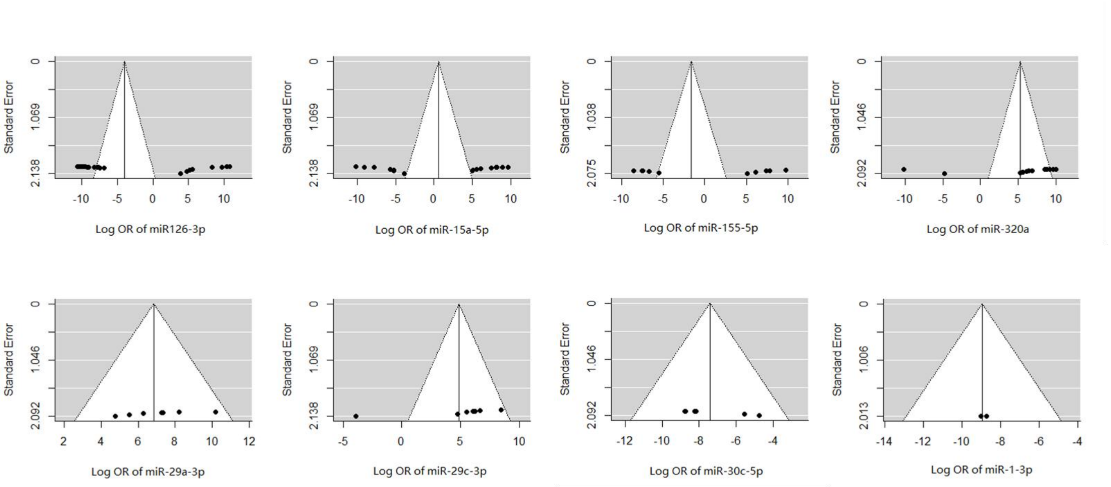

ESM Fig. 1. Funnel plots of publication bias on different microRNAs

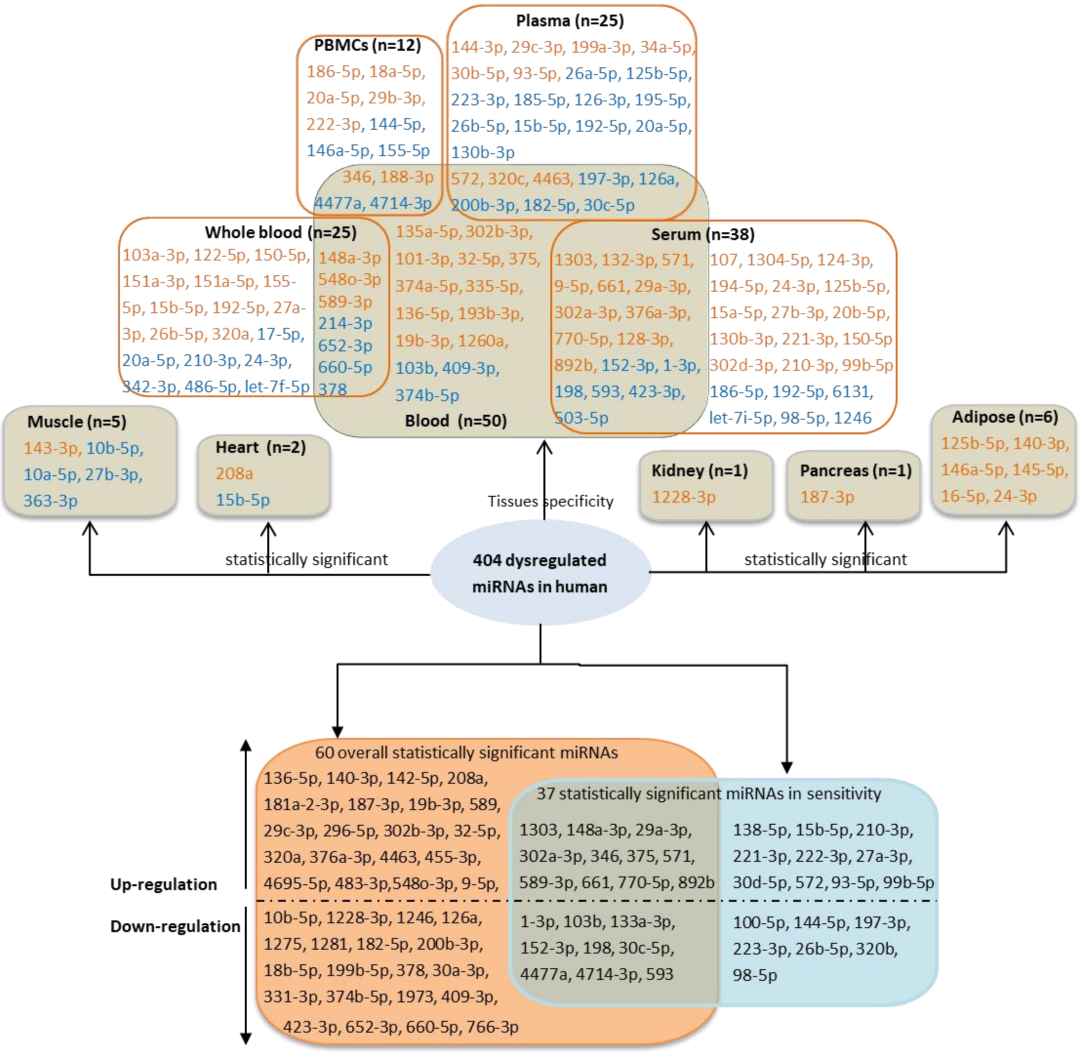

ESM Fig. 2. The Venn diagram of microRNA categories and flow chart of systematic review. “n”, number of statistically significant microRNAs. The prefix “miR-” of microRNA identifiers is omited. microRNAs in red are up-regulated and in blue are down-regulated.

Appendix

References of included studies

1 Al-Hayali MA, Sozer V, Durmus S, et al. Clinical value of circulating microribonucleic acids miR-1 and miR-21 in evaluating the diagnosis of acute heart failure in asymptomatic type 2 diabetic patients. Biomolecules 2019;9. doi:10.3390/biom9050193

2 Alicka M, Major P, Wysocki M, et al. Adipose-derived mesenchymal stem cells isolated from patients with type 2 diabetes show reduced "stemness" through an altered secretome profile, impaired anti-oxidative protection, and mitochondrial dynamics deterioration. J Clin Med 2019;8:765. doi:10.3390/jcm8060765

3 Alipoor B, Ghaedi H, Meshkani R, et al. The rs2910164 variant is associated with reduced miR-146a expression but not cytokine levels in patients with type 2 diabetes. J Endocrinol Invest Published Online First: 2017. doi:10.1007/s40618-017-0766-z

4 Al-Kafaji G, Al-Mahroos G, Alsayed NA, et al. Peripheral blood microRNA-15a is a potential biomarker for type 2 diabetes mellitus and pre-diabetes. Mol Med Rep 2015;12:7485–90. doi:10.3892/mmr.2015.4416

5 Al-Kafaji G, Al-Mahroos G, Al-Muhtaresh HA, et al. Decreased expression of circulating microRNA-126 in patients with type 2 diabetic nephropathy : A potential blood-based biomarker. Exp Ther Med 2016;12:815–22. doi:10.3892/etm.2016.3395

6 Al-kafaji G, Al-mahroos G, Al-muhtaresh HA, et al. Circulating endothelium-enriched microRNA-126 as a potential biomarker for coronary artery disease in type 2 diabetes mellitus patients. Biomarkers 2017;22:268–78. doi:10.1080/1354750X.2016.1204004

7 Avgeris M, Kokkinopoulou I, Maratou E, et al. Blood-based analysis of 84 microRNAs identifies molecules deregulated in individuals with type-2 diabetes, risk factors for the disease or metabolic syndrome. Diabetes Res Clin Pract 2020;164. doi:10.1016/j.diabres.2020.108187

8 Bai L, Li J, Panagal M, et al. Methylation dependent microRNA 1285-5p and sterol carrier proteins 2 in type 2 diabetes mellitus. Artif Cells, Nanomedicine Biotechnol 2019;47:3417–22. doi:10.1080/21691401.2019.1652625

9 Bai X, Geng J, Zhou Z, et al. MicroRNA-130b improves renal tubulointerstitial fibrosis via repression of Snail-induced epithelial-mesenchymal transition in diabetic nephropathy. Sci Rep 2016;6:20475. doi:10.1038/srep20475

10 Balasubramanyam M, Aravind S, Gokulakrishnan K, et al. Impaired miR-146a expression links subclinical inflammation and insulin resistance in type 2 diabetes. Mol Cell Biochem 2011;351:197–205. doi:10.1007/s11010-011-0727-3

11 Baldeón RL, Weigelt K, Wit H De, et al. Type 2 diabetes monocyte microRNA and mRNA expression : dyslipidemia associates with increased differentiation-related genes but not inflammatory activation. PLoS One 2015;10:1–19. doi:10.1371/journal.pone.0129421

12 Baltaci OF, Çolakoglu S, Gullu Amuran G, et al. Exploring the role of miRNAs in the diagnosis of MODY3. Turkish J Med Sci 2018;48:620–7. doi:10.3906/sag-1711-98

13 Bao L, Fu X, Si M, et al. MicroRNA-185 targets SOCS3 to inhibit beta-cell dysfunction in diabetes. PLoS One 2015;10:1–14. doi:10.1371/journal.pone.0116067

14 Blum A, Meerson A, Rohana H, et al. MicroRNA-423 may regulate diabetic vasculopathy. Clin Exp Med 2019;19:469–77. doi:10.1007/s10238-019-00573-8

15 Bork-jensen J, Scheele C, Christophersen D, et al. Glucose tolerance is associated with differential expression of microRNAs in skeletal muscle: results from studies of twins with and without type 2 diabetes. Diabetologia 2015;58:363–73. doi:10.1007/s00125-014-3434-2

16 Brovkina O, Nikitin A, Khodyrev D, et al. Role of microRNAs in the regulation of subcutaneous white adipose tissue in individuals with obesity and without type 2 diabetes. Front Endocrinol (Lausanne) 2019;10:1–9. doi:10.3389/fendo.2019.00840

17 Candia P De, Spinetti G, Specchia C, et al. A unique plasma microRNA profile defines type 2 diabetes progression. PLoS One 2017;12:1–13. doi:10.1371/journal.pone.0188980

18 Cheng S, Cui Y, Fan L, et al. T2DM inhibition of endothelial miR-342-3p facilitates angiogenic dysfunction via repression of FGF11 signaling. Biochem Biophys Res Commun 2018;503:71–8. doi:10.1016/j.bbrc.2018.05.179

19 Cheng Y, Wang D, Wang F, et al. Endogenous miR-204 protects the kidney against chronic injury in hypertension and diabetes. J Am Soc Nephrol 2020;31:1539–54. doi:10.1681/ASN.2019101100

20 Conserva F, Barozzino M, Pesce F, et al. Urinary miRNA-27b-3p and miRNA-1228-3p correlate with the progression of kidney fibrosis in diabetic nephropathy. Sci Rep 2019;9:1–11. doi:10.1038/s41598-019-47778-1

21 Corral-Fernández NE, Salgado-Bustamante M, Martínez-Leija ME, et al. Dysregulated miR-155 expression in peripheral blood mononuclear cells from patients with type 2 diabetes. Exp Clin Endocrinol Diabetes 2013;121:347–53. doi:10.1055/s-0033-1341516

22 Cui X, You L, Zhu L, et al. Change in circulating microRNA profile of obese children indicates future risk of adult diabetes. Metabolism Published Online First: 2017. doi:10.1016/j.metabol.2017.09.006

23 Dahlmans D, Houzelle A, Andreux P, et al. An unbiased silencing screen in muscle cells miR-34c as regulators of skeletal muscle mitochondrial metabolism. Mol Metab 2017;6:1429–42. doi:10.1016/j.molmet.2017.08.007

24 Dai X, Pang W, Zhou Y, et al. Altered profile of serum microRNAs in pancreatic cancer-associated new-onset diabetes mellitus. J Diabetes 2016;8:422–33. doi:10.1111/1753-0407.12313

25 Dangwal S, Stratmann B, Bang C, et al. Impairment of wound healing in patients with type 2 diabetes mellitus influences circulating microRNA patterns via inflammatory cytokines. Arter Thromb Vasc Biol 2015;35:1480–8. doi:10.1161/ATVBAHA.114.305048

26 Deng X, Liu Y, Luo M, et al. Circulating miRNA-24 and its target YKL-40 as potential biomarkers in patients with coronary heart disease and type 2 diabetes mellitus. Oncotarget 2017;1:63038–46. doi:10.18632/oncotarget.18593

27 El Samaloty NM, Hassan ZA, Hefny ZM, et al. Circulating microRNA-155 is associated with insulin resistance in chronic hepatitis C patients. Arab J Gastroenterol 2019;20:1–7. doi:10.1016/j.ajg.2019.01.011

28 Elemeery MN, Mohamed MA, Madkour MA, et al. MicroRNA signature in patients with hepatocellular carcinoma associated with type 2 diabetes. World J Gastroenterol 2019;25:6322–41. doi:10.3748/wjg.v25.i42.6322

29 Fejes Z, Póliska S, Czimmerer Z, et al. Hyperglycaemia suppresses microRNA expression in platelets to increase P2RY12 and SELP levels in type 2 diabetes mellitus. Thromb Haemost 2017;117:529–42.

30 Fomison-nurse I, Saw EEL, Gandhi S, et al. Diabetes induces the activation of pro-ageing miR-34a in the heart , but has differential effects on cardiomyocytes and cardiac progenitor cells. Cell Death Differ 2018;25:1336–49.

doi:10.1038/s41418-017-0047-6
31 Fulzele S, El-sherbini A, Ahmad S, et al. MicroRNA-146b-3p regulates retinal inflammation by suppressing adenosine deaminase-2 in diabetes. Biomed Res Int 2015;2015:1–8. doi:10.1155/2015/846501
32 Gallagher IJ, Scheele C, Keller P, et al. Integration of microRNA changes in vivo identifies novel molecular features of muscle insulin resistance in type 2 diabetes. Genome Med 2010;2:9. doi:10.1186/gm130
33 Giannella A, Radu CM, Franco L, et al. Circulating levels and characterization of microparticles in patients with different degrees of glucose tolerance. Cardiovasc Diabetol 2017;16:118. doi:10.1186/s12933-017-0600-0
34 Guglielmi V, D'Adamo M, Menghini R, et al. MicroRNA 21 is up-regulated in adipose tissue of obese diabetic subjects. Nutr Heal Aging 2017;4:141–5. doi:10.3233/NHA-160020
35 Guo J, Li J, Zhao J, et al. MiRNA-29c regulates the expression of inflammatory cytokines in diabetic nephropathy by targeting tristetraprolin. Sci Rep 2017;7:1–13. doi:10.1038/s41598-017-01027-5
36 Han YL, Cao XE, Wang JX, et al. Correlations of microRNA-124a and microRNA-30d with clinicopathological features of breast cancer patients with type 2 diabetes mellitus. Springerplus 2016;5:2107. doi:10.1186/s40064-016-3786-9
37 He X, Zheng Y, Liu S, et al. Altered plasma microRNAs as novel biomarkers for arteriosclerosis obliterans. J Atheroscler Thromb 2016;23:196–206.
38 He X, Du C, Yan Z, et al. Downregulation of microRNA-4463 attenuates high-glucose- and hypoxia- induced endothelial cell injury by targeting PNUTS. Cell Physiol Biochem 2018;49:2073–87. doi:10.1159/000493717
39 Henriksen TI, Davidsen PK, Pedersen M, et al. Dysregulation of a novel miR-23b/27b-p53 axis impairs muscle stem cell differentiation of humans with type 2 diabetes. Mol Metab 2017;6:770–9. doi:10.1016/j.molmet.2017.04.006
40 Hou X, Wu W, Yin B, et al. MicroRNA-463-3p/ABCG4 : a new axis in glucose-stimulated insulin secretion. Obesity 2016;24:2368–76. doi:10.1002/oby.21655
41 Hou X, Tian J, Geng J, et al. MicroRNA-27a promotes renal tubulointerstitial fibrosis via suppressing PPARγ pathway in diabetic nephropathy. Oncotarget 2016;7:47760.
42 Jansen F, Wang H, Przybilla D, et al. Vascular endothelial microparticles-incorporated microRNAs are altered in patients with diabetes mellitus. Cardiovasc Diabetol 2016;15:1–10. doi:10.1186/s12933-016-0367-8
43 Jiang Q, Lyu X, Yuan Y, et al. Plasma miR-21 expression: an indicator for the severity of type 2 diabetes with diabetic retinopathy. Biosci Rep 2017;37:1–10. doi:10.1042/BSR20160589
44 Jiang X, Luo Y, Zhao S, et al. Clinical significance and expression of microRNA in diabetic patients with erectile dysfunction. Exp Ther Med 2015;10:213–8. doi:10.3892/etm.2015.2443
45 Jiao Y, Zhu M, Mao X, et al. MicroRNA-130a expression is decreased in Xinjiang Uygur patients with type 2 diabetes mellitus. Am J Transl Res 2015;7:1984–91.
46 Jones A, Danielson KM, Benton MC, et al. MiRNA signatures of insulin resistance in obesity. Obesity 2017;25:1734–44. doi:10.1002/oby.21950
47 Kamalden TA, Macgregor-Das AM, Kannan SM, et al. Exosomal microRNA-15a transfer from the pancreas augments diabetic complications by inducing oxidative stress. Antioxid Redox Signal 2017;March:1–18. doi:10.1089/ars.2016.6844
48 Karolina DS, Armugam A, Tavintharan S, et al. MicroRNA 144 impairs insulin signaling by inhibiting the expression of insulin receptor substrate 1 in type 2 diabetes mellitus. PLoS One 2011;6:e22839. doi:10.1371/journal.pone.0022839
49 Karolina DS, Tavintharan S, Armugam A, et al. Circulating miRNA profiles in patients with metabolic syndrome. J Clin Endocrinol Metab 2012;97:E2271-6. doi:10.1210/jc.2012-1996
50 Katayama M, Wiklander OPB, Fritz T, et al. Circulating exosomal miR-20b-5p is elevated in type 2 diabetes and could impair insulin action in human skeletal muscle. Diabetes 2019;68:515–26. doi:10.2337/db18-0470
51 Khan R, Kadamkode V, Kesharwani D, et al. Circulatory miR-98-5p levels are deregulated during diabetes and it inhibits proliferation and promotes apoptosis by targeting PPP1R15B in keratinocytes. RNA Biol 2020;17:188–201. doi:10.1080/15476286.2019.1673117
52 Klöting N, Berthold S, Kovacs P, et al. MicroRNA expression in human omental and subcutaneous adipose tissue. PLoS One 2009;4:e4699. doi:10.1371/journal.pone.0004699
53 Kokkinopoulou I, Maratou E, Mitrou P, et al. Decreased expression of microRNAs targeting type-2 diabetes susceptibility genes in peripheral blood of patients and predisposed individuals. Endocrine 2019;66:226–39. doi:10.1007/s12020-019-02062-0
54 Kong L, Han W, Jiang X, et al. Expression and clinical significance of peripheral miR-34a during the onset of type 2 diabetes. J Shangdong Univ (Health Sci 2010;48:2–4.
55 Kong L, Zhu J, Han W, et al. Significance of serum microRNAs in pre-diabetes and newly diagnosed type 2 diabetes: a clinical study. Acta Diabetol 2011;48:61–9. doi:10.1007/s00592-010-0226-0
56 Krause C, Geißler C, Tackenberg H, et al. Multi-layered epigenetic regulation of IRS2 expression in the liver of obese individuals with type 2 diabetes. Diabetologia Published Online First: 2020. doi:10.1007/s00125-020-05212-6
57 Kuschnerus K, Straessler ET, Müller MF, et al. Increased expression of miR-483-3p impairs the vascular response to injury in type 2 diabetes. Diabetes 2019;68:349–60. doi:10.2337/db18-0084
58 Lareyre F, Clément M, Moratal C, et al. Differential micro-RNA expression in diabetic patients with abdominal aortic aneurysm. Biochimie 2019;162:1–7. doi:10.1016/j.biochi.2019.03.012
59 Latouche C, Natoli A, Reddy-luthmoodoo M, et al. MicroRNA-194 modulates glucose metabolism and its skeletal muscle expression is reduced in diabetes. PLoS One 2016;11:1–20. doi:10.1371/journal.pone.0155108
60 Latreille M, Hausser J, Stützer I, et al. MicroRNA-7a regulates pancreatic β cell function. J Clin Invest 2014;124:2722–35. doi:10.1172/JCI73066DS1
61 Li H, Fan J, Zhao Y, et al. Nuclear miR-320 mediates diabetes-induced cardiac dysfunction by activating transcription of fatty acid metabolic genes to cause lipotoxicity in the heart. Circ Res 2019;125:1106–20. doi:10.1161/CIRCRESAHA.119.314898
62 Li MY, Pan SR, Qiu AY. Roles of microRNA-221/222 in type 2 diabetic patients with post-menopausal breast cancer. Genet Mol Res 2016;15:1–10.
63 Li YB, Wu Q, Liu J, et al. miR-199a-3p is involved in the pathogenesis and progression of diabetic neuropathy through downregulation of SerpinE2. Mol Med Rep 2017;16:2417–24. doi:10.3892/mmr.2017.6874
64 Lian W, Hu X, Shi R, et al. MiR-31 regulates the function of diabetic endothelial progenitor cells by targeting Satb2. Acta Biochim Biophys Sin 2018;50:336–44. doi:10.1093/abbs/gmy010
65 Liang L, Stone RC, Stojadinovic O, et al. Integrative analysis of miRNA and mRNA paired expression profiling of

primary fibroblast derived from diabetic foot ulcers reveals multiple impaired cellular functions. Wound Rep Reg 2016;24:943–53. doi:10.1111/wrr.12470
66 Liang YZ, Dong J, Zhang J, et al. Identification of neuroendocrine stress response-related circulating microRNAs as biomarkers for type 2 diabetes mellitus and insulin resistance. Front Endocrinol (Lausanne) 2018;9:1–11. doi:10.3389/fendo.2018.00132
67 Liang Z, Gao KP, Wang YX, et al. RNA sequencing identified specific circulating miRNA biomarkers for early detection of diabetes retinopathy. Am J Physiol Endocrinol Metab 2018;315:374–85. doi:10.1152/ajpendo.00021.2018
68 Liu H, Xu Y, Li H, et al. Loss of microRNA-145 expression is involved in the development and prognosis of breast cancer complicated by type 2 diabetes mellitus. Int J Biol Markers 2016;31:368–74. doi:10.5301/jbm.5000220
69 Liu Y, Li H, Liu J, et al. Variations in microRNA-25 expression influence the severity of diabetic kidney disease. J Am Soc Nephrol 2017;28:3627–38. doi:10.1681/ASN.2015091017
70 Locke JM, da Silva Xavier G, Dawe HR, et al. Increased expression of miR-187 in human islets from individuals with type 2 diabetes is associated with reduced glucose-stimulated insulin secretion. Diabetologia 2014;57:122–8. doi:10.1007/s00125-013-3089-4
71 Long Y, Zhan Q, Yuan M, et al. The expression of microRNA-223 and FAM5C in cerebral infarction patients with diabetes mellitus. Cardiovasc Toxicol 2017;17:42–8. doi:10.1007/s12012-015-9354-7
72 Lu H, Buchan RJ, Cook SA. MicroRNA-223 regulates Glut4 expression and cardiomyocyte glucose metabolism. Cardiovasc Res 2010;86:410–20. doi:10.1093/cvr/cvq010
73 Luo M, Li R, Deng X, et al. Platelet-derived miR-103b as a novel biomarker for the early diagnosis of type 2 diabetes. Acta Diabetol 2015;52:943–9. doi:10.1007/s00592-015-0733-0
74 Luo M, Wang G, Xu C, et al. Circulating miR-30c as a predictive biomarker of type 2 diabetes mellitus with coronary heart disease by regulating PAI-1/VN interactions. Life Sci 2019;239:117092. doi:10.1016/j.lfs.2019.117092
75 Luo M, Xu C, Luo Y, et al. Circulating miR-103 family as potential biomarkers for type 2 diabetes through targeting CAV-1 and SFRP4. Acta Diabetol 2020;57:309–22. doi:10.1007/s00592-019-01430-6
76 Mao G, Liu L. microRNA-18a is a genetic marker for the early diagnosis of cerebral injury induced by type 2 diabetes. Exp Ther Med 2014;8:1901–5. doi:10.3892/etm.2014.1996
77 Massaro JD, Polli CD, Costa e Silva M, et al. Post-transcriptional markers associated with clinical complications in type 1 and type 2 diabetes mellitus. Mol Cell Endocrinol 2019;490:1–14. doi:10.1016/j.mce.2019.03.008
78 Massart J, Sjögren RJO, Lundell LS, et al. Altered miRNA-29 expression in type 2 diabetes influences glucose and lipid metabolism in skeletal muscle. Diabetes 2017;66:1807–18.
79 Matsha TE, Kengne AP, Hector S, et al. MicroRNA profiling and their pathways in South African individuals with prediabetes and newly diagnosed type 2 diabetes mellitus. Oncotarget 2018;9:30485–98.
80 Mazloom H, Alizadeh S, Pasalar P, et al. Downregulated microRNA-155 expression in peripheral blood mononuclear cells of type 2 diabetic patients is not correlated with increased inflammatory cytokine production. Cytokine 2015;76:403–8. doi:10.1016/j.cyto.2015.07.007
81 Meng S, Cao J-T, Zhang B, et al. Downregulation of microRNA-126 in endothelial progenitor cells from diabetes patients impairs their functional properties via target gene Spred-1. J Mol Cell Cardiol 2012;53:64–72. doi:10.1016/j.yjmcc.2012.04.003
82 Meng S, Cao J, Zhang X, et al. Downregulation of microRNA-130a contributes to endothelial progenitor cell dysfunction in diabetic patients via its target Runx3. PLoS One 2013;8:1–10. doi:10.1371/journal.pone.0068611
83 Mensà E, Giuliani A, Matacchione G, et al. Circulating miR-146a in healthy aging and type 2 diabetes: age- and gender-specific trajectories. Mech Ageing Dev 2019;180:1–10. doi:10.1016/j.mad.2019.03.001
84 Monfared YK, Honardoost M, Sarookhani MR, et al. Circulating miR-135 may serve as a novel co-biomarker of HbA1c in type 2 diabetes. Appl Biochem Biotechnol 2020;191:623–30. doi:10.1007/s12010-019-03163-2
85 Mononen N, Lyytikäinen LP, Seppälä I, et al. Whole blood microRNA levels associate with glycemic status and correlate with target mRNAs in pathways important to type 2 diabetes. Sci Rep 2019;9:1–14. doi:10.1038/s41598-019-43793-4
86 Ortega FJ, Moreno-Navarrete JM, Pardo G, et al. MiRNA expression profile of human subcutaneous adipose and during adipocyte differentiation. PLoS One 2010;5:e9022. doi:10.1371/journal.pone.0009022
87 Ortega FJ, Mercader JM, Moreno-Navarrete JM, et al. Profiling of circulating microRNAs reveals common microRNAs linked to type 2 diabetes that change with insulin sensitization. Diabetes Care 2014;37:1375–83. doi:10.2337/dc13-1847
88 Ou L, Sun T, Cheng Y, et al. MicroRNA-214 contributes to regulation of necroptosis via targeting ATF4 in diabetes-associated periodontitis. J Cell Biochem 2019;120:14791–803. doi:10.1002/jcb.28740
89 Párrizas M, Brugnara L, Esteban Y, et al. Circulating miR-192 and miR-193b are markers of prediabetes and are modulated by an exercise intervention. Endocrinol Metab 2015;100:407–15. doi:10.1210/jc.2014-2574
90 Parrizas M, Mundet X, Castaño C, et al. miR-10b and miR-223-3p in serum microvesicles signal progression from prediabetes to type 2 diabetes. J Endocrinol Invest 2020;43:451–9. doi:10.1007/s40618-019-01129-z
91 Pek SLT, Sum CF, Lin MX, et al. Circulating and visceral adipose miR-100 is down-regulated in patients with obesity and type 2 diabetes. Mol Cell Endocrinol 2016;427:112–23. doi:10.1016/j.mce.2016.03.010
92 Peng R, Liu H, Peng H, et al. Promoter hypermethylation of let-7a-3 is relevant to its down-expression in diabetic nephropathy by targeting UHRF1. Gene 2015;570:57–63. doi:10.1016/j.gene.2015.05.073
93 Pescador N, Pérez-Barba M, Ibarra JM, et al. Serum circulating microRNA profiling for identification of potential type 2 diabetes and obesity biomarkers. PLoS One 2013;8:e77251. doi:10.1371/journal.pone.0077251
94 Poddar S, Kesharwani D, Datta M. Histone deacetylase inhibition regulates miR-449a levels in skeletal muscle cells. Epigenetics 2016;11:579–87. doi:10.1080/15592294.2016.1188247
95 Polina ER, Oliveira FM, Sbruzzi RC, et al. Gene polymorphism and plasma levels of miR-155 in diabetic retinopathy. Endocr Connect 2019;8:1591–9. doi:10.1530/EC-19-0446
96 Prabu P, Rome S, Sathishkumar C, et al. Circulating miRNAs of 'Asian Indian phenotype' identified in subjects with impaired glucose tolerance and patients with type 2 diabetes. PLoS One 2015;10:1–14. doi:10.1371/journal.pone.0128372
97 Prabu P, Poongothai S, Shanthirani CS, et al. Altered circulatory levels of miR-128, BDNF, cortisol and shortened telomeres in patients with type 2 diabetes and depression. Acta Diabetol 2020;57:799–807. doi:10.1007/s00592-020-01486-9
98 Radović N, Jakoba NN, Petrovic N, et al. MicroRNA-146a and microRNA-155 as novel crevicular fluid biomarkers for

periodontitis in non-diabetic and type 2 diabetic patients. J Clin Periodontol 2018;45:663–71.
99 Ramirez HA, Liang L, Pastar I, et al. Comparative genomic, microRNA, and tissue analyses reveal subtle differences between non-diabetic and diabetic foot skin. PLoS One 2015;10:1–19. doi:10.1371/journal.pone.0137133
100 Rawal S, Munasinghe PE, Nagesh PT, et al. Down-regulation of miR-15a/b accelerates fibrotic remodelling in the type 2 diabetic human and mouse heart. Clin Sci 2017;131:847–63. doi:10.1042/CS20160916
101 Rawal S, Nagesh PT, Coffey S, et al. Early dysregulation of cardiac-specific microRNA-208a is linked to maladaptive cardiac remodelling in diabetic myocardium. Cardiovasc Diabetol 2019;18:1–12. doi:10.1186/s12933-019-0814-4
102 Regmi A, Liu G, Zhong X, et al. Evaluation of serum micrornas in patients with diabetic kidney disease: a nested case-controlled study and bioinformatics analysis. Med Sci Monit 2019;25:1699–708. doi:10.12659/MSM.913265
103 Rezk NA, Sabbah NA, Saad MSS. Role of microRNA 126 in screening, diagnosis, and prognosis of diabetic patients in Egypt. IUBMB Life 2016;68:452–8. doi:10.1002/iub.1502
104 Riches K, Alshanwani AR, Warburton P, et al. Elevated expression levels of miR-143/5 in saphenous vein smooth muscle cells from patients with type 2 diabetes drive persistent changes in phenotype and function. J Mol Cell Cardiol 2014;74:240–50. doi:10.1016/j.yjmcc.2014.05.018
105 Rojas LB, Weigelt K, Wit H De, et al. Study on inflammation - related genes and microRNAs, with special emphasis on the vascular repair factor HGF and miR-574-3p, in monocytes and serum of patients with T2D. Diabetol Metab Syndr 2016;8:6. doi:10.1186/s13098-015-0113-5
106 Rong Y, Bao W, Shan Z, et al. Increased microRNA-146a levels in plasma of patients with newly diagnosed type 2 diabetes mellitus. PLoS One 2013;8:e73272. doi:10.1371/journal.pone.0073272
107 Rubie C, Zimmer J, Lammert F, et al. MicroRNA-496 and Mechanistic Target of Rapamycin Expression are Associated with Type 2 Diabetes Mellitus and Obesity in Elderly People. Ann Nutr Metab 2019;74:279–86. doi:10.1159/000499576
108 Santovito D, Nardis V De, Marcantonio P, et al. Plasma exosome microRNA profiling unravels a new potential modulator of adiponectin pathway in diabetes: effect of glycemic control. J Clin Endocrinol Metab 2014;99:1681–5. doi:10.1210/jc.2013-3843
109 Sebastiani G, Po A, Miele E, et al. MicroRNA-124a is hyperexpressed in type 2 diabetic human pancreatic islets and negatively regulates insulin secretion. Acta Diabetol 2015;52:523–30. doi:10.1007/s00592-014-0675-y
110 Silva MED da C e, Polina ER, Crispim D, et al. Plasma levels of miR ‐ 29b and miR ‐ 200b in type 2 diabetic retinopathy. J Cell Mol Med 2019;23:1280 ‐ 7. doi:10.1111/jcmm.14030
111 Smit-McBride Z, Nguyen AT, Yu AK, et al. Unique molecular signatures of microRNAs in ocular fluids and plasma in diabetic retinopathy. PLoS One 2020;15:e0235541. doi:10.1371/journal.pone.0235541
112 Spinetti G, Fortunato O, Caporali A, et al. MicroRNA-15a and microRNA-16 impair human circulating proangiogenic cell functions and are increased in the proangiogenic cells and serum of patients with critical limb ischemia. Circ Res 2013;112:335–46. doi:10.1161/CIRCRESAHA.111.300418.figure
113 Spinetti G, Sangalli E, Tagliabue E, et al. MicroRNA-21/PDCD4 proapoptotic signaling from circulating CD34+ cells to vascular endothelial cells: a potential contributor to adverse cardiovascular outcomes in patients with critical limb ischemia. Diabetes Care 2020;43:1520–9. doi:10.2337/dc19-2227
114 Stępień EL, Durak-Kozica M, Kamińska A, et al. Circulating ectosomes: determination of angiogenic microRNAs in type 2 diabetes. Theranostics 2018;8:3874–90. doi:10.7150/thno.23334
115 Sucharita S, Ashwini V, Prabhu JS, et al. The role of circulating microRNA in the regulation of beta cell function and insulin resistance among Indians with type 2 diabetes. Indian J Endocrinol Metab 2018;22:770–3. doi:10.4103/ijem.IJEM
116 Sun K, Chang X, Yin L, et al. Expression and DNA methylation status of microRNA-375 in patients with type 2 diabetes mellitus. Mol Med Rep 2014;9:967–72. doi:10.3892/mmr.2013.1872
117 Sun Q, Zeng J, Liu Y, et al. microRNA-9 and -29a regulate the progression of diabetic peripheral neuropathy via ISL1-mediated sonic hedgehog signaling pathway. Aging (Albany NY) 2020;12:11446–65. doi:10.18632/aging.103230
118 Tang W Bin, Zheng L, Yan R, et al. MiR302a-3p may modulate renal epithelial-mesenchymal transition in diabetic kidney disease by targeting ZEB1. Nephron 2018;138. doi:10.1159/000481465
119 Togliatto G, Dentelli P, Rosso A, et al. PDGF-BB carried by endothelial cell–derived extracellular vesicles reduces vascular smooth muscle cell apoptosis in diabetes. Diabetes 2018;67:704–16. doi:10.2337/db17-0371
120 Wang C, Wan S, Yang T, et al. Increased serum microRNAs are closely associated with the presence of microvascular complications in type 2 diabetes mellitus. Sci Rep 2016;6:20032. doi:10.1038/srep20032
121 Wang H, Lin Y, Zhou Y, et al. Circulatory level of microRNA 92a in patients of stable angina pectoris with diabetes mellitus. China J Mod Med 2012;22:26–9.
122 Wang H, Wang Z, Tang Q. Reduced expression of microRNA-199a-3p is associated with vascular endothelial cell injury induced by type 2 diabetes mellitus. Exp Ther Med 2018;16:3639–45. doi:10.3892/etm.2018.6655
123 Wang JM, Tao J, Chen DD, et al. MicroRNA miR-27b rescues bone marrow-derived angiogenic cell function and accelerates wound healing in type 2 diabetes mellitus. Arter Thromb Vasc Biol 2014;34:99–109. doi:10.1161/ATVBAHA.113.302104
124 Wang S, Ai H, Liu L, et al. Micro-RNA-27a/b negatively regulates hepatic gluconeogenesis by targeting FOXO1. Am J Physiol Endocrinol Metab 2019;317:E911–24. doi:10.1152/ajpendo.00190.2019
125 Wang SS, Li YQ, Liang YZ, et al. Expression of miR-18a and miR-34c in circulating monocytes associated with vulnerability to type 2 diabetes mellitus and insulin resistance. J Cell Mol Med 2017;XX:1–9. doi:10.1111/jcmm.13240
126 Wang WY, Zheng YS, Li ZG, et al. MiR-92a contributes to the cardiovascular disease development in diabetes mellitusthrough NF-κB and downstream inflammatory pathways. Eur Rev Med Pharmacol Sci 2019;23:3070–9. doi:10.26355/eurrev_201904_17589
127 Wang X, Sundquist J, Zöller B, et al. Determination of 14 circulating microRNAs in Swedes and Iraqis with and without type 2 diabetes mellitus. PLoS One 2014;9:e86792. doi:10.1371/journal.pone.0086792
128 Widlansky ME, Jensen DM, Wang J, et al. MiR-29 contributes to normal endothelial function and can restore it in cardiometabolic disorders. EMBO Mol Med 2018;10:1–14. doi:10.15252/emmm.201708046
129 Witkowski M, Witkowski M, Saffarzadeh M, et al. Vascular miR-181b controls tissue factor-dependent thrombogenicity and inflammation in type 2 diabetes. Cardiovasc Diabetol 2020;19:1–12. doi:10.1186/s12933-020-0993-z
130 Wu L, Dai X, Zhan J, et al. Profiling peripheral microRNAs in obesity and type 2 diabetes mellitus. APMIS

2015;123:580–5. doi:10.1111/apm.12389
131 Xavier DJ, Takahashi P, Evangelista AF, et al. Assessment of DNA damage and mRNA/miRNA transcriptional expression profiles in hyperglycemic versus non-hyperglycemic patients with type 2 diabetes mellitus. Mutat Res 2015;776:98–110. doi:10.1016/j.mrfmmm.2015.01.016
132 Xiang Y, Cheng J, Wang D, et al. Hyperglycemia repression of miR-24 coordinately upregulates endothelial cell expression and secretion of von Willebrand factor. Blood 2015;125:3377–88. doi:10.1182/blood-2015-01-620278.The
133 Xu Q, Meng S, Liu B, et al. MicroRNA-130a regulates autophagy of endothelial progenitor cells through Runx3. Clin Exp Pharmacol Physiol 2014;41:351–7. doi:10.1111/1440-1681.12227
134 Yan S, Wang T, Huang S, et al. Differential expression of microRNAs in plasma of patients with prediabetes and newly diagnosed type 2 diabetes. Acta Diabetol 2016;53:693–702. doi:10.1007/s00592-016-0837-1
135 Yan YX, Xiao HB, Zhang J, et al. Pri-miR-144 rs9279 is associated with type 2 diabetes and regulation of stress response. J Cell Physiol Published Online First: 2020. doi:10.1002/jcp.29883
136 Yang F, Chen Y, Xue Z, et al. High-throughput sequencing and exploration of the lncRNA-circRNA-miRNA-mRNA network in type 2 diabetes mellitus. Biomed Res Int 2020;2020. doi:10.1155/2020/8162524
137 Yang S, Chen X, Yang M, et al. The variant at TGFBRAP1 is significantly associated with type 2 diabetes mellitus and affects diabetes‐related miRNA expression. J Cell Mol Med 2019;23:83‐92. doi:10.1111/jcmm.13885
138 Yang TT, Song S, Xue H, et al. Regulatory T cells in the pathogenesis of type 2 diabetes mellitus retinopathy by miR-155. Eur Rev Med Pharmacol Sci 2015;19:2010–5.
139 Yang X, Liu S, Zhang R, et al. MicroRNA-192 as a specific biomarker for the early diagnosis of diabetic kidney disease. J Diabetes Investig 2017;:0–26. doi:10.1111/jdi.12753
140 Yang Z, Chen H, Si H, et al. Serum miR-23a, a potential biomarker for diagnosis of pre-diabetes and type 2 diabetes. Acta Diabetol 2014;51:823–31. doi:10.1007/s00592-014-0617-8
141 Yang ZM, Chen LH, Hong M, et al. Serum microRNA profiling and bioinformatics of patients with spleen-deficiency syndrome. Evid Based Complement Altern Med 2016;2016:1–9. doi:10.1155/2016/8726720
142 Yang ZM, Chen LH, Hong M, et al. Serum microRNA profiling and bioinformatics analysis of patients with type 2 diabetes mellitus in a Chinese population. Mol Med Rep 2017;15:2143–53. doi:10.3892/mmr.2017.6239
143 Ye M, Li D, Yang J, et al. MicroRNA-130a targets MAP3K12 to modulate diabetic endothelial progenitor cell function. Cell Physiol Biochem 2015;36:712–26. doi:10.1159/000430132
144 Yi B, Huang J, Zhang W, et al. Vitamin D receptor down-regulation is associated with severity of albuminuria in type 2 diabetes patients. J Clin Endocrinol Metab 2016;101:4395–404. doi:10.1210/jc.2016-1516
145 Yin C, Lin X, Sun Y, et al. Dysregulation of miR-210 is involved in the development of diabetic retinopathy and serves a regulatory role in retinal vascular endothelial cell proliferation. Eur J Med Res 2020;25:1–8. doi:10.1186/s40001-020-00416-3
146 Zampetaki A, Kiechl S, Drozdov I, et al. Plasma microRNA profiling reveals loss of endothelial miR-126 and other microRNAs in type 2 diabetes. Circ Res 2010;107:810–7. doi:10.1161/CIRCRESAHA.110.226357
147 Zhang J, Sun X, Chen J, et al. Increasing the miR-126 expression in the peripheral blood of patients with diabetic foot ulcers treated with maggot debridement therapy. J Diabetes Complications 2017;31:241–4. doi:10.1016/j.jdiacomp.2016.07.026
148 Zhang JY, Gong YL, Li CJ, et al. Circulating miRNA biomarkers serve as a fingerprint for diabetic atherosclerosis. Am J Transl Res 2016;8:2650–8.
149 Zhang T, Lv C, Li L, et al. Plasma miR-126 is a potential biomarker for early prediction of type 2 diabetes mellitus in susceptible individuals. Biomed Res Int 2013;2013:761617. doi:10.1155/2013/761617
150 Zhang T, Li L, Shang Q, et al. Circulating miR-126 is a potential biomarker to predict the onset of type 2 diabetes mellitus in susceptible individuals. Biochem Biophys Res Commun 2015;463:60–3. doi:10.1016/j.bbrc.2015.05.017
151 Zhang Y, Guan Q, Jin X. Platelet-derived miR-92a downregulates cysteine protease inhibitor cystatin C in type II diabetic lower limb ischemia. Exp Ther Med 2015;9:2257–62. doi:10.3892/etm.2015.2400
152 Zhao B, Li H, Liu J, et al. MicroRNA-23b targets Ras GTPase-activating protein SH3 domain-binding protein 2 to alleviate fibrosis and albuminuria in diabetic nephropathy. J Am Soc Nephrol 2016;27:2597–608. doi:10.1681/ASN.2015030300
153 Zhao H, Guan J, Lee H-M, et al. Up-regulated pancreatic tissue microRNA-375 associates with human type 2 diabetes through beta-cell deficit and islet amyloid deposition. Pancreas 2010;39:843–6. doi:10.1097/MPA.0b013e3181d12613
154 Zhou J, Peng R, Li T, et al. A potentially functional polymorphism in the regulatory region of let-7a-2 is associated with an increased risk for diabetic nephropathy. Gene 2013;527:456–61. doi:10.1016/j.gene.2013.06.088
155 Zhu J, Wang C, Zhang X, et al. Correlation analysis of microRNA155 and microRNA29 with T2DM and prediction and verification of target genes. J Diabetes Investig Published Online First: 2020. doi:10.1111/jdi.13334
156 Zou HL, Wang Y, Gang Q, et al. Plasma level of miR-93 is associated with higher risk to develop type 2 diabetic retinopathy. Graefe's Arch Clin Exp Ophthalmol 2017;255:1159–66. doi:10.1007/s00417-017-3638-5
